# Stanniocalcin 2 governs cancer cell adaptation to nutrient insufficiency through alleviation of oxidative stress

**DOI:** 10.1038/s41419-024-06961-7

**Published:** 2024-08-06

**Authors:** Shuo Qie, Haijuan Xiong, Yaqi Liu, Chenhui Yan, Yalei Wang, Lifeng Tian, Chenguang Wang, Nianli Sang

**Affiliations:** 1 Department of Pathology, Tianjin Medical University Cancer Institute and Hospital, Tianjin, China; 2 National Clinical Research Center for Cancer, Tianjin, China; 3 Key Laboratory of Cancer Prevention and Therapy (Tianjin), Tianjin, China; 4 Tianjin’s Clinical Research Center for Cancer, Tianjin, China; 5 Department of Cancer Biology, Kimmel Cancer Center, Thomas Jefferson University, Philadelphia, PA USA; 6 Department of Biology, Drexel University, Philadelphia, PA USA

**Keywords:** Cancer metabolism, Apoptosis

## Abstract

Solid tumours often endure nutrient insufficiency during progression. How tumour cells adapt to temporal and spatial nutrient insufficiency remains unclear. We previously identified *STC2* as one of the most upregulated genes in cells exposed to nutrient insufficiency by transcriptome screening, indicating the potential of STC2 in cellular adaptation to nutrient insufficiency. However, the molecular mechanisms underlying STC2 induction by nutrient insufficiency and subsequent adaptation remain elusive. Here, we report that STC2 protein is dramatically increased and secreted into the culture media by Gln-/Glc- deprivation. *STC2* promoter contains *cis*-elements that are activated by ATF4 and p65/RelA, two transcription factors activated by a variety of cellular stress. Biologically, STC2 induction and secretion promote cell survival but attenuate cell proliferation during nutrient insufficiency, thus switching the priority of cancer cells from proliferation to survival. Loss of STC2 impairs tumour growth by inducing both apoptosis and necrosis in mouse xenografts. Mechanistically, under nutrient insufficient conditions, cells have increased levels of reactive oxygen species (ROS), and lack of STC2 further elevates ROS levels that lead to increased apoptosis. RNA-Seq analyses reveal STC2 induction suppresses the expression of monoamine oxidase B (MAOB), a mitochondrial membrane enzyme that produces ROS. Moreover, a negative correlation between STC2 and MAOB levels is also identified in human tumour samples. Importantly, the administration of recombinant STC2 to the culture media effectively suppresses MAOB expression as well as apoptosis, suggesting STC2 functions in an autocrine/paracrine manner. Taken together, our findings indicate that nutrient insufficiency induces STC2 expression, which in turn governs the adaptation of cancer cells to nutrient insufficiency through the maintenance of redox homoeostasis, highlighting the potential of STC2 as a therapeutic target for cancer treatment.

## Introduction

The major characteristics of tumour metabolism include the Warburg effect, active glutaminolysis and hypoxia [1–3]; as such, tumour cells have increased demand of glucose (Glc), glutamine (Gln) and oxygen to keep cell survival and proliferation [4–6]. However, solid tumours often have poor circulation that leads to an insufficient supply of Glc, Gln and oxygen; therefore, to investigate the mechanisms of how tumour cells respond to nutrient insufficiency could provide promising therapeutic targets. Our previous study identified *Stanniocalcin 2* (*STC2*) as one of the most upregulated genes upon Gln-/Glc- deprivation and hypoxia exposure [1, 7], suggesting a critical role of STC2 in the cellular response to nutrient insufficiency.

STC2 belongs to the secreted glycoprotein hormone family that was reported to lower intracellular calcium concentrations by attenuating Ca^2+^ influx [8, 9]. Under physiological conditions, STC2 is highly expressed in skeletal muscle, heart, testis and pancreas [8, 10]; while under pathological conditions, STC2 is upregulated in a plethora of human tumours, including hepatocellular carcinoma (HCC), cervical cancer, nasopharyngeal carcinoma, colorectal cancer, gastric cancer, oesophageal squamous cell carcinoma, prostate cancer, renal cell carcinoma, etc. [7, 8, 11, 12]. Increased STC2 levels are strongly correlated with tumour development, progression and poor prognosis for most human tumours except breast cancer [13]. The expression of STC2 is regulated at both transcriptional and post-transcriptional levels, particularly under stress conditions, including ER stress, hypoxia, nutrient insufficiency and radiation-mediated stress [1, 7, 8, 11, 14]. Biologically, STC2 is involved in many biological processes in human tumours, such as cell survival, proliferation, migration and immune escape [8, 15–17]. However, the precise mechanisms underlying the stress-induced STC2 induction remain unclear.

Previous reports reveal hydrogen peroxide (H_2_O_2_) induces STC2 expression, and loss of STC2 impairs the tolerability of cells to oxidative stress [18–20], suggesting a potential mechanism that links nutrient insufficiency to STC2 induction, redox homoeostasis and cellular adaption. Current understanding is that aberrant metabolic activities of tumour cells result in an elevation of reactive oxygen species (ROS) [21]. Low to moderate levels of ROS may serve as a signalling mechanism to maintain cellular physiology; increased levels of ROS facilitate tumorigenesis by causing genetic alteration and oncogenic transformation; however, long-term and/or excessive ROS may trigger apoptosis [22]. In general, the glutathione (GSH) and nicotinamide adenine dinucleotide phosphate (NADPH) systems work coordinatively to maintain intracellular redox homoeostasis [23, 24]. As for tumour cell metabolism, Gln serves as the major source of glutamate that functions as an important substrate for de novo GSH biosynthesis; hence, Gln-deprivation results in decreased GSH levels and ROS accumulation [25, 26]. Glc insufficiency may trigger oxidative stress via impaired NADPH production, because Glc is the substrate for the pentose phosphate pathway, the major pathway to generate NADPH [27]. Moreover, hypoxia impairs the electron transport chain (ETC) activity and also enhances ROS production [28, 29]. Therefore, deprivation of each of these major nutrients may trigger oxidative stress, and cells must overcome the oxidative stress to maintain survival. A better understanding of how STC2 regulates the adaptation of tumour cells to nutrient insufficiency and the associated oxidative stress may provide new therapeutic targets.

In this study, we first confirm that STC2 protein is induced and secreted into the culture media by nutrient insufficiency. We next present data to define the *cis*-elements in the *STC2* promoter that physically interact with activating transcription factor 4 (ATF4) and nuclear factor-kappa B p65 (p65/RelA) during nutrient insufficiency. We show that STC2 induction inhibits apoptosis but attenuates tumour cell proliferation during nutrient insufficiency. In addition, loss of STC2 leads to elevated ROS levels accompanied by apoptosis during nutrient insufficiency. We finally show that STC2 induction suppresses the expression of monoamine oxidase B (MAOB), a mitochondrial membrane-associated enzyme that produces ROS, and the administration of recombinant human STC2 to culture media is sufficient to suppress MAOB expression. Taken together, our findings indicate that STC2 serves as a key regulator of redox homoeostasis that governs the survival of tumour cells during nutrient insufficiency.

## Materials and methods

### Ethical approval

Animal studies were performed according to the protocol approved by the Laboratory Animal Ethics Committee at the Tianjin Medical University Cancer Institute & Hospital (TMUCIH) (#: AE-2022036). This study has complied with all relevant ethical regulations for animal experiments. All efforts were made to minimise the numbers and suffering of experimental animals.

Human HCC tissue microarray (TMA) was purchased from the Shanghai Outdo Biotechnology (China) with an Internal Review Board approval (YB M-05-02). The clinical samples (23 HCC specimens) were collected from the Department of Pathology at the TMUCIH from January 1, 2018 to December 31, 2018. All patients were provided with informed consent and the study was conducted according to a protocol approved by the Internal Review Board at the TMUCIH (bc2021308).

### Cell culture

Hep3B, HeLa, MCF-7, SK-BR-3, MDA-MB-453, MDA-MB-468 and HEK293T (ATCC) cells were maintained in DMEM with 10% foetal bovine serum (FBS) supplemented with Pen/Strep. All cells were cultured in a humidified incubator at 37 °C with 5% CO_2_. MM01 cells were established through the extended culture of Hep3B cells in Gln-free DMEM supplemented with 10% dialysed FBS (Atlantic Biologicals, dialysed 10 kDa), 0.8 mM ammonia and Pen/Strep [30]. The culture media for MM01 cells were changed every 2 days, and the cells were passed once reaching ~90% confluency. All cells were tested routinely to ensure mycoplasma-free using Polymerase Chain Reaction (PCR) methods.

### Tumour xenografts

The NTG and athymic nude, nu/nu mice were purchased from SPF Biotechnology Co. (Beijing, China). For Hep3B xenografts, 24 NTG mice (male, age: 4 weeks old) were randomly allocated into three groups (6 per group). For HeLa xenografts, 24 athymic nude, nu/nu mice (female, age: 4 weeks old) were randomly allocated into three groups (6 per group). In general, 5 × 10^6^ Hep3B or HeLa cells were subcutaneously injected into each side of both flank regions. The xenografts were measured every three days to the completion of experiments. The growth curves were plotted with tumour sizes (calculated using the formula V_T_ = 0.5 × L × W^2^, mm^3^). After euthanization, dissected xenografts were weighted and recorded for further analysis. The mice were euthanized if the xenograft size became ≥20 mm in the long diameter, and the mice died prior to the completion of experiments were excluded for analysis.

### Statistical analysis

Statistical analyses were performed using SPSS 27.0 software (IBM SPSS Statistics). The results of qRT-PCR, tumour weight, IHC staining score, and luciferase analysis were presented as average ± standard deviation; Student *t* test or one-way ANOVA was performed to compare these results. The curves for cell proliferation and xenograft growth were compared using two-way ANOVA. Linear correlation was assessed using Spearman’s correlation analysis. Survival analysis was performed using the log rank test to estimate the survival probability, and *P* value less than 0.05 was set as statistically significant.

Addition information is provided in Supplementary Materials and Methods, and the original western blot data are included in the Original Data file.

## Results

### Nutrient insufficiency triggers STC2 induction and secretion

**Fig. 1:**
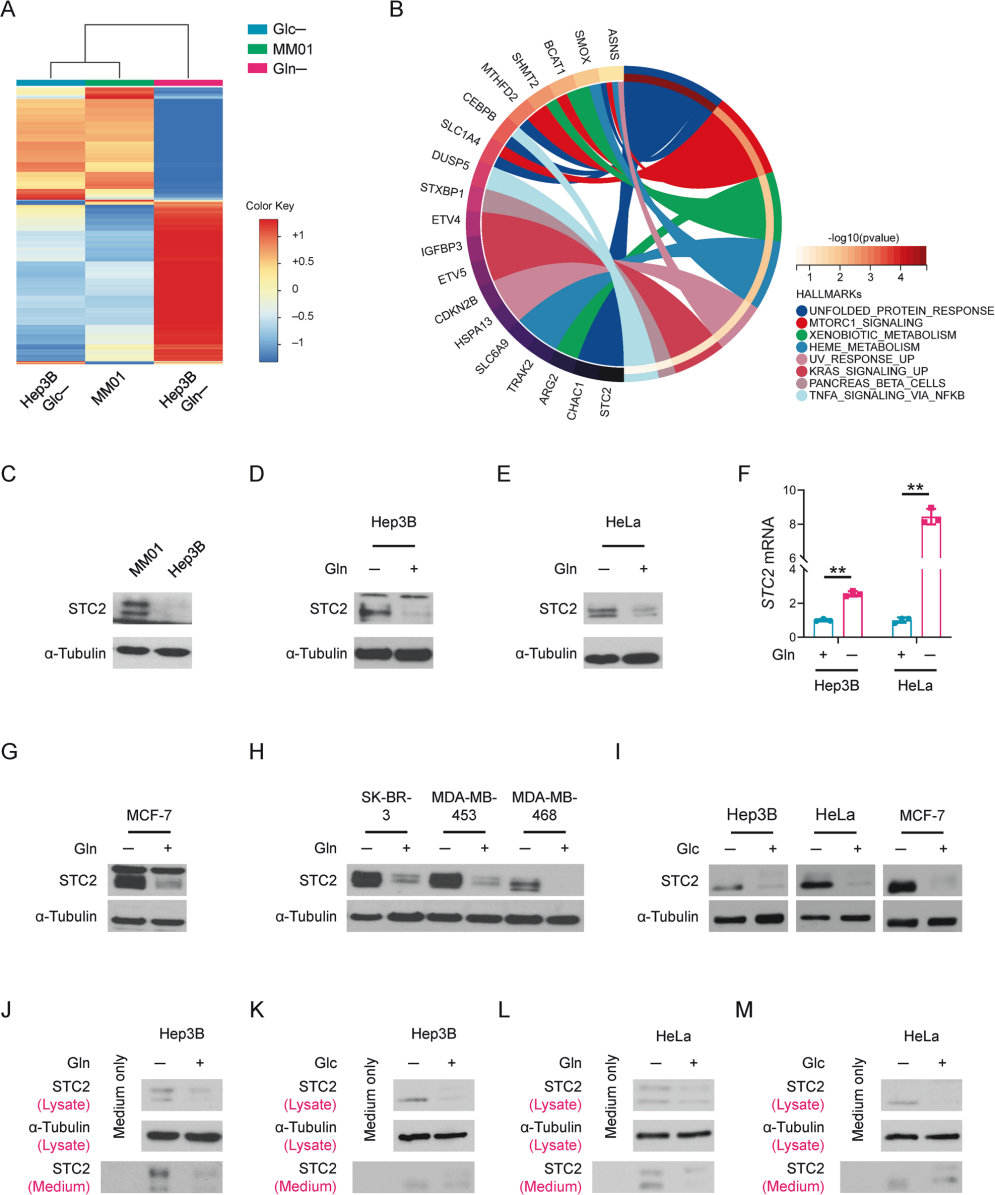
STC2 is upregulated under nutrient-insufficient conditions. **A** The iDEP analysis was performed to compare the cDNA microarray data from cells cultured with Gln-/Glc- deprived media. A total of 5783 genes are upregulated/downregulated according to the criteria | log2(fold changes) | ≥ 1. **B** Circos plot shows the biological functions of the 58 most upregulated genes in cells exposed to nutrient insufficiency. *STC2* is among the 58 most upregulated genes in MM01 cells and Gln-/Glc- deprived Hep3B cells. **C**–**E** STC2 is induced in MM01 cells (**C**), Hep3B (**D**) and HeLa (**E**) cells cultured in Gln-free media. **F** *STC2* mRNA is induced by Gln-deprivation in Hep3B and HeLa cells. Data are shown as the mean ± SD; **P < 0.01, *n* = 3. **G**, **H** STC2 is upregulated in human breast cancer cells when exposed to Gln-deprivation. **I** Glc-deprivation also upregulates STC2 in Hep3B, HeLa and MCF-7 cells. Note the loss of the high molecular weight band of STC2 in Glc-deprived cells (represents glycosylated STC2, unpublished data). **J**–**M** Nutrient insufficiency upregulates intracellular STC2 and its secretion into culture media. Both Hep3B and HeLa cells were cultured in Gln-free (**J**, **L**) or Glc-free (**K**, **M**) media, and the cell lysate and culture media were collected and analysed by Western blots.

In previous work, we cultured Hep3B cells for the long term in Gln-free media supplemented with NH_4_^+^ as an alternative nitrogen source and established a long-term Gln-deprived cell line, MM01, which maintains a slow proliferation phenotype [30]. In addition, we cultured Hep3B cells in Gln-/Glc- free media for short term that served as a model for acute nutrient insufficiency. cDNA microarray analysis revealed that *STC2* was among the most upregulated 58 genes shared by cells enduring acute Gln-/Glc- deprivation as well as long-term Gln-deprived condition (Fig. 1A, B, Supplementary Fig. S1A and Supplementary Table 1), indicating the potential of STC2 in mediating cellular adaptation to nutrient insufficiency. Further functional analyses suggested that most of these 58 genes were related to amino acid metabolism (Supplementary Fig. S1B). Moreover, our prior study found STC2 was also upregulated by hypoxia [7], suggesting STC2 induction is a common phenomenon when tumour cells exposed to nutrient insufficiency. In accordance, elevated STC2 protein levels were confirmed in MM01 cells relative to that in Hep3B cells (Fig. 1C), and STC2 was remarkably induced by acute Gln-deprivation (Fig. 1D–F and Supplementary Fig. S1C), whereas re-supplementation with Gln rapidly suppressed STC2 expression (Supplementary Fig. S1D). Similar results were observed when Gln was added back to MM01 cells (Supplementary Fig. S1E). To address whether STC2 upregulation is a common phenomenon in different tumour types enduring nutrient insufficiency, we exposed breast cancer cells to Gln-deprivation as well. We observed that STC2 was similarly induced in MCF-7, SK-BR-3, MDA-MB-453 and MDA-MB-468 cells by Gln-free media (Fig. 1G, H). In consistent with the cDNA microarray, Glc-deprivation also increased STC2 protein levels (Fig. 1I).

To test whether STC2 is induced by the lack of Gln itself or by a lack of metabolic derivatives of Gln, we administered the glutaminase inhibitor, DON, to Hep3B cells. We observed that DON treatment efficiently induced STC2 expression in Gln-rich media (Supplementary Fig. S1F, G), indicating that lack of Gln-derived metabolites is sufficient to trigger STC2 induction as well. As an inhibitor of protein secretion, BFA increased intracellular STC2 levels while decreased STC2 levels in the culture media (Supplementary Fig. S1H), confirming STC2 as a secreted protein. To address whether increased STC2 levels in cells is the result of secretion inhibition, we analysed STC2 levels in cell lysate and their corresponding culture media. We observed that increased STC2 levels in both cell lysate and culture media in a proportional manner upon Gln-/Glc- deprivation, suggesting that increased intracellular STC2 levels are not a consequence of secretion inhibition (Fig. 1J–M). Taken together, these data suggest that nutrient insufficiency triggers STC2 induction and secretion, which represents a converging consequence in response to different nutrient-insufficient conditions.

### ATF4 and p65/RelA transcriptionally induce STC2 expression during nutrient insufficiency

Our previous studies indicated that *STC2* mRNA levels were increased in cells under Gln-/Glc- deprived or hypoxic conditions [1, 8], suggesting *STC2* is transcriptionally upregulated by nutrient insufficiency. To further delineate the underlying mechanisms, we used ActD and CHX to block gene transcription and protein translation, respectively. ActD and CHX blocked STC2 expression at both mRNA and protein levels (Supplementary Fig. S2A,B), demonstrating that STC2 transcription depends on de novo synthesis of a transcription factor. In addition, to address whether Gln-deprivation affects STC2 protein stability, we performed CHX chase assay and found that after CHX treatment, STC2 showed a similar decay rate regardless of Gln levels (Supplementary Fig. S2C,D), ruling out the possibility that Gln-deprivation stabilises STC2 protein.

**Fig. 2:**
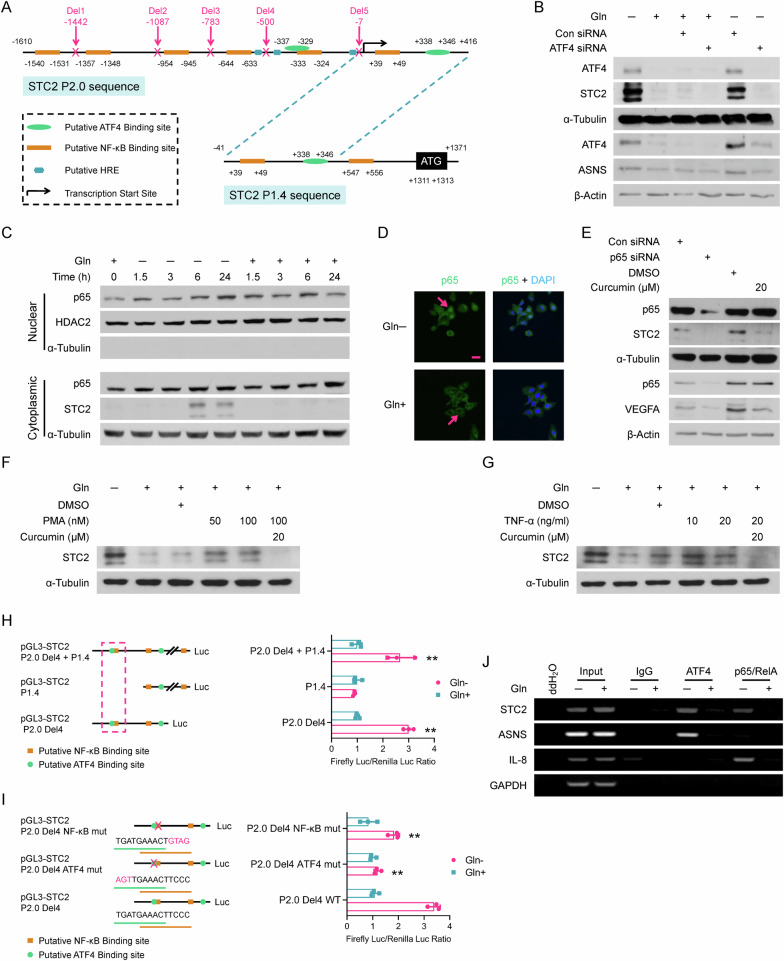
*STC2* is transcriptionally upregulated by ATF4 and NF-κB. **A** Bioinformatic analysis suggests the presence of putative ATF4, NF-κB and HRE binding sites in *STC2* promoter. **B** ATF4 knockdown abolishes STC2 induction upon Gln-deprivation. ASNS was used as a positive control for ATF4. **C** p65/RelA is translocated into the nuclei in cells exposed to Gln-free media. **D** Immunofluorescent confocal microscopic photos show nuclear localisation of p65 post Gln-deprivation. **E** p65 knockdown or inhibition by curcumin effectively suppresses STC2 induction upon Gln-deprivation. VEGFA was used as a positive control for p65. **F**, **G** Curcumin effectively suppresses STC2 induction by the NF-κB signalling activators PMA (**F**) and TNF-α (**G**). **H** Luciferase reporter assays confirm the binding sites (located at nt[−337 ~ −329] and nt[−333 ~ −324] of *STC2* promoter [NCBI Ref. #, NC_000005.10]) are required for *STC2* induction by Gln-deprivation. **I** Mutation of either ATF4 or NF-κB binding sites impairs the response of *STC2* promoter to Gln-deprivation. Data in (**H**, **I**) are shown as the mean ± SD; **P < 0.01, *n* = 3. **J** ChIP assays substantiate the direct binding of ATF4 or p65/RelA to the identified *cis*-elements on *STC2* promoter. *ASNS* serves as a positive control for ATF4; *IL-8* is used as a positive control for p65/RelA; *GAPDH* is used as a negative control.

STC2 was reported to be induced by ER stress that is correlated with ATF4 activation. ATF4 is an important component of the integrated stress response system. Amino acid insufficiency leads to the accumulation of non-aminoacylated tRNAs, which promotes the activation of general control non-derepressible-2 (GCN2) and the phosphorylation of eIF2α, thereafter triggering the selective translation of ATF4 [31, 32]. To determine whether *STC2* gene is a direct transcription target of ATF4, we performed bioinformatic analysis and found *STC2* promoter contains multiple copies of putative ATF4, NF-κB and HIF-1 binding sites (HRE) (Fig. 2A). Next, we confirmed that Gln-deprivation enhanced eIF2α phosphorylation and ATF4 induction in our experimental setting (Supplementary Fig. S2E,F). In addition, ATF4 knockdown effectively abolished STC2 induction in cells cultured in Gln-free media (Fig. 2B). Indeed, ER stress inducers like MG132 which blocks proteosome-dependent degradation of proteins, or BFA which suppresses protein secretory pathway, induced STC2 expression as well; while ActD or CHX effectively blocked this induction (Supplementary Fig. S2G–J), suggesting ER stress-triggered STC2 induction depends on de novo transcription and translation. Finally, glutaminase inhibitor, DON or Glc-deprivation also induced a co-upregulation of ATF4 and STC2 (Supplementary Fig. S2K, L).

In consistence with the existence of putative NF-κB binding sites in the *STC2* promoter, GSEA analyses highlighted the upregulation of NF-κB targets under Gln-insufficient conditions (Supplementary Fig. S3A and Supplementary Table 2). To evaluate the role of NF-κB in *STC2* induction, we performed cell fractionation analysis and immunofluorescent staining and observed that Gln-deprivation triggered p65/RelA accumulation in the nuclei (Fig. 2C, D), demonstrating the activation of p65/RelA signalling in Gln-deprived cells. Next, we applied chemical inhibitors or activators to test the importance of p65/RelA in mediating STC2 expression upon Gln-deprivation. We observed that NF-κB inhibitors, BAY 11-7082 or curcumin, efficiently blocked STC2 upregulation (Supplementary Fig. S3B–G). Furthermore, genetically suppressing p65/RelA by siRNA or IκBαSR attenuated STC2 induction by Gln-deprivation (Fig. 2E and Supplementary Fig. S3H). On the other hand, broadly used NF-κB activators, PMA or TNF-α, induced STC2 that could be blocked by curcumin (Fig. 2F, G). Taken together, these data demonstrate that p65/RelA functions as another transcription activator for STC2 induction.

To define the *cis*-elements that regulate *STC2* expression in response to Gln-deprivation, we cloned two promoter fragments into the pGL3-Luciferase reporter with a length of 2026 bp (P2.0) and 1412 bp (P1.4), respectively (Fig. 2A). Guided by the identified putative binding sites, a series of deletions were constructed and cloned into the pGL3-Luciferase reporter. Luciferase assays indicated a DNA region included in STC2-P2.0 but not in STC2-P1.4 is responsive to Gln-/Glc- deprivation or hypoxia (Supplementary Fig. S4A–C). Further deletion assays narrowed down to Deletion 4 (Del4) region that is indispensable for the response of *STC2* promoter to nutrient insufficiency (Fig. 2H and Supplementary Fig. S4D). Mutation of either ATF4 or NF-κB binding sites in this region decreased the stress response (Fig. 2I and Supplementary Fig. S4E), further confirming that these binding sites are functionally important in regulating STC2 induction. Finally, ChIP assays confirmed the direct binding of ATF4 and p65/RelA to this region of *STC2* promoter (Fig. 2J).

### STC2 induction compromises tumour cell proliferation under nutrient-insufficient conditions

**Fig. 3:**
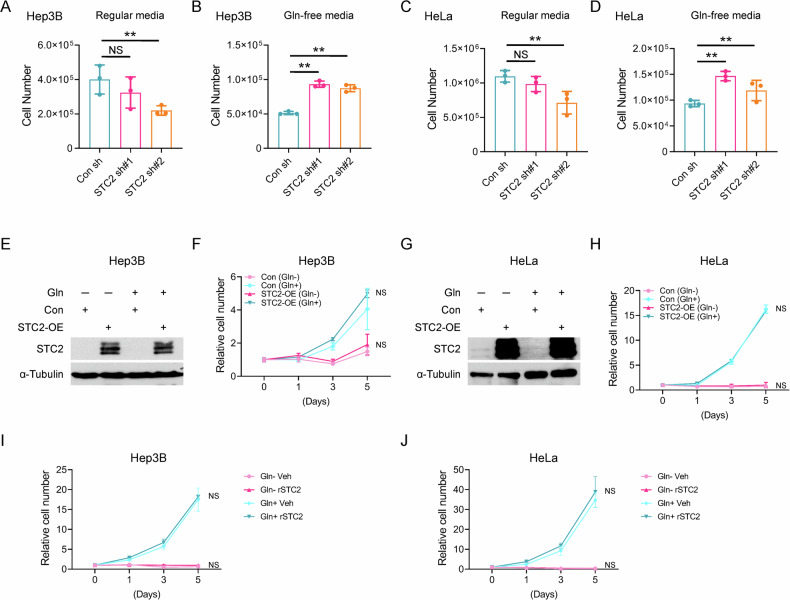
STC2 is required for optimal cell proliferation in normal culture media but negatively regulates cell proliferation upon Gln-deprivation. **A**, **B** STC2 is needed for optimal proliferation of Hep3B cells in regular media (**A**) but suppresses cell proliferation in Gln-free media (**B**). **C**, **D** STC2 is needed for optimal proliferation of HeLa cells in regular media (**C**) but suppresses cell proliferation in Gln-free media (**D**). **E**–**H** Overexpression of STC2 has no apparent effects on the proliferation of Hep3B (**E**, **F**) and HeLa (**G**, **H**) cells. **I**, **J** Exogenous rSTC2 does not affect the proliferation of Hep3B (**I**) and HeLa (**J**) cells. All data are shown as the mean ± SD; **P < 0.01; NS, not significant, *n* = 3–6.

Our previous study revealed that Gln-/Glc- deprivation caused cell cycle arrest, hence attenuating cell proliferation [1]. Since STC2 is induced by nutrient insufficiency, it is intriguing to investigate the role of STC2 in regulating cell proliferation under such conditions. We next focused on Gln-deprivation as a model to investigate the role of STC2 in regulating cell proliferation. In regular media, STC2 knockdown impaired the optimal cell proliferation (Fig. 3A, C and Supplementary Fig. S5A), while in Gln-free media, STC2 knockdown cells showed higher proliferation rates (Fig. 3B, D and Supplementary Fig. S5B), indicating that basal levels of STC2 are required to maintain the optimal proliferation when cells cultured in nutrient-rich media, whereas under nutrient insufficient conditions, elevated STC2 levels can suppress cell proliferation. Interestingly, overexpression of STC2 did not affect cell proliferation of cells cultured in either regular media or nutrient-insufficient media (Fig. 3E–H and Supplementary Fig. S5C, D). Furthermore, supplementing exogenous recombinant STC2 (rSTC2) to media did not affect cell proliferation under either conditions (Fig. 3I, J and Supplementary Fig. S5E). Taken together, these data demonstrate that basal levels of STC2 are required to support cell proliferation under nutrient-rich conditions; while endogenous *STC2* gene can be induced by nutrient insufficiency, thereby attenuating cell proliferation and facilitating cellular adaptation to stress conditions.

### STC2 induction and secretion promote tumour cell survival during nutrient insufficiency

**Fig. 4:**
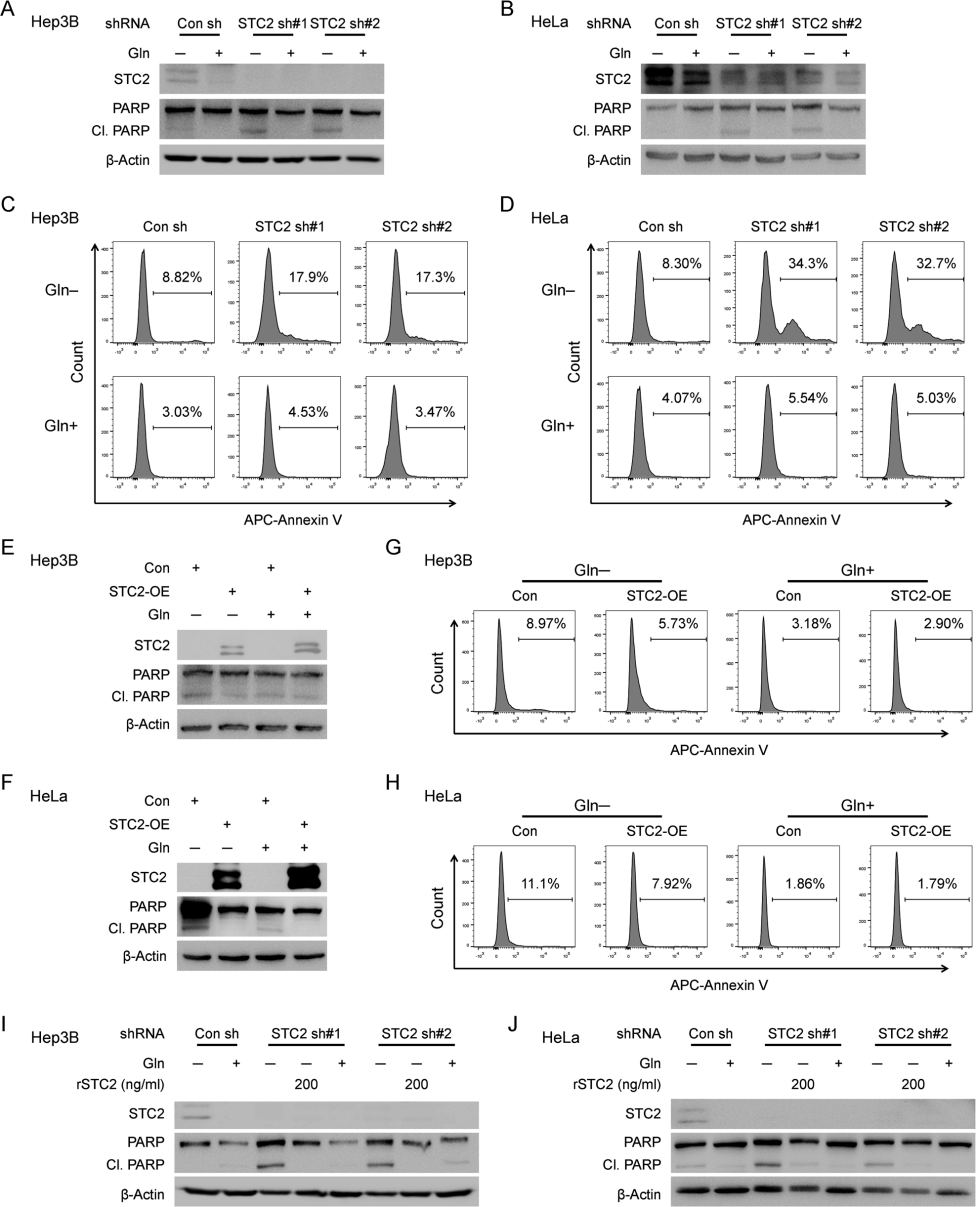
STC2 reduces apoptosis of cells under Gln-deprived conditions. **A**, **B** STC2 knockdown leads to increased PARP cleavage in Hep3B (**A**) and HeLa (**B**) cells in Gln-deprived media. **C**, **D** Flow cytometry analyses show increased apoptosis of Hep3B (**C**) and HeLa (**D**) cells as assayed by Annexin V staining. **E**, **F** Overexpression of STC2 suppresses PARP cleavage in Hep3B (**E**) and HeLa (**F**) cells cultured with Gln-free media. **G**, **H** Flow cytometry analysis indicates overexpression of STC2 inhibits apoptosis of Hep3B (**G**) and HeLa (**H**) cells cultured in Gln-deprived media as measured by Annexin V staining. **I**, **J** rSTC2 effectively decreases STC2 knockdown-triggered PARP cleavage in Hep3B (**I**) and HeLa (**J**) cells cultured in Gln-free media.

One common consequence of nutrient insufficiency and associated metabolic stress is apoptosis. We next focused on Gln-deprivation as a model to investigate whether STC2 induction played a role in maintaining cell survival during nutrient insufficiency. Western blot revealed STC2 knockdown increased PARP cleavage, a biomarker of apoptosis, under Gln-deprived conditions (Fig. 4A, B and Supplementary Fig. S6A). In accordance, flow cytometry analysis also supported that cells with STC2 knockdown were more sensitive to apoptosis triggered by Gln-deprivation (Fig. 4C, D and Supplementary Fig. S6C). On the other hand, overexpression of STC2 decreased apoptosis of cells exposed to Gln-free media (Fig. 4E–H and Supplementary Fig. S6B, D). As a secretory protein, STC2 has been proposed to function as a signalling molecule or to function intracellularly as a calcium regulator [9]. To test whether STC2 protects cells from apoptosis triggered by Gln-deprivation in an autocrine/paracrine manner, we added rSTC2 to cell culture media and found that rSTC2 efficiently suppressed PARP cleavage as well as apoptosis of cells cultured in Gln-free media (Fig. 4I, J and Supplementary Figs. S6E, F and S7). These data support that STC2 may function in an autocrine/paracrine manner to promote cell survival under nutrient insufficient conditions.

### STC2 knockdown retards the growth of mouse xenografts

**Fig. 5:**
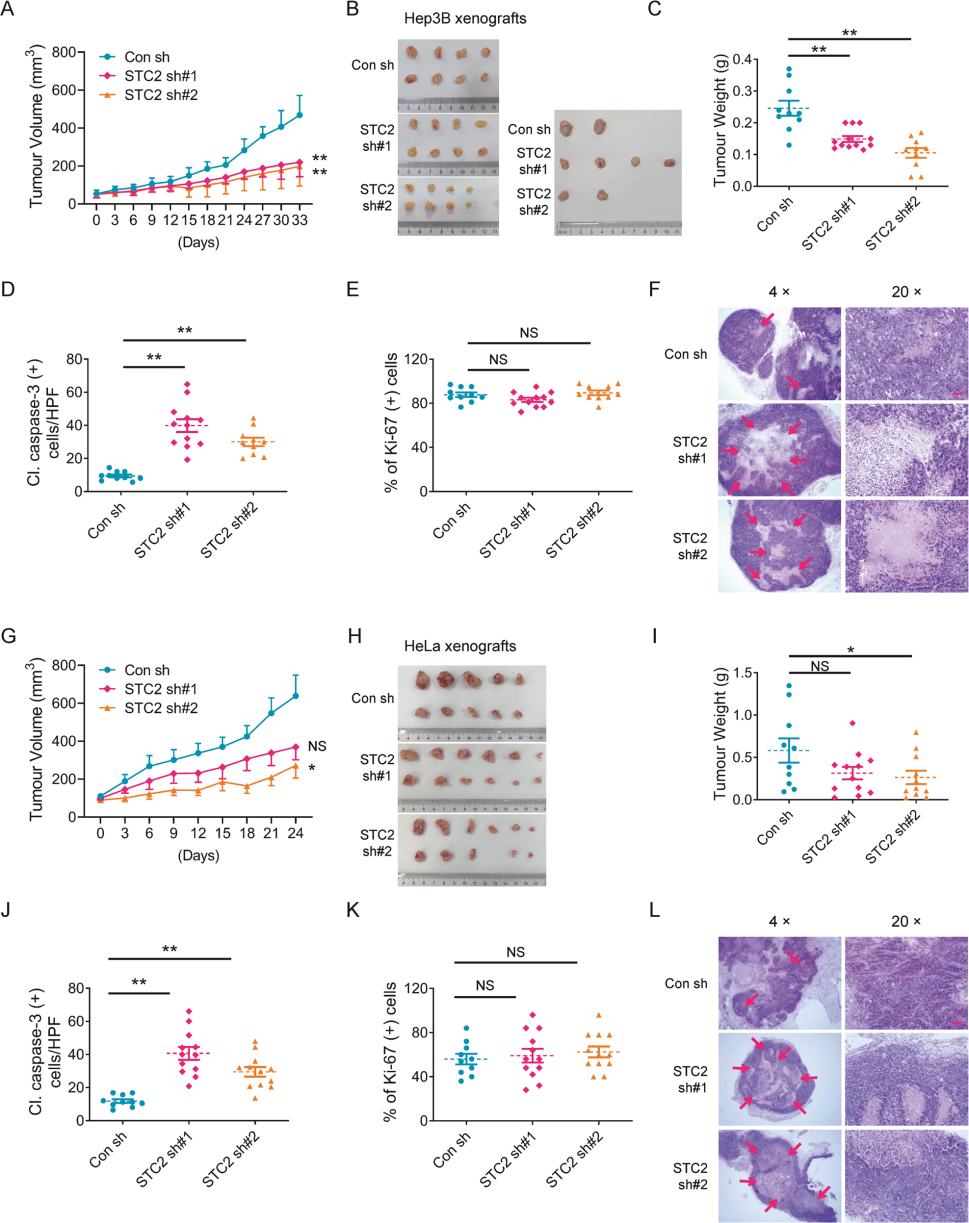
STC2 knockdown retards the growth of mouse xenografts. **A** STC2 knockdown slows down the growth of Hep3B xenografts. **B** The photographs of Hep3B tumour xenografts shown in the same scale. **C** Loss of STC2 reduces the tumour weight of Hep3B xenografts. **D** STC2 knockdown causes increased apoptosis in Hep3B xenografts indicated by caspase-3 cleavage. **E** STC2 knockdown does not affect cell proliferation rates in Hep3B xenografts as detected by Ki-67 IHC staining. **F** Loss of STC2 leads to increased necrosis in Hep3B xenografts. Red arrow, necrotic area. **G** STC2 knockdown slows down the growth of HeLa xenografts. **H** The photographs of HeLa tumour xenografts. **I** Loss of STC2 reduces the tumour weight of HeLa xenografts. **J** STC2 knockdown causes increased apoptosis in HeLa xenografts indicated by caspase-3 cleavage. **K** STC2 knockdown does not affect cell proliferation rates in HeLa xenografts evaluated by Ki-67 IHC staining. **L** Loss of STC2 leads to increased necrosis in HeLa xenografts. Red arrow, necrotic area. The representative IHC staining results in (**D**, **E**, **J**, **K**) are put in Supplementary Fig. S9. All data are shown as the mean ± SD; *P < 0.05; **P < 0.01; NS, not significant; *n* = 10–12. Scale bar, 50 μm.

To evaluate the role of STC2 in a physiological context, we first run a large-scale Kaplan-Meier Plotter analysis and found that elevated STC2 levels were associated with poor prognosis in patients with a variety of tumours including HCC, cervical squamous cell carcinoma, thymoma, oesophageal squamous cell carcinoma, renal papillary cell carcinoma, stomach adenocarcinoma, sarcoma, head and neck squamous cell carcinoma, bladder cancer and lung squamous cell carcinoma (Supplementary Fig. S8). To confirm the role of STC2 induction in tumour progression in vivo, we established mouse xenografts by injecting 5 × 10^6^ Hep3B or HeLa cells with STC2 knockdown or control shRNAs independently. We found that STC2 knockdown tumours grew slower than control ones (Fig. 5A, B, G, H). Moreover, STC2 knockdown xenografts overall had lighter tumour weights compared to control counterparts (Fig. 5C, I). To further determine the role of STC2 in cell survival and proliferation in vivo, we examined the expression of cleaved caspase-3 and Ki-67 in the dissected xenografts by IHC staining as markers of apoptosis and proliferation, respectively. We found that loss of STC2 led to increased caspase-3 cleavage, suggesting STC2 induction is a favourable factor for cell survival in vivo (Fig. 5D, J and Supplementary Fig. S9A), while there was no obvious difference in cell proliferation rates evaluated by Ki-67 IHC staining (Fig. 5E, K and Supplementary Fig. S9B). More importantly, larger necrotic areas were observed in STC2 knockdown xenografts compared to control ones (Fig. 5F, L), suggesting a failed adaptation to insufficient blood supply during tumour progression, which is consistent with a previous report that STC2 was involved in tumour angiogenesis [33]. These data highlight that STC2 plays a critical role in preventing cell death during tumour progression in vivo.

### STC2 knockdown results in ROS elevation in cells under nutrient-insufficient conditions

**Fig. 6:**
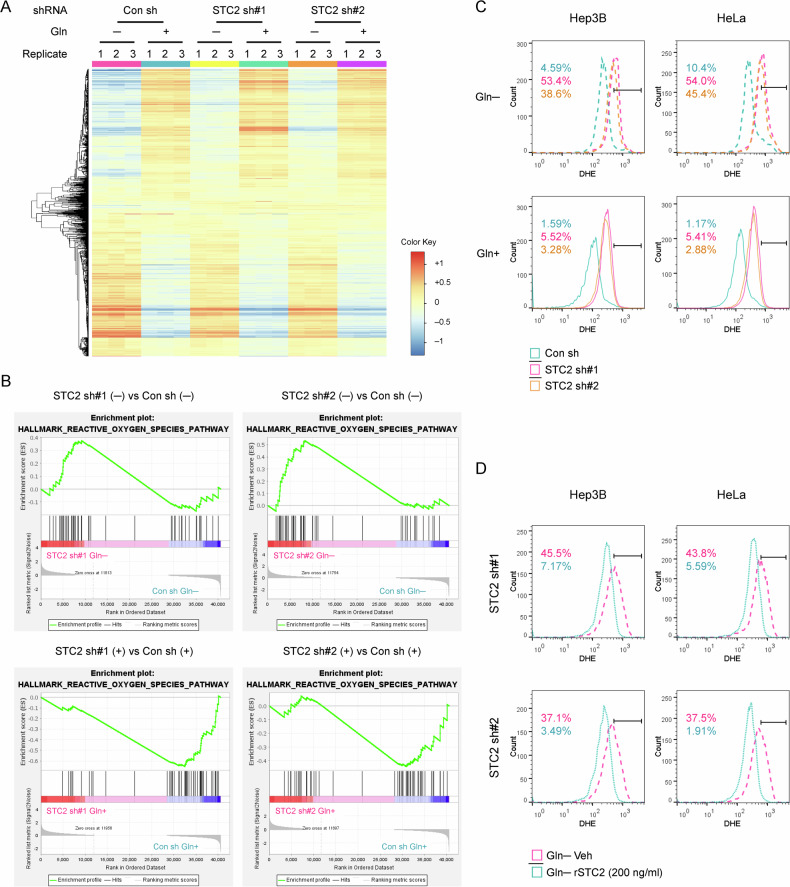
STC2 knockdown alters the expression signature of genes participating in redox homoeostasis and increases ROS levels in cells cultured in Gln-deprived media. **A** iDEP analysis shows the RNA-Seq data in STC2 knockdown versus control cells. This analysis was performed to identify the altered signalling pathways in STC2 knockdown cells in Gln-free media. **B** GSEA analysis highlights the enrichment of genes related to redox homoeostasis in STC2 knockdown cells exposed to Gln-free media. **C** After dihydroethidium (DHE) staining, flow cytometry analyses were performed, and the results indicate that Gln-deprivation increases ROS levels in both Hep3B and HeLa cells and STC2 knockdown further increases ROS levels. Three biological replicates were performed, one representative result is shown. **D** rSTC2 treatment reduces ROS levels in Hep3B and HeLa cells in Gln-free media. The numbers in (**C**, **D**) indicate the percentage of cells with elevated ROS levels. Three biological replicates were analysed, one representative result is shown.

Next, we asked how STC2 protects tumour cells from nutrient insufficiency-induced apoptosis. To address this question, we first performed RNA-Seq analysis to search for alterations of signalling pathways in STC2 knockdown versus control cells cultured in Gln-free media (Fig. 6A). The analysis of the RNA-Seq data identified an alteration of genes related to “hallmark gene sets” (Supplementary Fig. S10); particularly, both shRNAs targeting STC2 resulted in an elevation of genes related to redox homoeostasis under Gln-deprived conditions (Fig. 6B). We next determined how STC2 status affected ROS levels in cells exposed to Gln-deprivation. Using dihydroethidium (DHE) staining combined with flow cytometry analyses, we observed that Gln-deprivation moderately increased ROS levels, and STC2 knockdown significantly intensified ROS accumulation (Fig. 6C). Moreover, the administration of rSTC2 to culture media obviously decreased ROS levels in STC2 knockdown cells (Fig. 6D). Taken together, these data indicate that STC2 induction and secretion are required to ameliorate ROS elevation triggered by nutrient insufficiency.

### STC2 knockdown leads to MAOB dysregulation and redox imbalance during nutrient insufficiency

**Fig. 7:**
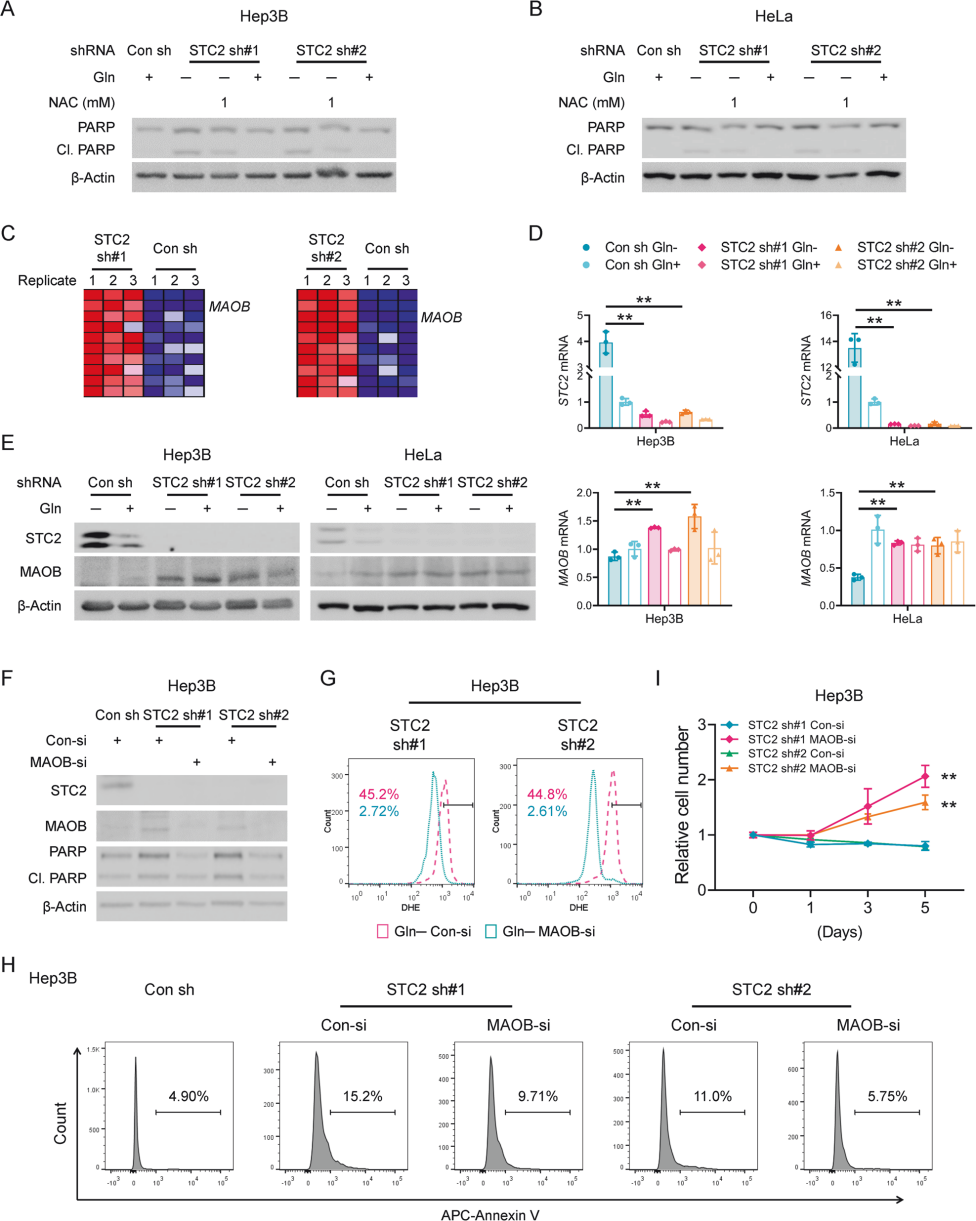
Loss of STC2 leads to MAOB dysregulation and redox imbalance when exposed to Gln-free media. **A**, **B** ROS scavenger NAC decreases PARP cleavage in Hep3B (**A**) and HeLa (**B**) cells in Gln-free media. **C** Blue-Pink O’ Gram in the Space of the analysed Gene Set, the comparison of redox metabolic genes between STC2 knockdown and control cells cultured in Gln-free media. *MAOB* is one of the genes most significantly upregulated in STC2 knockdown cells. Detailed genes are listed in Supplementary Table 3. **D** qRT-PCR validates the upregulation of *MAOB* mRNA in STC2 knockdown Hep3B and HeLa cells cultured in Gln-free media. Data are shown as the mean ± SD; **P < 0.01; *n* = 3. **E** Western blot confirms that MAOB levels are higher in STC2 knockdown Hep3B and HeLa cells cultured in Gln-free media. **F** MAOB knockdown reduces PARP cleavage in STC2 knockdown Hep3B cells cultured in Gln-free media. **G** MAOB knockdown decreases ROS levels in STC2 knockdown Hep3B cells in Gln-free media. The numbers indicate the percentage of cells with elevated ROS levels. **H** MAOB knockdown prevents Gln-deprivation-triggered apoptosis of STC2 knockdown Hep3B cells. **I** MAOB knockdown rescues the proliferation of STC2 knockdown Hep3B cells cultured in Gln-free media. Data are shown as the mean ± SD; **P < 0.01; *n* = 4.

To confirm a causative relationship between ROS accumulation and apoptosis in our experimental model, we administered NAC or GSH to STC2 knockdown cells. Not surprisingly, both NAC and GSH suppressed apoptosis of STC2 knockdown cells enduring Gln-deprivation (Fig. 7A, B and Supplementary Figs. S7 and S11), indicating increased ROS is a bona fide mediator of apoptosis in our experimental setting. To explore the molecular mechanism underlying the protective effects of upregulated STC2 in response to nutrient insufficiency, we mined the RNA-Seq data and found that *MAOB*, a mitochondrial membrane-associated enzyme that directly participates in ROS production, was among the genes most upregulated by STC2 knockdown (Fig. 7C and Supplementary Table 3). Subsequent qRT-PCR and western blot assays further confirmed that STC2 knockdown increased *MAOB* expression under Gln-deprived conditions (Fig. 7D, E), whereas no significant alteration of expression levels of other genes related to ROS generation was found in two independent STC2 knockdown cell lines. Particularly, no signal was detected for NADPH oxidase (NOX) family genes in both STC2 knockdown cell lines (Supplementary Fig. S12). In accordance, we did not find a significant change in mitochondrial ROS in STC2 knockdown cells (Supplementary Fig. S13).

To further explore the regulatory relationship between STC2 and MAOB, we applied rSTC2 to cells with STC2 knockdown. We observed that rSTC2 effectively decreased MAOB expression in STC2 knockdown cells in Gln-free media (Supplementary Fig. S14). To substantiate the biological importance of MAOB expression to ROS levels and cell survival, we used siRNA pool to knockdown MAOB, or specific inhibitors to suppress MAOB activity in STC2 knockdown cells. We observed that MAOB knockdown efficiently decreased ROS levels in STC2 knockdown cells cultured in Gln-free media (Fig. 7F, G and Supplementary Fig. S15A, B). In addition, MAOB knockdown not only suppressed apoptosis but also rescued cell proliferation (Fig. 7H, I and Supplementary Fig. S15C, D). To further evaluate whether MAOB is associated with STC2 loss, we employed two specific MAOB inhibitors, Rasagiline and Selegiline. Consistently, both inhibitors effectively attenuated ROS production and apoptosis in STC2 knockdown cells cultured in Gln-free media (Supplementary Fig. S16A–D), thereby rescuing cell proliferation (Supplementary Fig. S16E, F). As a metabolic precursor or substrate, the lack of Gln showed a detrimental effect on GSH levels. MAOB inhibitors, Rasagiline and Selegiline, slightly increased GSH levels, but in some experiments, these changes barely meet the criteria of statistical significance. Similarly, MAOB knockdown did not significantly change GSH levels (unpublished data), indicating MAOB is more likely a fine tuner of the GSH/GSSG ratio, which is reflected by the alteration of intracellular ROS levels and controls the survival or death of cells enduring oxidative stress. Taken together, these findings indicate that under nutrient-insufficient conditions, STC2 induction maintains redox homoeostasis mainly by suppressing MAOB expression, thus reducing intracellular ROS levels and maintaining cell survival.

### The expression levels of STC2 and MAOB are negatively correlated, and elevated STC2 is associated with tumour progression in vivo

**Fig. 8:**
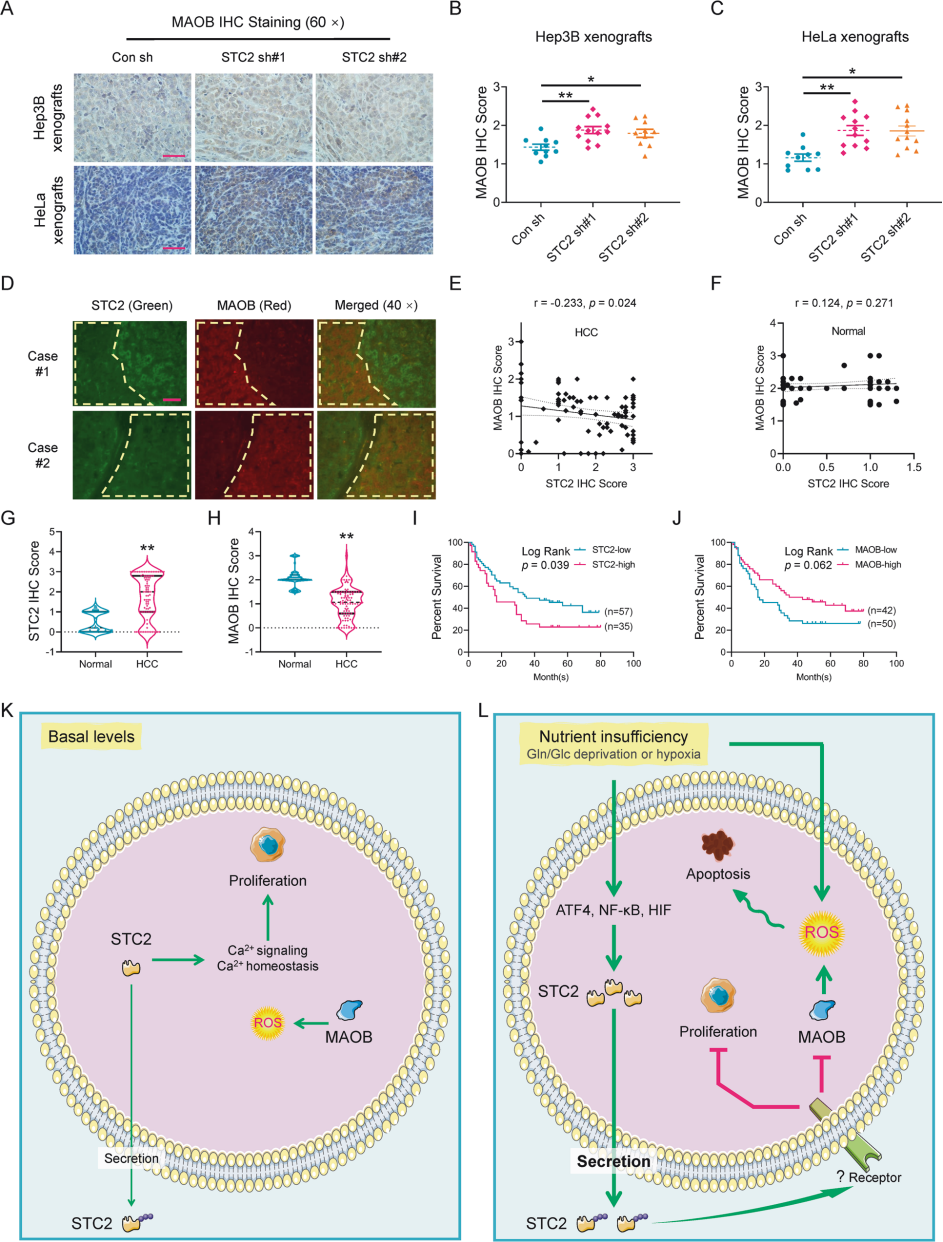
The expression levels of STC2 and MAOB are negatively correlated, and elevated STC2 is associated with tumour progression. **A** Elevated MAOB levels are revealed in STC2 knockdown xenografts by IHC staining. Scale bar, 50 μm. **B**, **C** The xenografts from cells with STC2 knockdown show higher MAOB IHC staining score. All data are shown as the mean ± SD; *P < 0.05; **P < 0.01; *n* = 10–12. **D** The immunofluorescent staining indicates the reverse correlation between STC2 (green) and MAOB (red) expression in HCC specimens. Yellow line encircled area with low STC2 expression but high MAOB levels. Scale bar, 50 μm. **E**, **F** TMA IHC staining reveals a reverse correlation between STC2 and MAOB expression in HCC but not normal samples. **G**, **H** Elevated STC2 (**G**) and decreased MAOB (**H**) levels are detected in human HCC versus normal samples. **P < 0.01. **I**, **J** High STC2 expression is significantly correlated with poor prognosis in patients with HCC. **K**, **L** Schematic illustration of the proposed role of STC2 signalling in tumour cell adaptation to nutrient insufficiency. Under normal conditions, basal levels of STC2 perform its biological functions mainly by affecting intracellular targets, for example, maintaining Ca^2+^ homoeostasis to facilitate cell proliferation. Under nutrient-insufficient conditions, stress-triggered ATF4 and NF-κB activation upregulates STC2 expression. STC2 is secreted out of the cells to function in an autocrine/paracrine manner to prevent apoptosis through the alleviation of oxidative stress and to conserve nutrients by attenuating cell proliferation.

To address whether the observation in cell culture is relevant to tumour progression in vivo, we first performed IHC staining and confirmed the elevated expression of MAOB in STC2 knockdown xenografts (Fig. 8A–C). Furthermore, DepMap analyses suggested a reverse correlation between *STC2* and *MAOB* expression in a series of human tumour cells (Supplementary Fig. S17A). Other bioinformatic analyses also revealed a negative correlation between *STC2* and *MAOB* expression in human tumours (Supplementary Fig. S17B–F).

To investigate the potential of using the reciprocal STC2-MAOB expression as a prognostic indicator, we first performed immunofluorescent staining to examine their expression correlation in human HCC specimens. In consistency with cell culture findings, we confirmed an obviously negative correlation between STC2 and MAOB levels in the same HCC samples (Fig. 8D). IHC staining also verified a reverse correlation between STC2 and MAOB expression in human HCC tissues but not in normal counterparts (Fig. 8E–H and Supplementary Fig. S18). Importantly, clinicopathological analyses revealed that elevated STC2 expression was significantly associated with poor prognosis in patients with HCC, whereas increased MAOB levels were likely correlated with a favourable prognosis with a log-rank *P* value of 0.062 (Fig. 8I, J). Taken together, these data strongly indicate that STC2 negatively regulates MAOB expression via an un-characterised mechanism (Fig. 8K, L), and a combined analysis of both STC2 and MAOB levels is a potential approach in assessing the prognosis of patients with HCC.

## Discussion

The rapid proliferation of tumour cells demands an increased supply of nutrients to support proliferation and active biosynthesis, while a defective blood vessel system often fails to support solid tumour cells with sufficient blood supply, leading to nutrient insufficiency and, subsequently, oxidative stress during tumour progression. Our previous study revealed that *STC2* is one of the most upregulated genes under Gln-/Glc- deprived and hypoxic conditions [1]. With these data in context, our study focuses on addressing how STC2 is upregulated by nutrient insufficiency, and the biological roles of STC2 in the adaptation of tumour cells to nutrient insufficiency.

We presented data to conclude that ATF4 and p65/RelA are activated by nutrient insufficiency that in turn transactivates *STC2* gene. Previous studies reported that STC2 was upregulated upon ATF4 activation [8, 18], but it was unclear whether ATF4 directly transactivates *STC2* gene, or indirectly regulates its expression. Our current study identifies the *cis*-elements of the *STC2* gene and demonstrates that these elements are indispensable for *STC2* induction upon nutrient deprivation. It is well known that eIF2α-ATF4 axis represents the core of an integrated stress response mechanism [34]. Under various stress conditions, cells utilise multiple kinases to phosphorylate eIF2α. Phosphorylation of eIF2α results in a global translation inhibition but a selective translation of stress-related genes exemplified by ATF4. As a stress-responsive transcription factor, ATF4 transcribes several functional groups of genes, including ER chaperones that are involved in protein folding, ER-associated degradation of terminally damaged proteins, and apoptosis. In addition to ATF4, NF-κB is another transcription factor ubiquitously involved in cellular stress response. It was suggested that Gln-deprivation activated NF-κB signalling [35]. Consistent with that report, our findings demonstrate that p65/RelA, a member of NF-κB family, directly transactivates STC2 gene by a physical interaction with identified *cis*-elements upon activation. Considering NF-κB is activated by various cell injury, pathogen infection and inflammatory signalling, we deduce that STC2 could also be induced by a variety of stress, including cell injury and inflammatory factors.

Elevated ROS is a common result of many cellular stress and disease status, and is a major contributor to oxidative stress imbalance. ROS is mainly generated in the mitochondria as a byproduct of oxidative phosphorylation or in the cytosol from reactions catalysed by oxidoreductases such as NOX [36]. To some extent, ROS possesses beneficial effects for tumour cells, driving cell proliferation, genetic instability and metastasis [37]. However, excessive and/or long-term ROS may lead to the damage of macromolecules, and eventually cell death. From the nutrient point of view, Gln and Glc play critical roles in the intracellular counter-oxidative stress processes [25, 26]. Gln is the direct precursor of glutamate; both Gln and glutamate play central roles as a nitrogen donor in the biosynthesis of non-essential amino acids, including cysteine and glycine. Glutamate, cysteine and glycine are the direct substrates for GSH biosynthesis. Indeed, we observed that Gln-/Glc- deprivation altered the amino acid metabolic pathway (Supplementary Fig. S1B), reflecting a feedback response to global amino acid insufficiency that directly impair the biosynthesis of GSH and possibly other antioxidants. Our data revealed that tumour cells exposed to Gln-free media have elevated ROS levels, and STC2 knockdown further increases ROS levels, suggesting that STC2 plays a role in maintaining redox homoeostasis. RNA-Seq analyses also revealed that the change of ROS metabolic signature is one of the most affected pathways in STC2 knockdown cells upon Gln-deprivation, further providing additional evidence to our hypothesis.

Previous studies indicated a connection between oxidative stress and STC2 induction. For example, H_2_O_2_ treatment leads to STC2 upregulation, and loss of STC2 causes cell death when challenged with oxidative stress [18–20]. We note that a variety of cell stress, including nutrient insufficiency, may trigger oxidative stress and ER stress. Accordingly, as a primary or a secondary alteration, oxidative stress itself may be sufficient to independently activate ATF4 and NF-kB, hence upregulating STC2.

Whereas STC1 has been reported to regulate redox homoeostasis by promoting the expression of uncoupling protein 2, thus compromising the ETC-related ROS production [38, 39], our findings in this study suggest that STC2 utilises a novel mechanism to maintain the cellular redox homoeostasis during nutrient insufficiency, i.e., STC2 induction by nutrient insufficiency suppresses MAOB expression to decrease ROS production. MAOB belongs to the family of monoamine oxidase (MAO) enzymes that localise in the outer mitochondrial membrane to catalyse the oxidation of various monoamines [40]. MAOB is mainly expressed in liver, heart, duodenum, lung and brain [41]. Under physiological conditions, there is a clear increase of MAOB expression in aging or inflammatory brains [42, 43]. The reaction catalysed by MAOB is regarded as a source of H_2_O_2_ that is involved in ROS-mediated cellular injury in neurological and cardiac disorders [44–47]. In consistent with the literature, our data indicate that STC2 suppresses MAOB expression, whereas loss of STC2 leads to MAOB induction, which coincides with elevated ROS levels and cell death upon Gln-deprivation, highlighting the role of MAOB as a redox regulator during nutrient insufficiency.

Besides its functions in catalysing monoamine, it remains unclear whether MAOB directly affects the activity of ETC, the major source of ROS production. A previous study reported that MAOA, another member of the MAO family, is involved in regulating mitochondrial bioenergetics, and the MAOA-specific inhibitor, clorgyline, enhances mitochondrial ROS production at high concentrations by increasing cellular respiration [48].

Importantly, this study found the administration of rSTC2 can efficiently block Gln-deprivation-induced apoptosis, suggesting STC2 may perform its biological functions in an autocrine/paracrine manner. Till now, the majority of investigations have focused on utilising the genetically overexpressing systems to investigate the biological functions of STC2 to establish its role in regulating cell proliferation, migration, invasion and apoptosis [8]. But as a secreted glycoprotein, overexpression of STC2 leads to increased secretion when it has its own signal peptide (our unpublished data). Therefore, it’s unreasonable to simply attribute the biological effects of STC2 overexpression to its intracellular functions [49]. Our study found rSTC2 efficiently blocks MAOB induction, resulting in reduced ROS production and apoptosis under Gln-deprived conditions. These findings strongly suggest STC2 works in an autocrine/paracrine manner to regulate cellular processes. A recent study found the administration of rSTC2 promotes epithelial-mesenchymal transition by inducing the expression of snail family transcription repressor 2 and matrix metalloproteinases [16]. Recently, several clinicopathological studies have identified serum STC2 levels are elevated in patients with HCC, gastric cancer and colorectal cancer, and particularly high serum STC2 levels are associated with poor prognosis [20, 50–52], indicating secreted STC2 could perform its biological functions through an endocrine mechanism at organism levels. It has been reported that the cation-independent mannose-6-phosphate receptor/IGF2R interacts with STC1 and may serve as a receptor [53]. Whether STC2 shares the same receptor or uses a different surface protein as a receptor remains to be investigated.

Although a plethora of clinicopathological investigations indicate that STC2 expression is correlated with tumour size, disease stage and prognosis of various human tumours, that is also demonstrated by the bioinformatic analyses of the TCGA data in our study [8], it remains unclear how STC2 induction could biologically benefit tumour growth. This study provides new insights into our understanding of the role of STC2: serving as a critical regulator to coordinate cell survival and proliferation under nutrient-insufficient conditions. Specifically, STC2 is required to support optimal cell proliferation under normal conditions, indicating basal levels of intracellular STC2 facilitate cell proliferation, which might be attributed to its function in maintaining intracellular calcium homoeostasis (Fig. 8K, L). Under nutrient-insufficient conditions, STC2 slows down cell proliferation which might be more beneficial for tumour cells by allocating limited resources to fulfil their basic demands, such as maintaining cell survival. A potential explanation might be that various types of cellular stress triggers STC2 induction and secretion into the culture media; and the secreted STC2 serves as a ligand to activate downstream signalling, which subsequently slows down cell proliferation (Fig. 8K, L). Therefore, STC2 serves as a switch of cell priority: under normal conditions, basal levels of STC2 facilitate maintaining normal cell biology and functions; under stress conditions, STC2 is upregulated and secreted, thereby triggering a stress response that shifts cellular priority to survival.

It is an interesting question regarding the roles of STC2 in non-cancer cells. After careful reviewing of available literature and our own data, we propose that STC2 functions as a critical regulator of cellular adaptation to intracellular/extracellular metabolic state. Under normal conditions, the basal level of STC2 is likely to be an ER resident protein that supports specialised normal cell function via regulating calcium homoeostasis and dynamics. Under various stress conditions, transcriptionally induced STC2 is predominantly secreted as a signalling molecule to suppress non-survival related cellular functions to save resources, thus shifting the cellular priority to survival. This should confer the survival advantage of normal cells during microenvironmental challenges, but in cancers, it may facilitate therapeutical resistance by slowing down proliferation and promoting cell survival. Detailed physiological functions and signalling pathways of STC2 deserve further investigation.

In summary, as a secreted glycoprotein, STC2 is quickly and remarkably transactivated by ATF4 and p65/RelA in cells enduring nutrient insufficiency. As a signalling molecule, secreted STC2 functions as a ligand in an autocrine/paracrine manner to promote cell survival by alleviating oxidative stress. One possible mechanism is that STC2 suppresses MAOB expression at the transcriptional level, thus decreasing ROS production. Although the receptor of STC2 and its downstream signalling remain to be dissected, our current findings support a model that STC2 switches cancer cells’ priority from proliferation to survival, hence facilitating cellular adaptation to nutrient insufficiency. Therefore, STC2 can be regarded as a prognostic biomarker and a promising therapeutic target for cancer treatment.

### Supplementary information

STC2 Supplementary Figures and Tables

STC2 Supplementary Materials and Methods

Original Data

## Data Availability

All datasets generated are included in the main text and its Supplementary Information files. Additional data are available from SQ (shuoqie@tmu.edu.cn) upon reasonable request.
