# Supplementary material for: Stanniocalcin 2 governs cancer cell adaptation to nutrient insufficiency through alleviation of oxidative stress
Source: Cell Death Dis. 2024 Aug 6;15(8):567. doi: 10.1038/s41419-024-06961-7 (PMC11303387; doi:10.1038/s41419-024-06961-7)
Supplement: Supplementary file 1 — STC2 Supplementary Figures and Tables [file 41419_2024_6961_MOESM1_ESM.docx]

**Supplementary Figures and Tables**

**Stanniocalcin 2 governs cancer cell adaptation to nutrient insufficiency through alleviation of oxidative stress**

Shuo Qie^1,2,3,4,#,*^, Haijuan Xiong^1,2,3,4,^**^#^**, Yaqi Liu^1,2,3,4,^**^#^**, Chenhui Yan^1,2,3,4^, Yalei Wang^1,2,3,4^, Lifeng Tian^5^, Chenguang Wang^5^ and Nianli Sang^6,*^

1 Department of Pathology, Tianjin Medical University Cancer Institute and Hospital, Tianjin, 300060 China

2 National Clinical Research Center for Cancer, Tianjin, 300060 China

3 Key Laboratory of Cancer Prevention and Therapy (Tianjin), Tianjin, 300060 China

4 Tianjin's Clinical Research Center for Cancer, Tianjin, 300060 China

5 Department of Cancer Biology, Kimmel Cancer Center, Thomas Jefferson University, Philadelphia, PA, 19107 USA

6 Department of Biology, Drexel University, Philadelphia, PA, 19104 USA

**^#^**, These authors contributed equally to this work.

^*,^ Corresponding authors:

Shuo Qie: Department of Pathology, Tianjin Medical University Cancer Institute and Hospital, Huanhuxi Road, Tiyuanbei, Hexi District, Tianjin 300060, China. Email: shuoqie@tmu.edu.cn

Nianli Sang: Department of Biology, Drexel University, Papadakis Integrated Sciences Building, Room 417, 3245 Chestnut St, Philadelphia, PA 19104, USA. Email: nianli.sang@drexel.edu

**
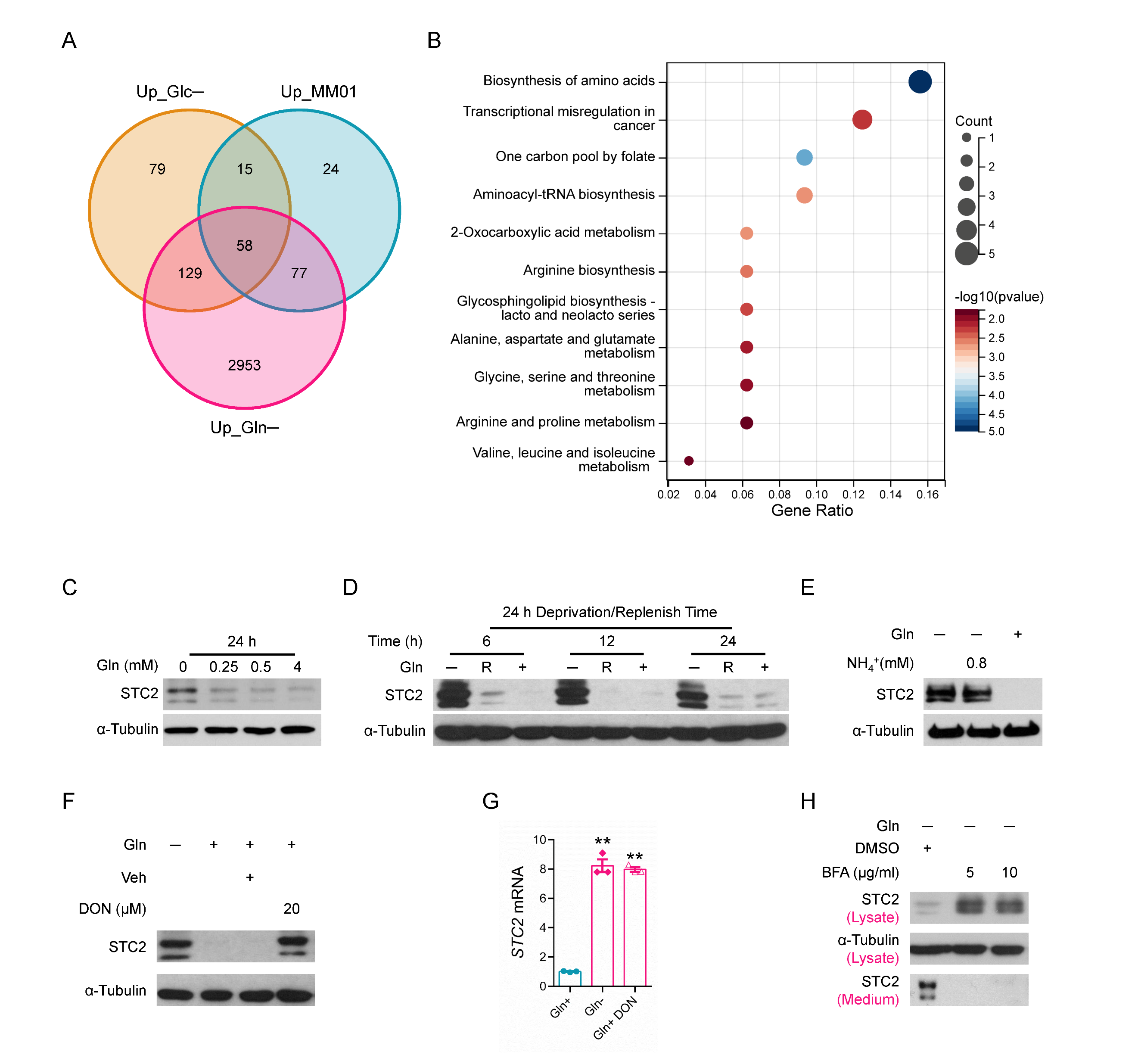
**

**Figure S1.** Either Gln- or Glc- deprivation triggers STC2 induction and secretion. **A** Venn diagram shows numbers of upregulated genes under nutrient deficient conditions. *STC2* is among the 58 genes upregulated by the specified nutrient deficient conditions. **B** Bubble chart shows most of the 58 genes are involved in amino acid metabolism based on KEGG analysis. **C** STC2 levels are reversely correlated with Gln concentrations in Hep3B cells. **D** Re-supplementation of Gln effectively reverses STC2 induction in Hep3B cells. The samples were taken after 24 h culture with (+) or without (─) Gln and analyzed by Western blot. “R” indicates cells that were cultured in Gln-free media for 24 h and re-supplemented with 4 mM Gln for periods as indicated. **E** Supplementation of Gln effectively reduces STC2 expression in MM01 cells. **F, G** Inhibiting Gln catabolism leads to STC2 upregulation at both protein and mRNA levels in Hep3B cells. Data are shown as mean ± SD; **, *p*<0.01; n=3. **H** Gln-deprivation induces secretion of STC2 into culture media which is suppressed by BFA, an inhibitor of protein secretion.

**
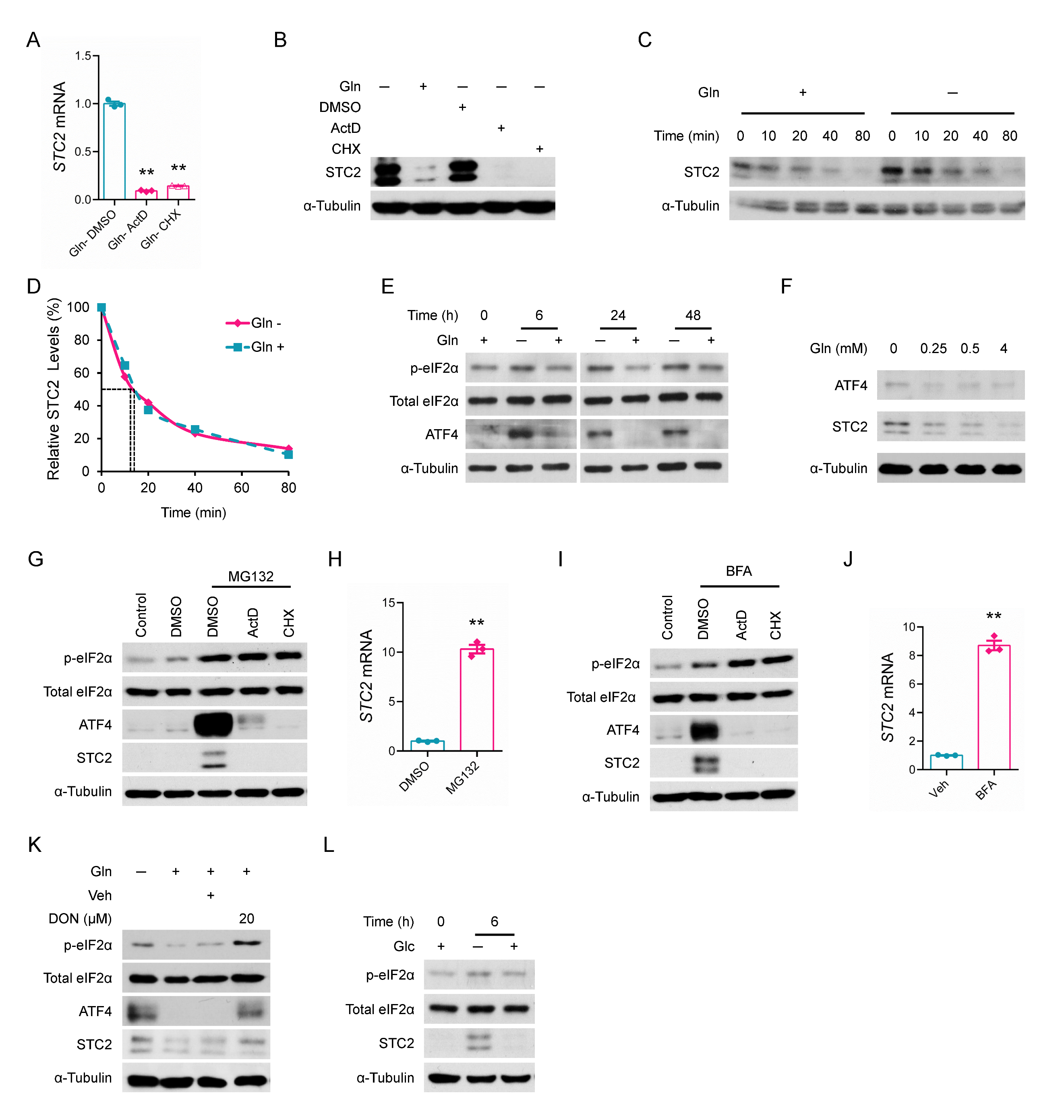
**

**Figure S2.** STC2 upregulation by Gln-deprivation depends on *de novo* transcription and translation. **A, B** ActD or CHX suppresses the expression of STC2 at mRNA (A) or protein (B) levels under Gln-deprived conditions, indicating STC2 transcription depends on translation of a transcription factor. **C** Gln doesn’t affect STC2 protein stability. The half-life of STC2 protein was determined by CHX-chase assay. Hep3B cells were first cultured in Gln-free media for 24 h, and then exposed to media with the translation inhibitor CHX in the presence or absence of Gln. The whole cell lysate at different time points were collected and STC2 levels were examined by western blot. **D** Quantification of STC2 blot bands in “Panel C”, showing lack of Gln made no difference in STC2 decay rates. **E** The integrated stress response eIF2α-ATF4 axis is activated by Gln-deprivation. **F** ATF4 expression is positively correlated with STC2 levels in cells exposed to different concentrations of Gln. **G, H** MG132 increases ATF4 and STC2 expression, but ActD or CHX blocks ATF4 and STC2 accumulation by MG132. **I, J** ER stress inducer BFA activates ATF4 and enhances STC2 expression that is blocked by ActD or CHX. **K** Inhibiting glutaminolysis is sufficient to activate the integrated cell stress response eIF2α-ATF4 axis, leading to STC2 upregulation. **L** Similarly, Glc-deprivation activates eIF2α-ATF4 axis and induces STC2 expression. All data are shown as the mean ± SD; **, *p*<0.01; n=3.


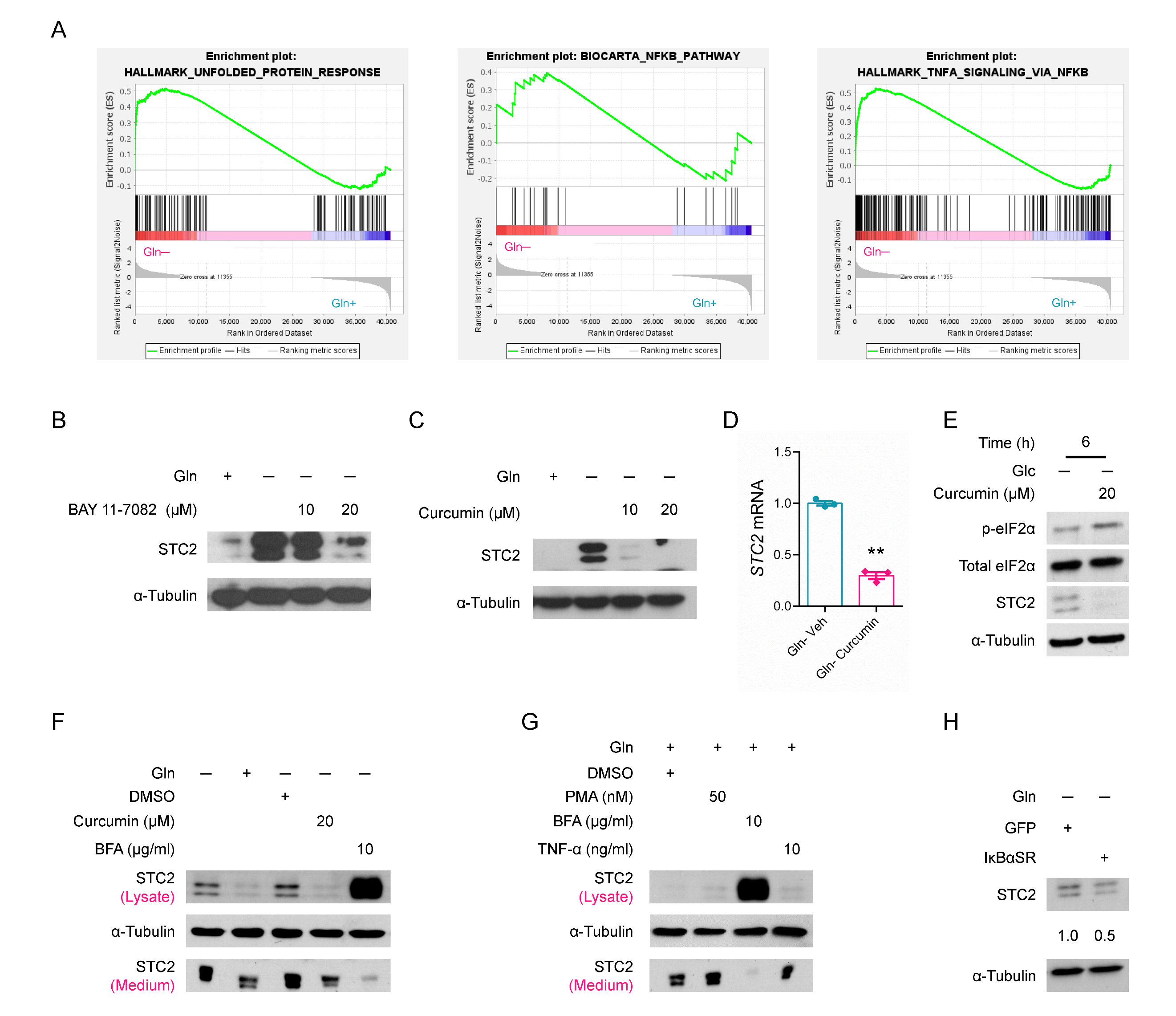


**Figure S3.** NF-κB is involved in STC2 induction by nutrient insufficiency. **A** GSEA analysis indicates the activation of unfolded protein response, NF-κB pathway/TNF-α signaling in cells under Gln-deprived conditions. **B-D** NF-κB inhibitors BAY 11-7082 or curcumin suppresses STC2 induction by Gln-deprivation. Data are shown as the mean ± SD; **, *p*<0.01; n=3. **E** Curcumin suppresses STC2 induction by Glc-deprivation. **F** Curcumin doesn’t enhance STC2 secretion in the absence of Gln. **G** PMA or TNF-α doesn’t suppress STC2 secretion in regular culture conditions. **H** Overexpression of IκBαSR attenuates STC2 induction by Gln-deprivation. The numbers below the blot indicate the quantification of STC2 bands above.


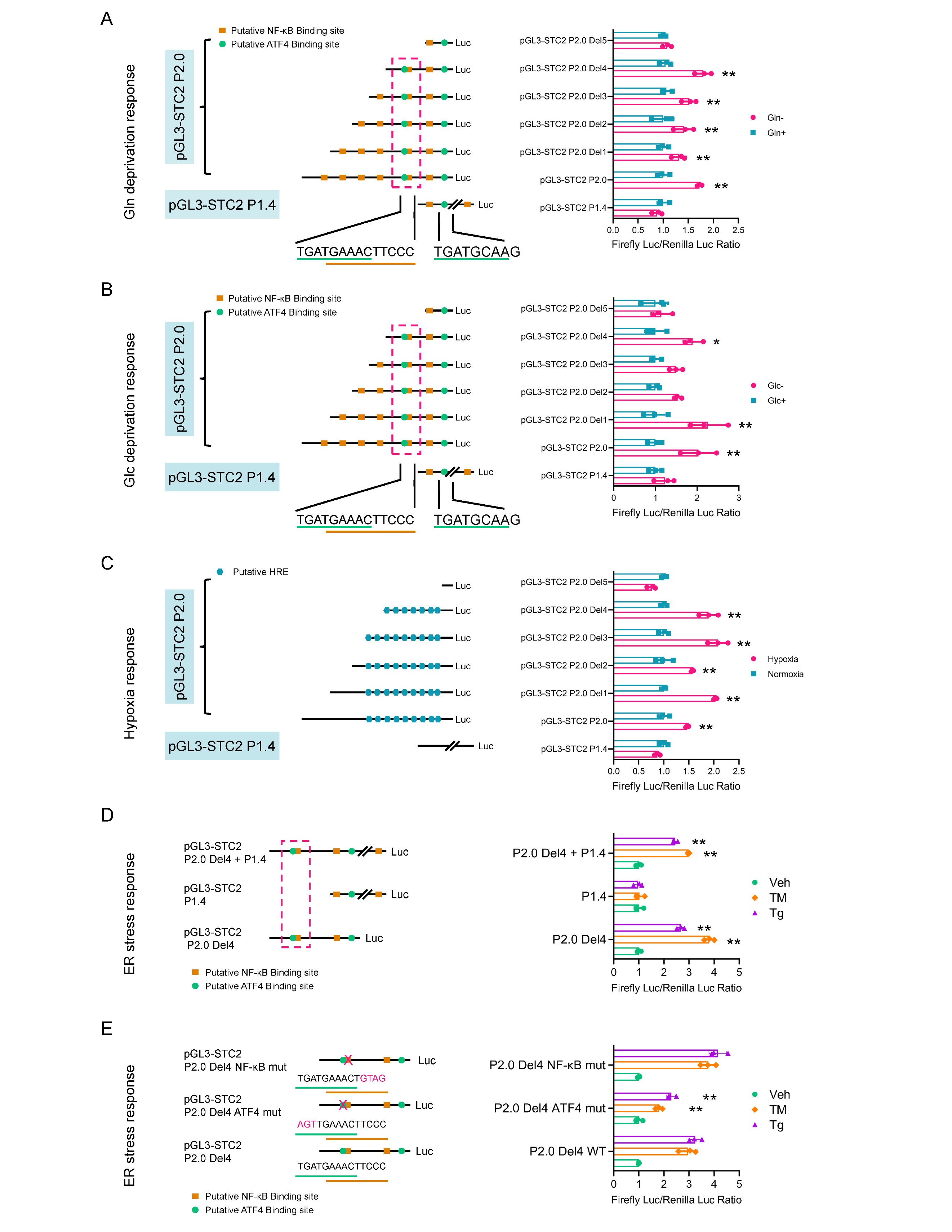


**Figure S4.** Identification of ATF4, NF-κB and HIF-1 binding sites on *STC2* promoter. **A, B** Luciferase assays confirm the *cis* elements required for *STC2* induction by Gln- or Glc- deprivation (highlighted by red rectangle with dash lines). **C** Luciferase assays confirm that the HRE elements on *STC2* promoter are required for hypoxic induction. **D** Luciferase assays identify ATF4 binding sites responsible for *STC2* induction by ER stress inducers, TM or Tg (highlighted by red rectangle with dash lines). **E** Mutation of ATF4 binding site impairs *STC2* induction by TM or Tg. All data are shown as mean ± SD; *, *p*<0.05; **, *p*<0.01; n=3.


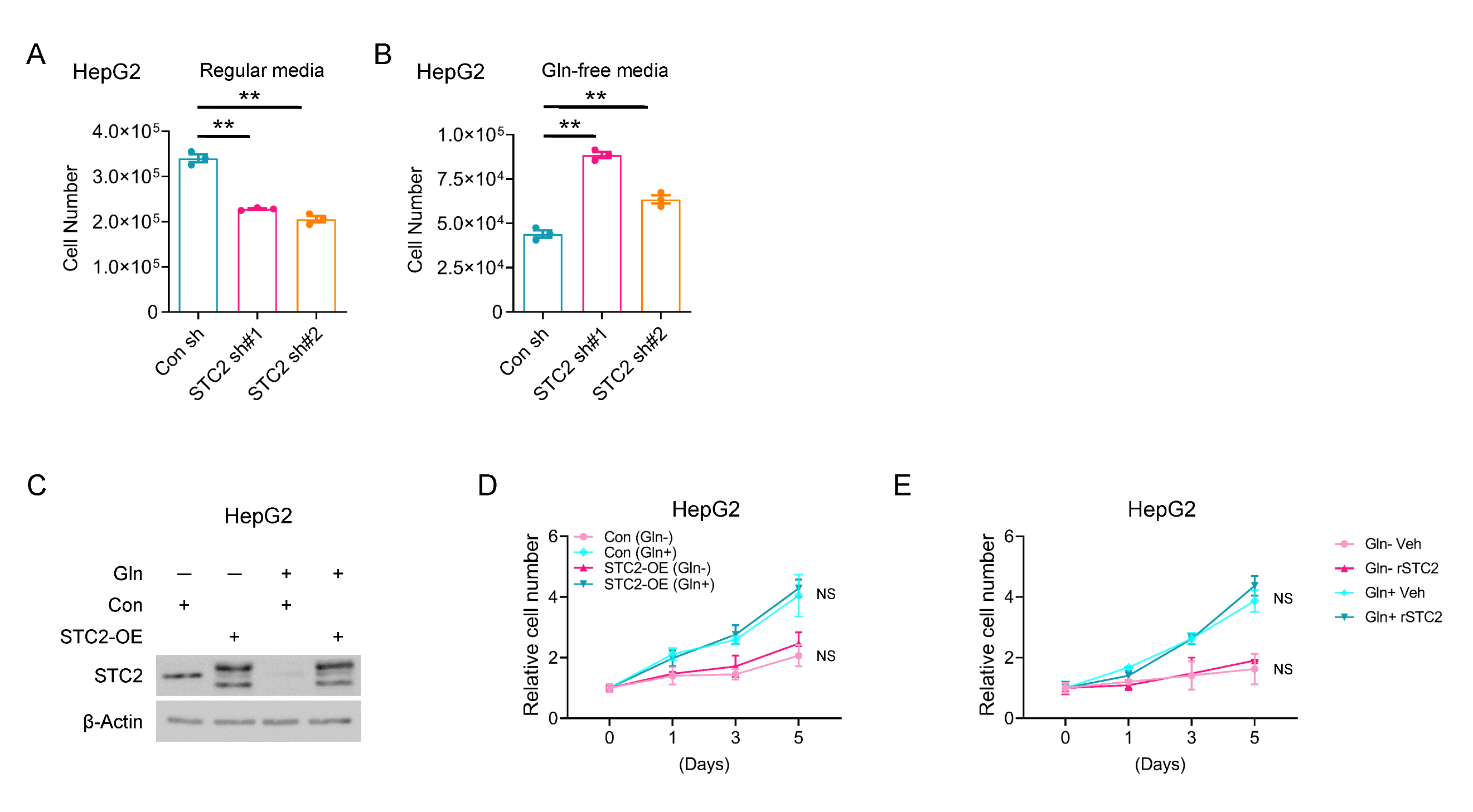


**Figure S5.** STC2 is required for optimal cell proliferation in normal culture media, but negatively regulates cell proliferation upon Gln-deprivation. **A, B** STC2 is needed for optimal proliferation of HepG2 cells in regular media (A) but suppresses cell proliferation in Gln-free media (B). **C, D** Overexpression of STC2 has no apparent effects on the proliferation of HepG2 cells. **E** Exogenous rSTC2 doesn’t affect the proliferation of HepG2 cells, indicating endogenous STC2 is sufficient for its function. All data are shown as the mean ± SD; **, *p*<0.01; NS, not significant, n=3-4.


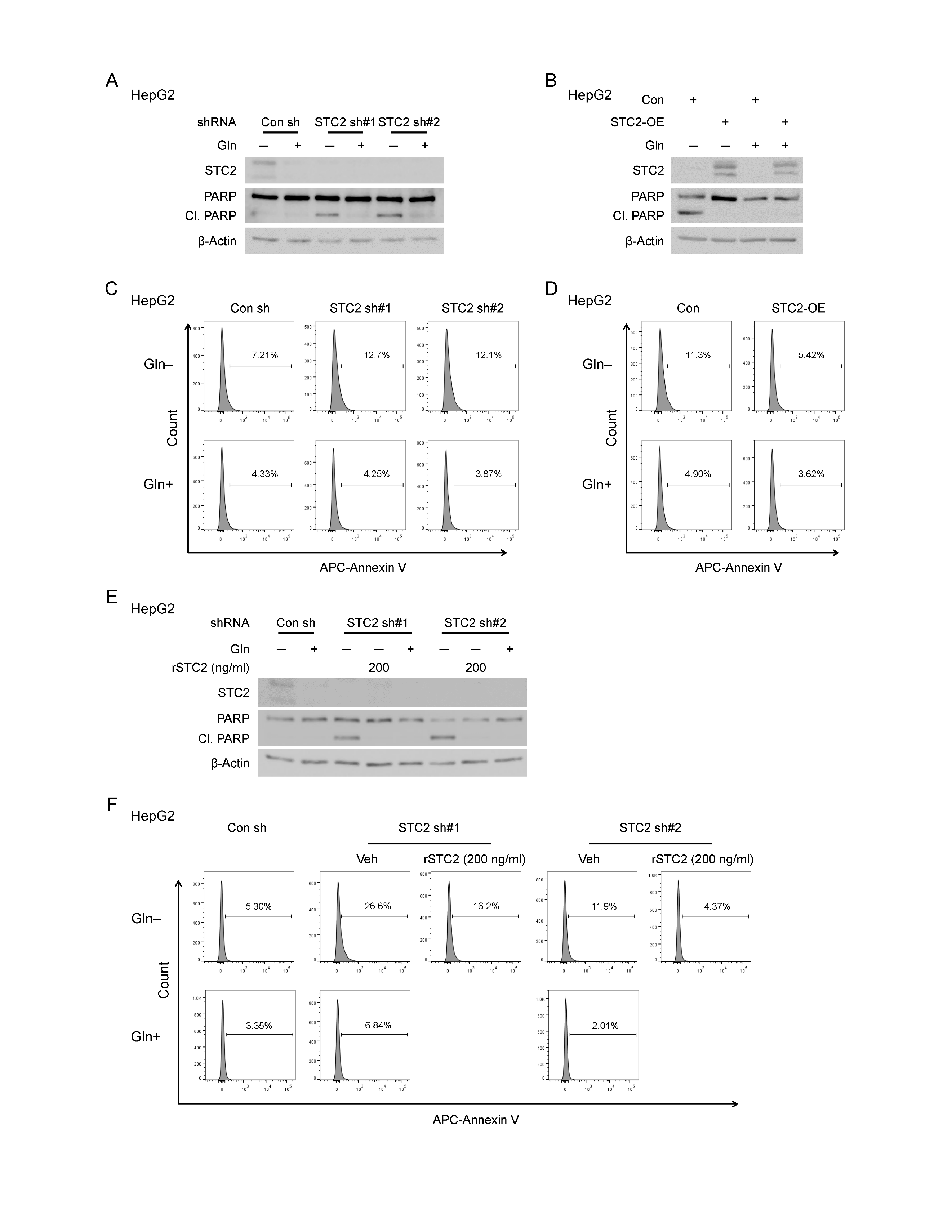


**Figure S6.** STC2 suppresses apoptosis of HepG2 cells under Gln-deprived conditions. **A** STC2 knockdown leads to increased PARP cleavage in HepG2 cells in Gln-deprived media. **B** Overexpression of STC2 suppresses PARP cleavage in HepG2 cells cultured with Gln-free media. **C** Flow cytometry analysis shows increased apoptosis of HepG2 cells as assayed by Annexin V staining. **D** Flow cytometry analysis indicates overexpression of STC2 inhibits apoptosis of HepG2 cells cultured in Gln-deprived media as measured by Annexin V staining. **E, F** rSTC2 effectively decreases STC2 knockdown-triggered PARP cleavage and apoptosis in HepG2 cells cultured in Gln-free media.

**
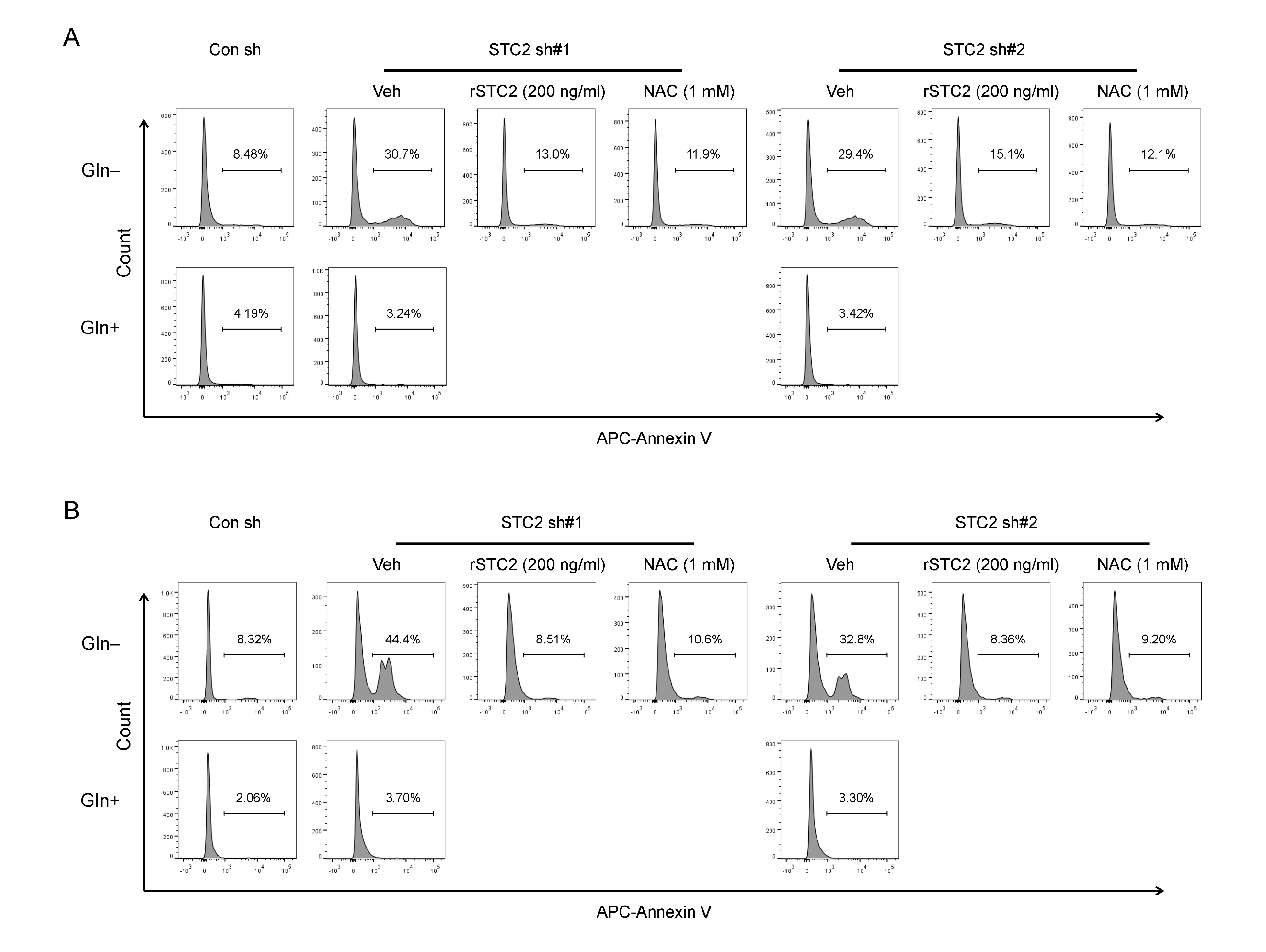
**

**Figure S7.** Administration of rSTC2 or ROS scavenger NAC in culture media suppresses apoptosis triggered by Gln-deprivation. **A, B** Flow cytometry analyses show that the addition of rSTC2 or NAC to culture media decreases apoptosis of STC2 knockdown Hep3B (A) and HeLa (B) cells cultured in Gln-free media. Three biological replicates were performed, and one set of representative data is shown.

**
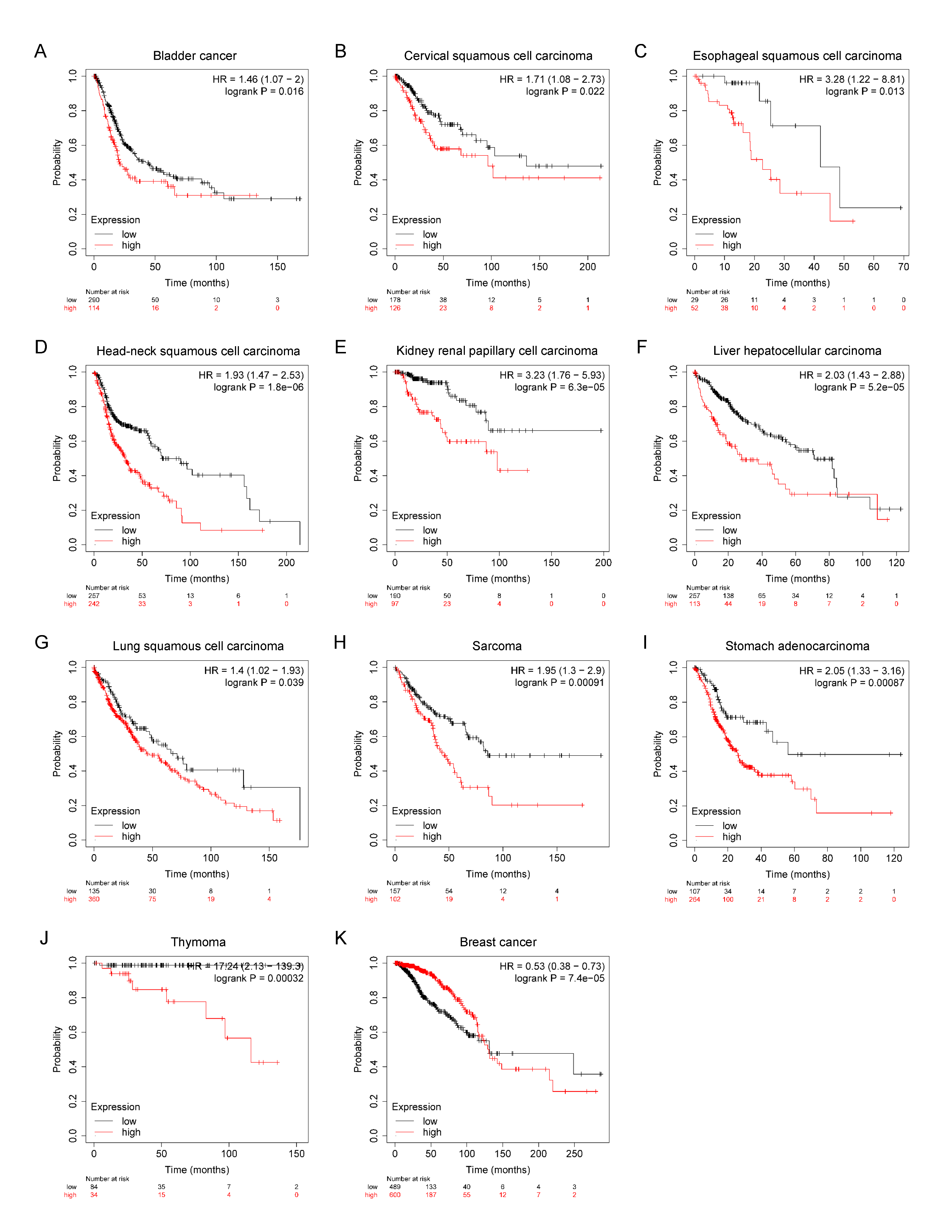
**

**Figure S8.** High STC2 protein levels are associated with poor prognosis of patients with various tumours. **A-K** Pan-Cancer Survival Analysis based on STC2 expression in human tumours with statistical significance using the KM plotter analysis (https://kmplot.com/analysis/).

**
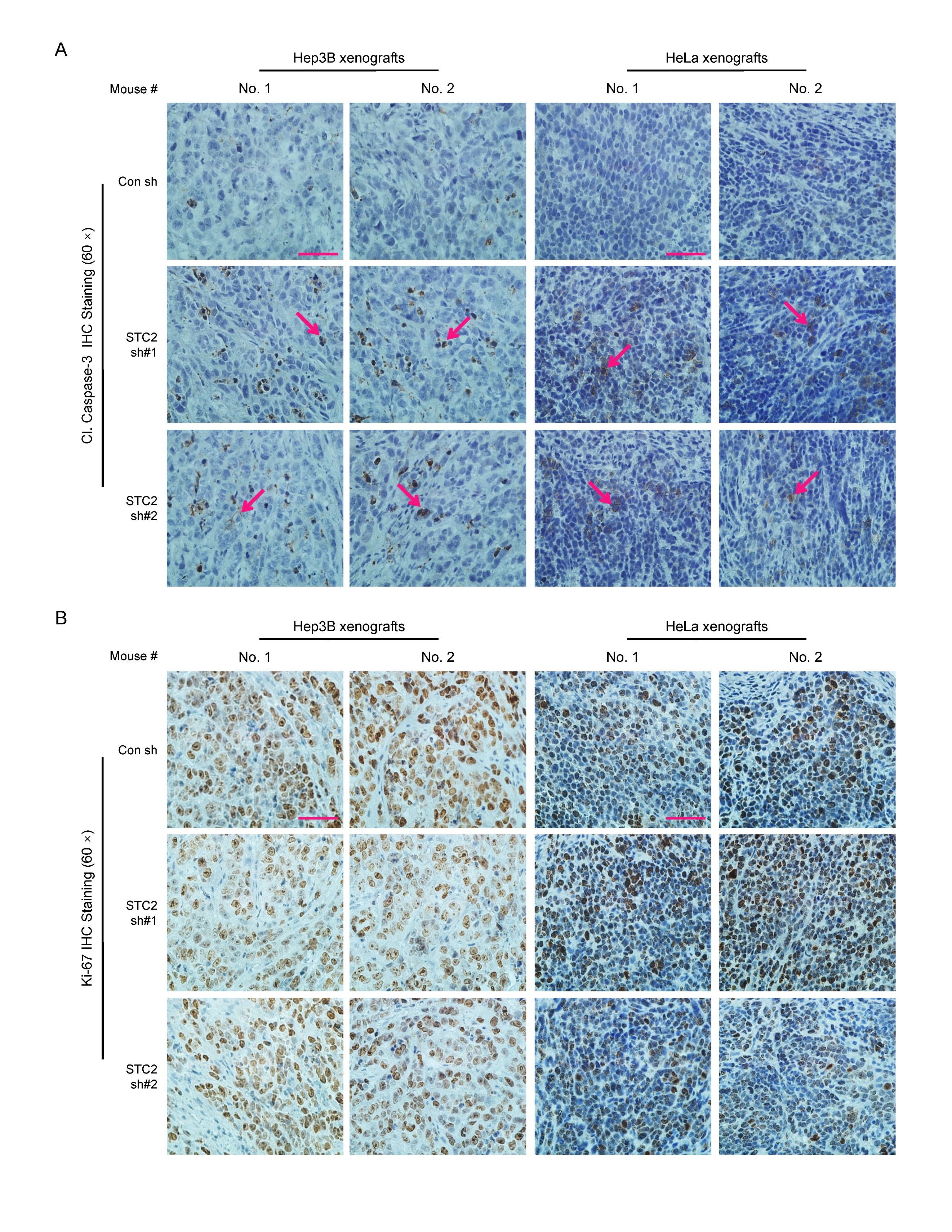
**

**Figure S9.** IHC staining of cleaved caspase-3 and Ki-67 in mouse xenograft specimens. **A** IHC staining shows that cleaved caspase-3 positive (apoptotic) cells increased in STC2 knockdown xenografts. Red arrows indicate cleaved caspase-3-positive cells. **B** IHC staining shows Ki-67 positive (proliferating) cells in specimens from Hep3B and HeLa xenografts. The quantification data of IHC staining and statistical analyses are presented in main text Figure 5. Scale bar, 50 μm.


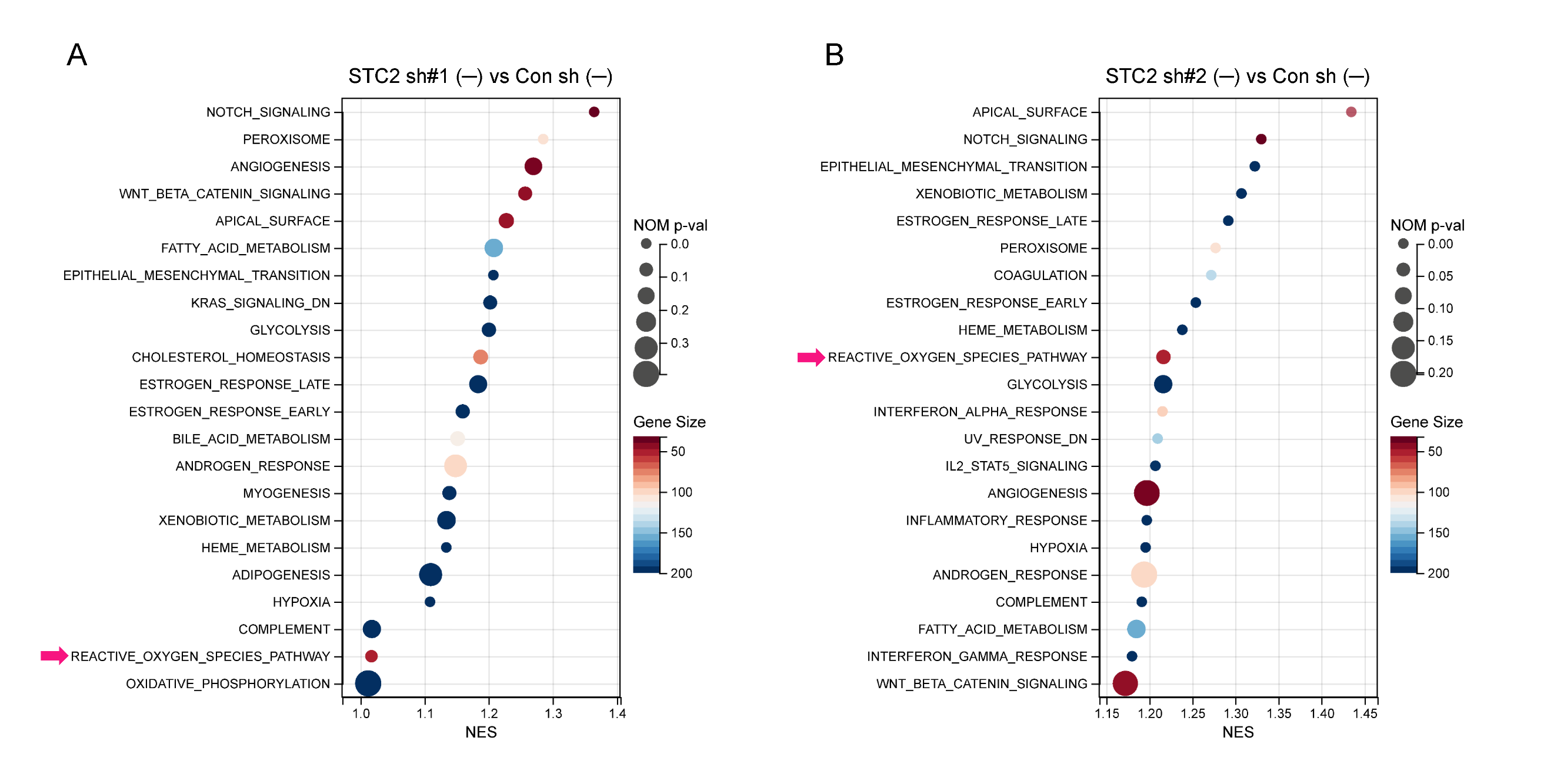


**Figure S10.** Bubble chart shows the activation of Reactive Oxygen Species Pathway (red arrow) based on the KEGG analysis when comparing STC2 knockdown cells with control counterparts in response to Gln-deprivation.


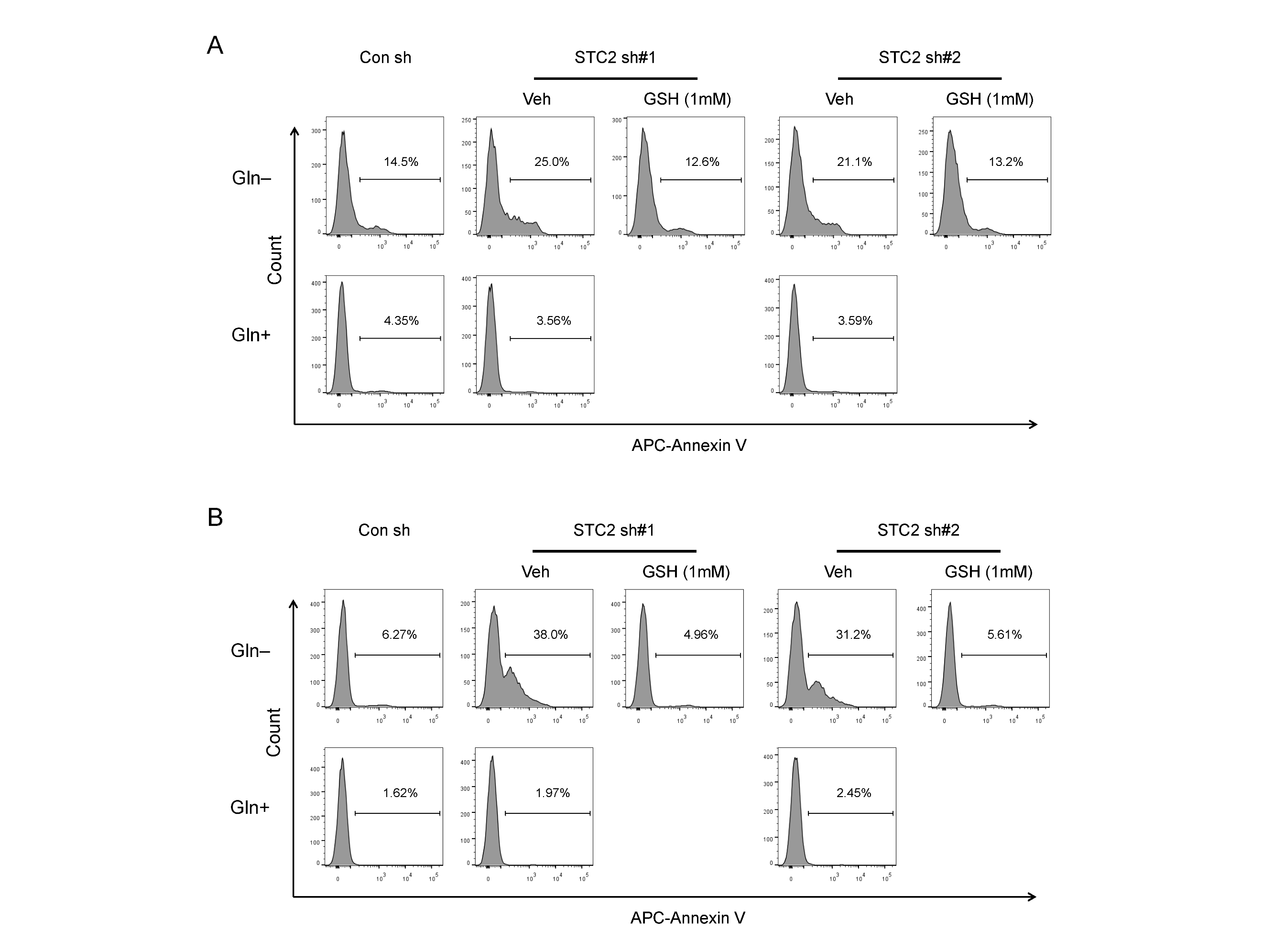


**Figure S11.** Administration of GSH in culture media suppresses apoptosis triggered by Gln-deprivation. **A, B** Flow cytometry analyses show that the addition of GSH to culture media decreases apoptosis of STC2 knockdown Hep3B (A) and HeLa (B) cells cultured in Gln-free media. Three biological replicates were performed, and one set of representative data is shown.

**
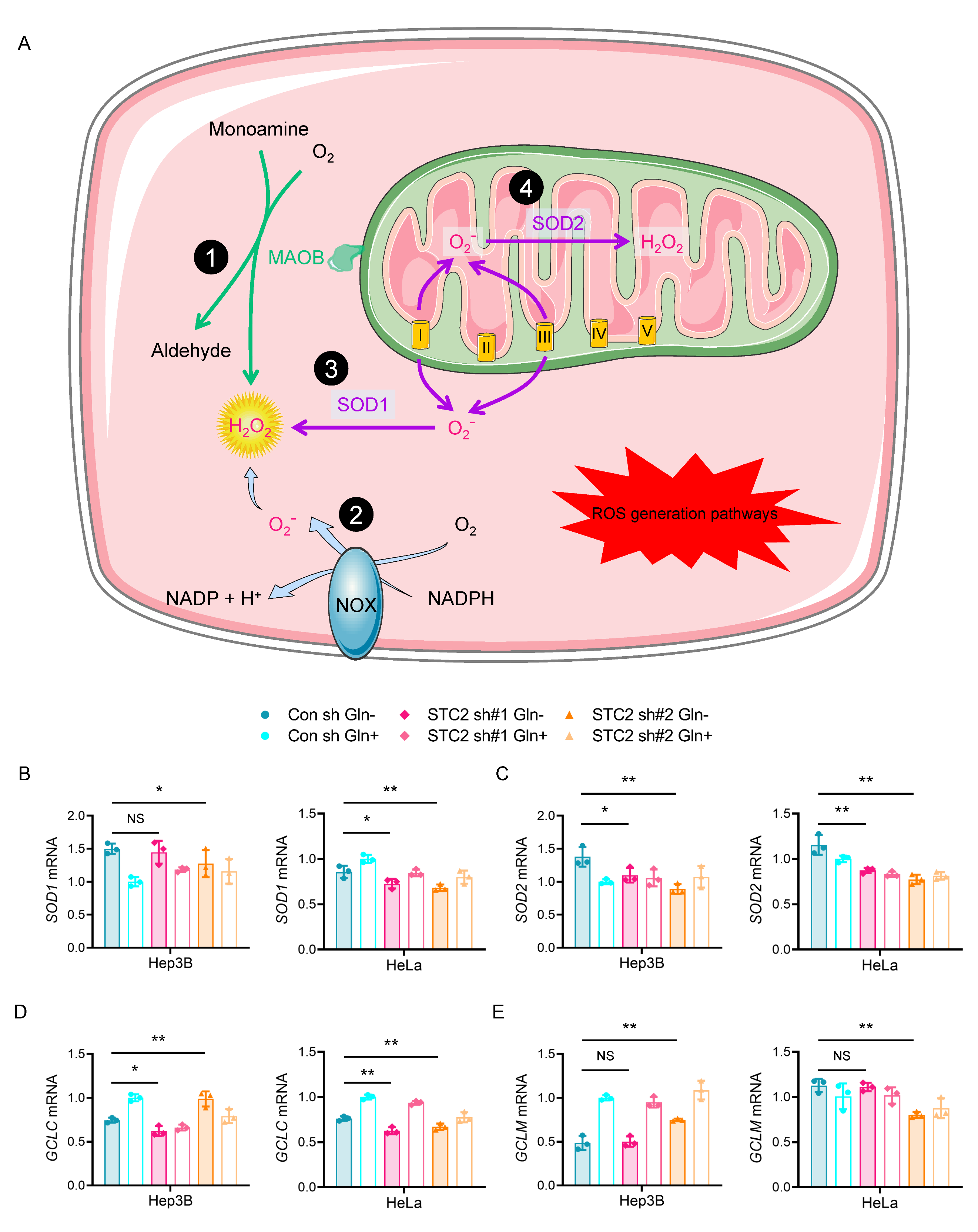
**

**Figure S12.** Effects of STC2 knockdown on mRNA expression of genes involved in ROS metabolism. **A** The schematic illustration of major ROS generating enzymes. 1) Monoamine oxidase B (MAOB) catalyzes the conversion of monoamine to aldehyde, leading to the production of H_2_O_2_. 2) cytosolic NADPH oxidases (NOXs) produce cytosolic ROS by transferring one electron from NADPH to oxygen molecule. 3 & 4) Superoxide dismutases (SOD) convert superoxide radical to H_2_O_2_ and molecular oxygen. **B-E** qRT-PCR analysis of the expression of *SOD1* (B), *SOD2* (C), *GCLC* (D) and *GCLM* (E) genes. Data are shown as mean ± SD; *, *p*<0.05; **, *p*<0.01; NS, not significant, n=3.


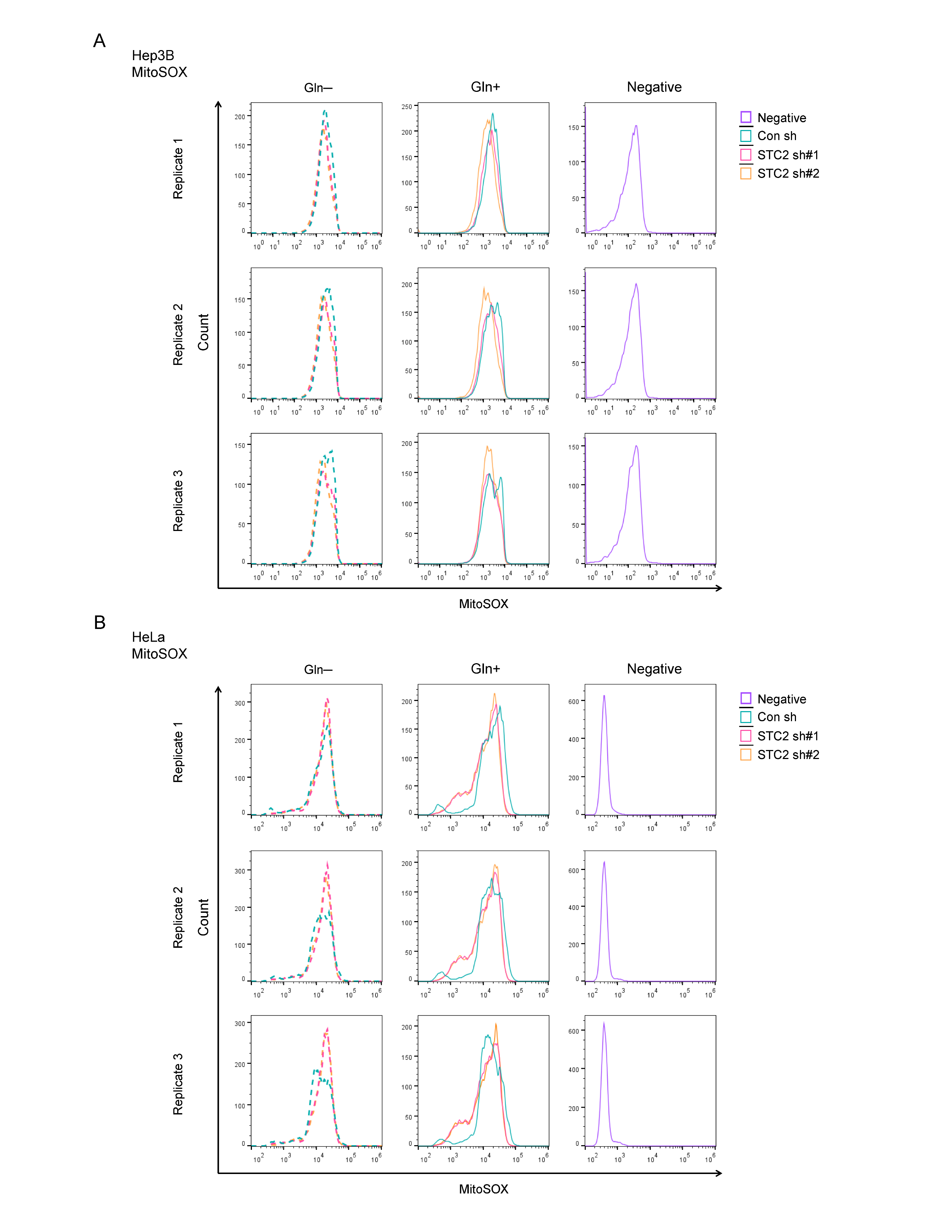


**Figure S13.** MitoSOX staining and flow cytometric analyses. **A, B** Flow cytometry analyses show no significant change of mitochondrial ROS levels in STC2 knockdown Hep3B (A) and HeLa (B) cells.


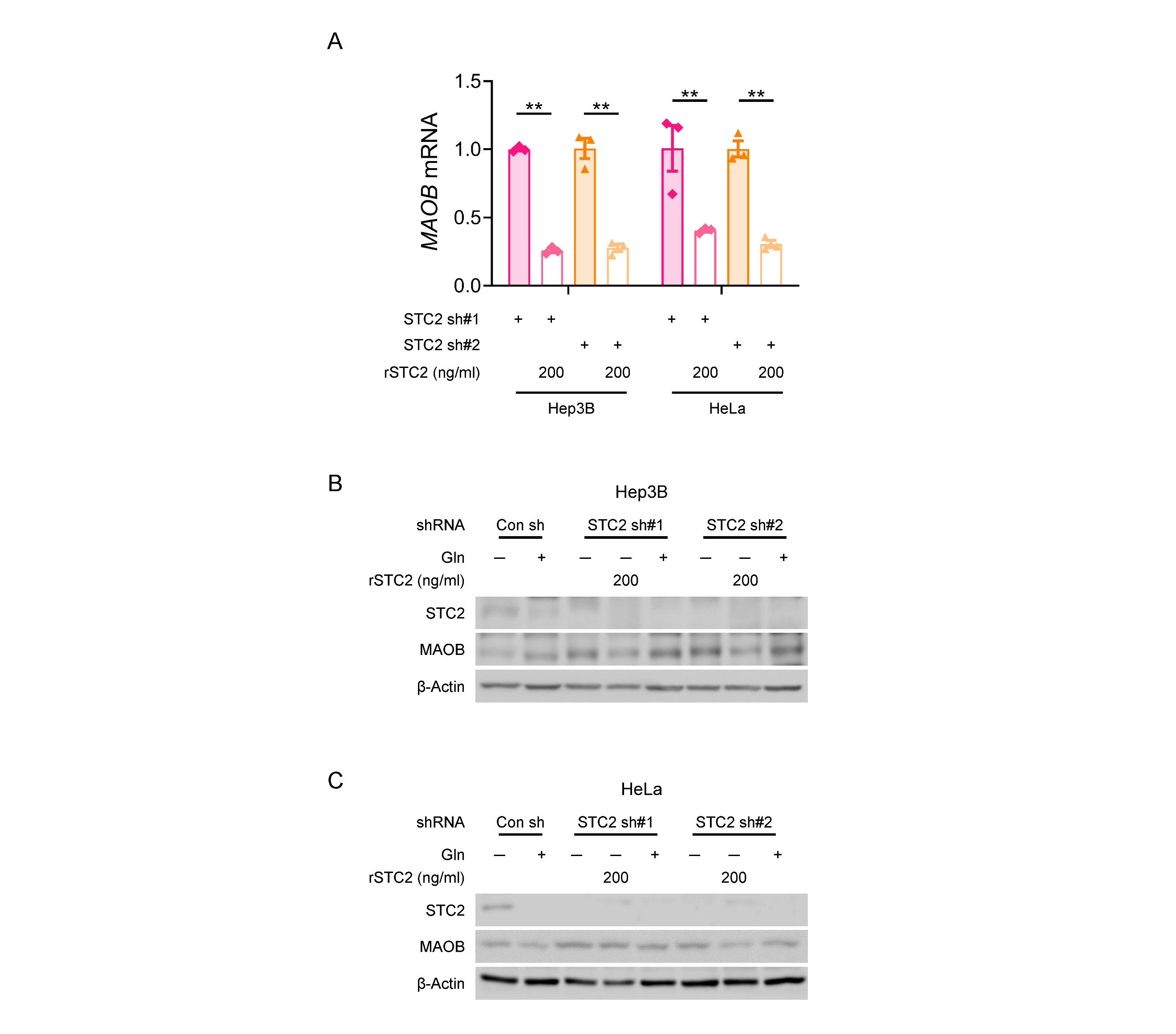


**Figure S14.** rSTC2 in media suppresses MAOB expression in STC2 knockdown cells. **A** Administration of rSTC2 decreases *MAOB* mRNA levels in STC2 knockdown cells. Data are shown as mean ± SD; **, *p*<0.01; n=3. **B, C** rSTC2 treatment decreases MAOB expression in STC2 knockdown Hep3B (B) and HeLa (C) cells exposed to Gln-deprivation.


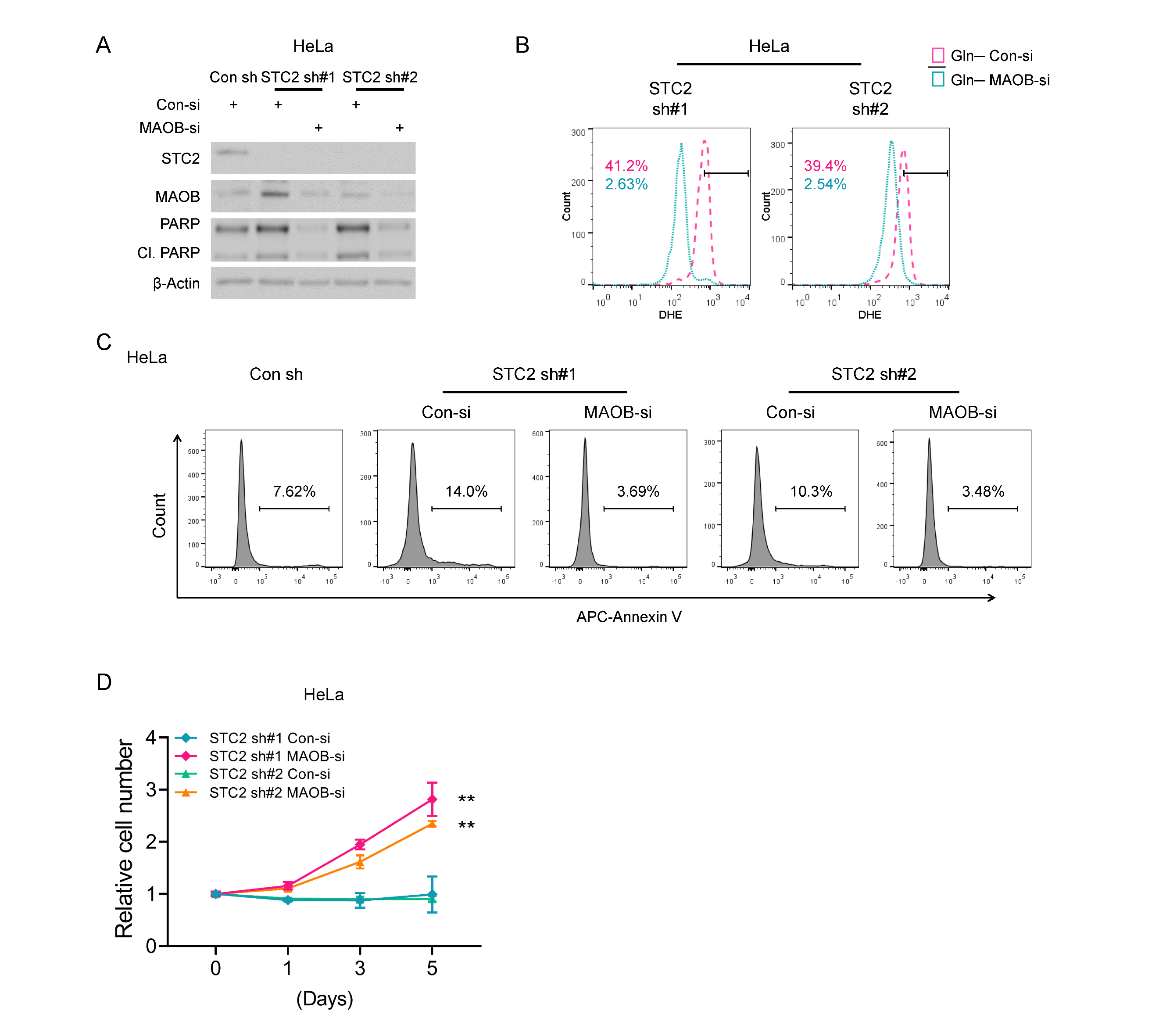


**Figure S15.** MAOB knockdown alleviates Gln-deprivation-triggered apoptosis of STC2 knockdown cells. **A** MAOB knockdown decreases PARP cleavage in STC2 knockdown HeLa cells cultured in Gln-free media. **B** MAOB knockdown decreases ROS levels in STC2 knockdown HeLa cells under Gln-deprived conditions. The numbers indicate the percentage of cells with elevated ROS levels. **C** MAOB knockdown prevents Gln-deprivation-triggered apoptosis of STC2 knockdown HeLa cells. **D** MAOB knockdown rescues the proliferation of STC2 knockdown HeLa cells cultured in Gln-free media. Data are shown as mean ± SD; **, *p*<0.01; n=4.


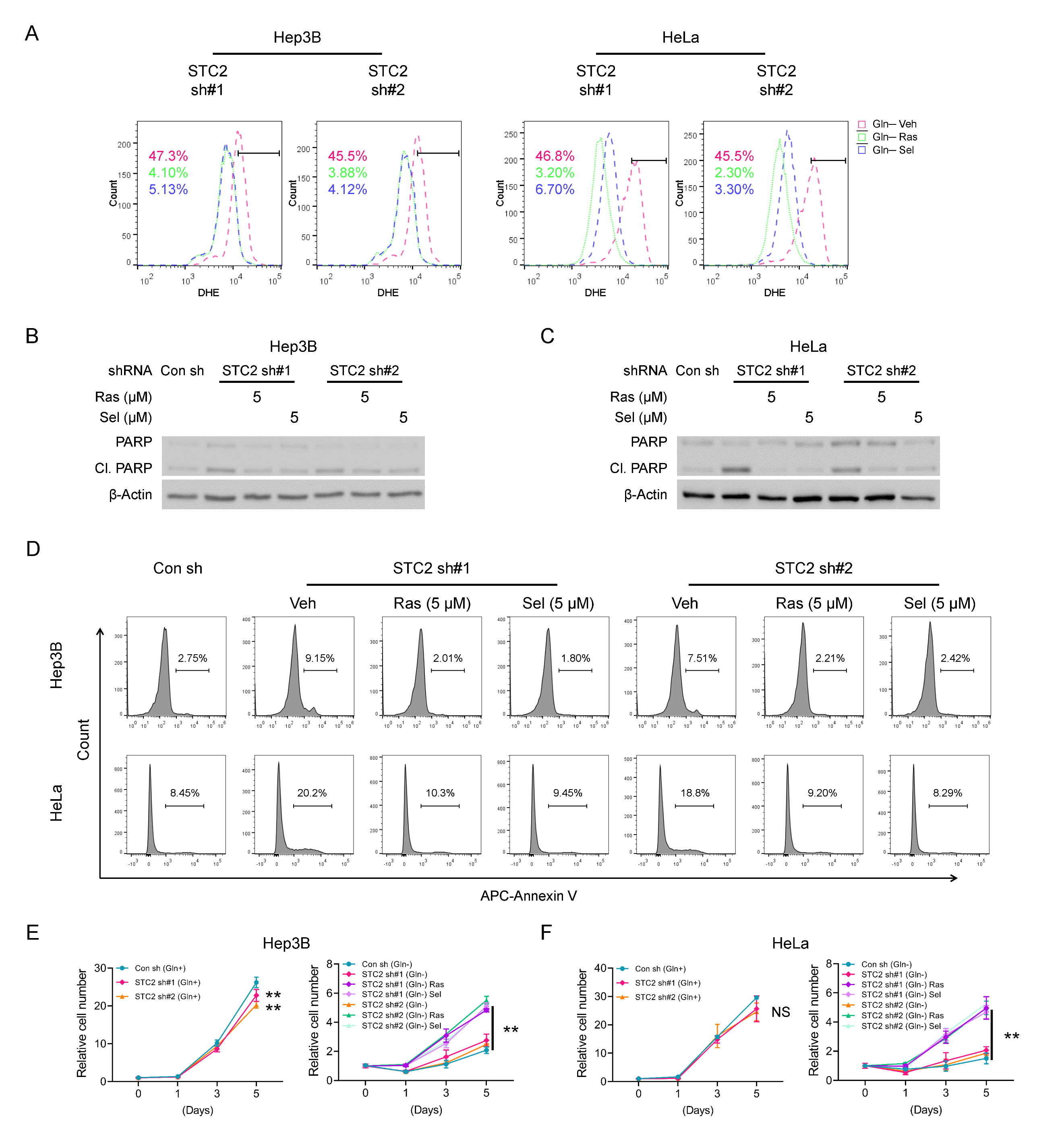


**Figure S16.** MAOB inhibitors prevent Gln-deprivation-triggered apoptosis of STC2 knockdown cells. **A** MAOB inhibitors Rasagiline (Ras) and Selegiline (Sel) decrease ROS levels in STC2 knockdown Hep3B and HeLa cells. The numbers indicate the percentage of cells with elevated ROS levels. **B, C** Ras or Sel reduces PARP cleavage in STC2 knockdown Hep3B (B) and HeLa (C) cells upon Gln-deprivation. **D** Flow cytometry analyses indicate that Ras or Sel inhibits apoptosis of STC2 knockdown Hep3B and HeLa cells cultured in Gln-free media. **E, F** Ras or Sel rescues the proliferation of STC2 knockdown Hep3B (E) and HeLa (F) cells under Gln-deprived conditions. Data are shown as mean ± SD; **, *p*<0.01; n=4.

**
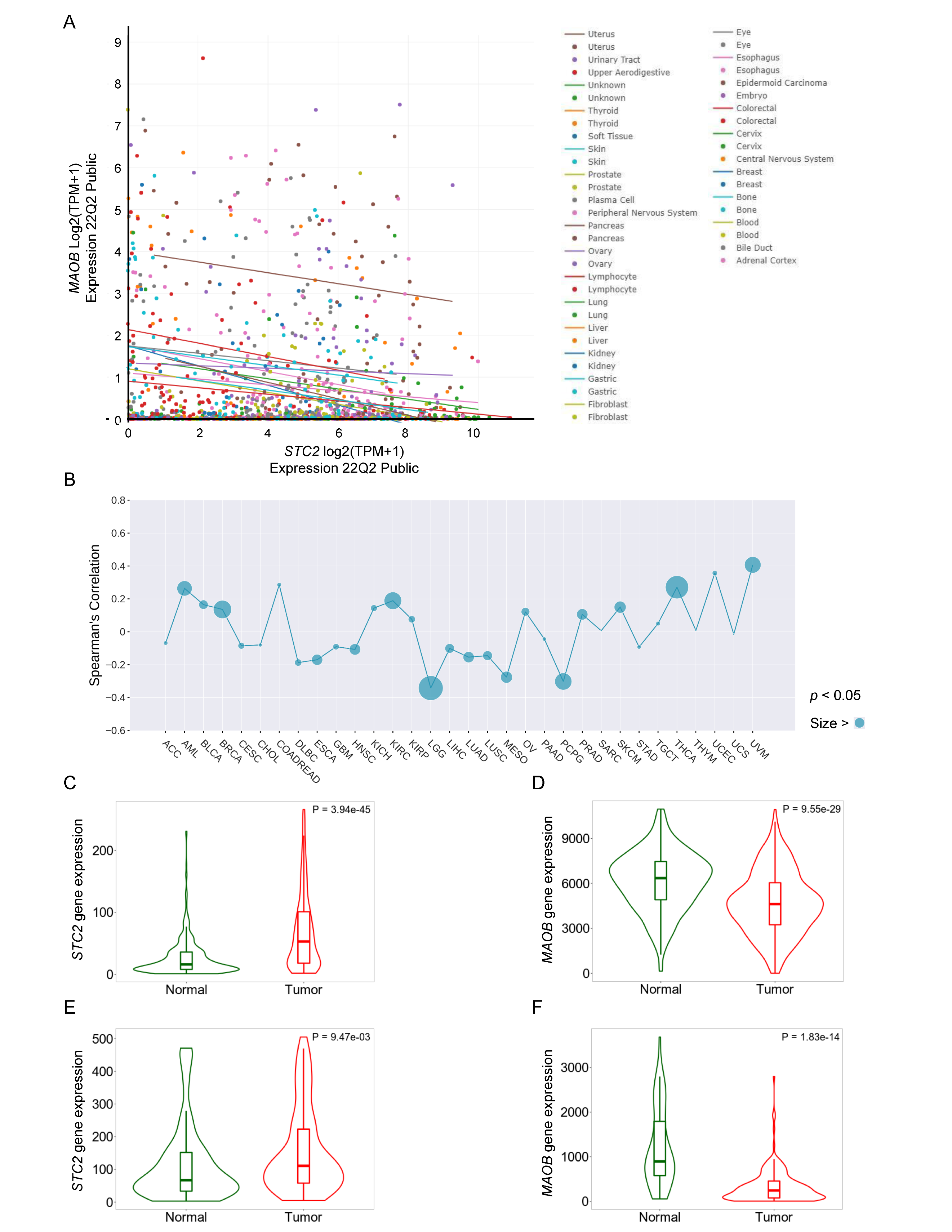
**

**Figure S17.** Bioinformatic analyses reveal a negative correlation between *STC2* and *MAOB* expression. **A** DepMap analyses show a reverse correlation between *STC2* and *MAOB* expression in human tumour cells. **B** cBioPortal analyses reveal a negative correlation between *STC2* and *MAOB* mRNA levels in human HCC and cervical carcinoma tissues. Y-axis indicates the Spearman’s correlation between *STC2* and *MAOB*, the circle size stands for -log10(p value). **C** HCC tumour specimens show increased *STC2* levels relative to normal tissues. **D** HCC tumours specimens show decreased *MAOB* levels relative to normal tissues. **E** Cervical carcinoma specimens show increased *STC2* levels compared to normal tissues. **F** Cervical carcinoma specimens show decreased *MAOB* levels compared to normal tissues.


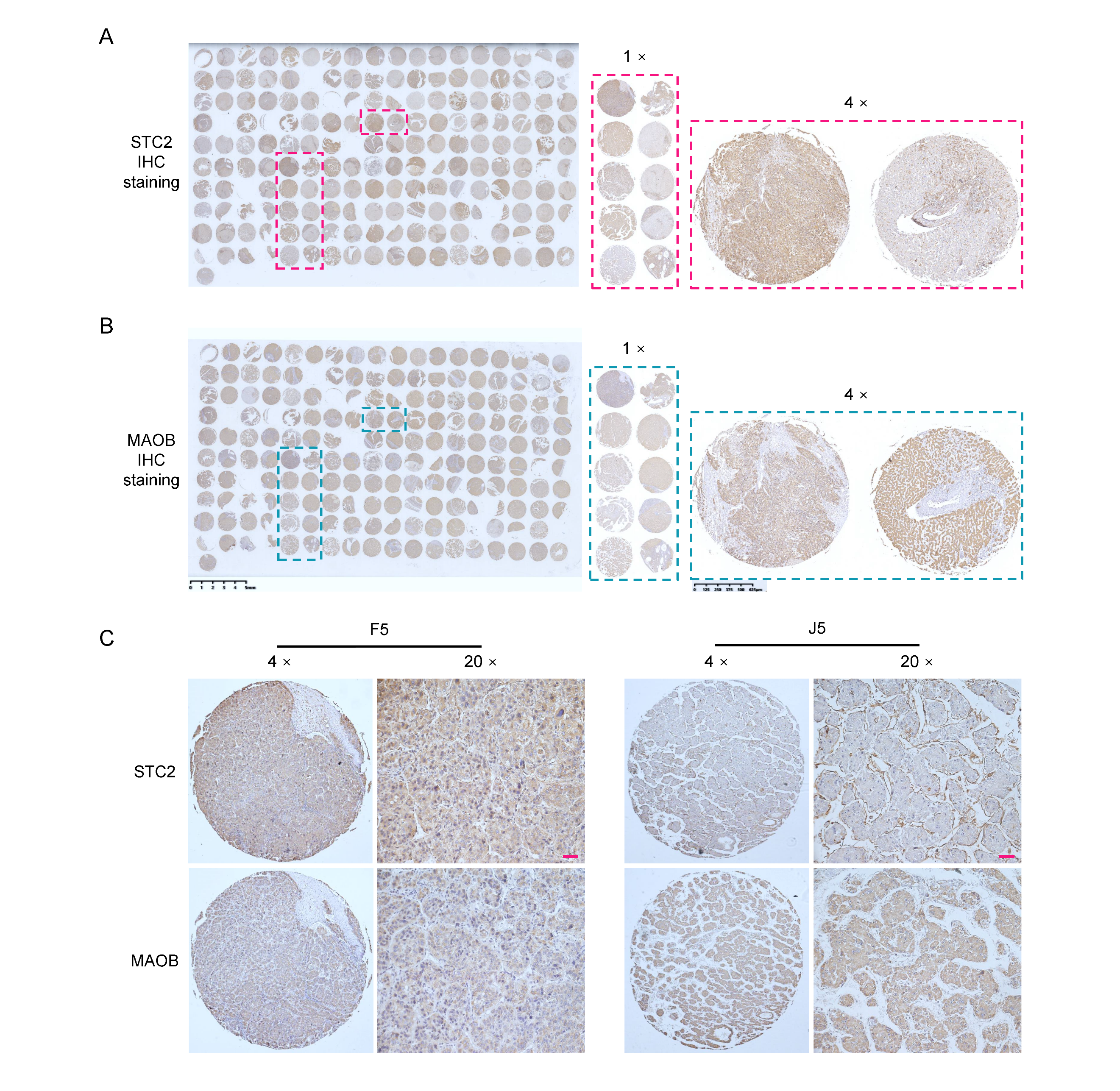


**Figure S18.** STC2 and MAOB levels show reverse correlation in human HCC samples. **A** The whole slide scanning of HCC TMA with STC2 IHC staining. **B** The whole slide scanning of HCC TMA with MAOB IHC staining. **C** Two representative cases show the reverse correlation between STC2 and MAOB expression. Scale bar, 50 μm.

Supplementary Table 1 The upregulated genes with log2(fold change) ≥ 1

in nutrient insufficient comparing to control cells

| Gene | Gln(─)/Hep3B | Glc(─)/Hep3B | MM01/Hep3B |
| --- | --- | --- | --- |
| *S100P* | 4.712066 | 2.696604 | 2.988858 |
| *STC2* | 4.04208 | 2.46978 | 2.641416 |
| *DRD5* | 3.885474 | 2.132124 | 2.047854 |
| *OSTBETA* | 3.88152 | 2.301083 | 2.200525 |
| *MEF2B* | 3.825807 | 2.980603 | 1.993171 |
| *ETV5* | 3.68936 | 1.627603 | 1.904378 |
| *WARS* | 3.650184 | 2.633354 | 1.759502 |
| *FUT1* | 3.55718 | 3.041991 | 1.316837 |
| *GPT2* | 3.378495 | 1.400167 | 1.912175 |
| *TSC22D3* | 3.337839 | 1.681931 | 2.497143 |
| *FUT3* | 3.284884 | 1.730974 | 1.278529 |
| *SLC1A4* | 3.268685 | 1.350868 | 1.558057 |
| *SPIRE1* | 3.260832 | 1.969526 | 1.057879 |
| *SHMT2* | 3.220771 | 1.818831 | 1.392081 |
| *KLHL29* | 3.117394 | 1.401372 | 1.062597 |
| *TRAK2* | 2.99012 | 1.138456 | 1.070688 |
| *C9ORF91* | 2.956149 | 1.930974 | 1.216241 |
| *SESN2* | 2.906565 | 2.013483 | 1.201373 |
| *SLC6A9* | 2.899361 | 1.96307 | 1.691729 |
| *CEBPB* | 2.861664 | 1.841352 | 1.238941 |
| *KCNJ5* | 2.85028 | 1.842299 | 3.054714 |
| *IGFBP3* | 2.833319 | 1.704036 | 1.583106 |
| *PRPH* | 2.813878 | 1.713955 | 2.030949 |
| *FAM129A* | 2.808024 | 2.641583 | 1.376335 |
| *SLC6A15* | 2.804742 | 2.556141 | 1.18747 |
| *MAP2* | 2.667687 | 1.654199 | 1.005133 |
| *HBP1* | 2.666826 | 1.466016 | 1.031591 |
| *CHAC1* | 2.633767 | 2.736532 | 2.256367 |
| *STK40* | 2.601976 | 1.162485 | 1.293106 |
| *MICALL1* | 2.57842 | 1.01788 | 1.049915 |
| *ETV4* | 2.471563 | 1.22638 | 1.643175 |
| *FAM59A* | 2.46781 | 1.107455 | 1.460129 |
| *TAF4B* | 2.233175 | 1.031303 | 1.102567 |
| *COG5* | 2.200804 | 1.041137 | 1.116472 |
| *HSPA13* | 2.096897 | 2.206692 | 1.222955 |
| *THBS4* | 2.08498 | 1.166824 | 1.607208 |
| *ARHGEF2* | 2.082088 | 1.139949 | 1.100641 |
| *GCAT* | 2.047608 | 1.547354 | 1.077735 |
| *INHBE* | 1.936055 | 1.610602 | 1.181499 |
| *SARS* | 1.922609 | 1.971197 | 1.20085 |
| *BEX4* | 1.896043 | 1.296537 | 1.123093 |
| *PCDHA9* | 1.891287 | 1.122945 | 1.335092 |
| *SNTB1* | 1.888267 | 1.229966 | 1.076343 |
| *ASNS* | 1.868988 | 2.061811 | 2.221264 |
| *STXBP1* | 1.860887 | 1.144913 | 1.425236 |
| *BCAT1* | 1.860679 | 1.262712 | 1.5878 |
| *MTHFD1L* | 1.853611 | 1.632051 | 1.182758 |
| *GARS* | 1.785051 | 2.025636 | 1.427624 |
| *RCN1* | 1.759376 | 1.546764 | 1.128413 |
| *DUSP5* | 1.694068 | 1.626926 | 1.123384 |
| *MTHFD2* | 1.690391 | 1.927949 | 1.334463 |
| *SMOX* | 1.682583 | 1.857229 | 1.290372 |
| *MACROD1* | 1.594499 | 1.858742 | 1.502122 |
| *LARP6* | 1.531709 | 2.875743 | 1.839536 |
| *CDKN2B* | 1.420668 | 1.332171 | 1.794742 |
| *ARG2* | 1.327661 | 1.755621 | 1.164393 |
| *TMEM55A* | 1.256989 | 1.740109 | 1.351204 |
| *LRRC49* | 1.249016 | 1.11214 | 1.00748 |

Supplementary Table 2 The NF-κB targeted genes upregulated by Gln-deprivation

|  | Gene | Log2 Ratio | *p*-value |
| --- | --- | --- | --- |
| Hep3B (Gln‒)  *vs*  Hep3B (Gln+) | *IRF7* | 2.634773 | 5.68E-38 |
|  | *G6PD* | 2.141096 | 1.64E-20 |
|  | *RELB* | 2.011901 | 3.37E-34 |
|  | *BCL3* | 1.994334 | 7.19E-21 |
|  | *JUNB* | 1.569589 | 0.00000 |
|  | *REL* | 1.471187 | 5.08E-17 |
|  | *MYC* | 1.470939 | 4.03E-16 |
|  | *ELF3* | 1.463566 | 8.55E-09 |
|  | *GSTP1* | 1.398309 | 2.29E-12 |
|  | *IRF4* | 1.115194 | 8.05E-22 |
|  | *TNFRSF21* | 1.079588 | 2.14E-10 |
|  | *BCL2L11* | 1.056654 | 6.23E-12 |

Supplementary Table 3 The expression change of ROS related genes in STC2 knockdown cells in Gln-free media picked by GSEA from RNA-seq dataset

| Gene | STC2 sh#1  vs  Con sh | | STC2 sh#2  vs  Con sh | |
| --- | --- | --- | --- | --- |
|  | Log2 Ratio | *p*-value | Log2 Ratio | *p*-value |
| *MAOB* | 0.8113 | 3.33E-19 | 0.5788 | 1.30E-09 |
| *GPX2* | 0.5946 | NA | 0.0592 | NA |
| *GPX3* | 0.5780 | 0.303874371 | 0.1621 | 0.823607734 |
| *GPX4* | 0.1680 | 0.012469375 | 0.0274 | 0.784296375 |
| *PRDX6* | 0.1588 | 0.00527803 | 0.2979 | 2.00E-09 |
| *SOD2* | 0.1556 | 0.035176797 | 0.2685 | 2.64E-05 |
| *GCLM* | 0.1315 | 0.140788036 | 0.1336 | 0.119473629 |
| *PRDX2* | 0.0991 | 0.244607809 | 0.2594 | 7.42E-05 |
| *PRDX1* | 0.0898 | 0.128231744 | 0.1791 | 0.000265327 |
| *PRDX5* | 0.0835 | 0.522349466 | -0.0236 | 0.884351049 |
| *PRDX3* | 0.0811 | 0.390623202 | 0.2903 | 1.70E-05 |
| *SOD1* | 0.0154 | 0.899571672 | 0.0749 | 0.371967944 |
| *GPX1* | 0.0036 | 0.976828006 | -0.0236 | 0.812312718 |
| *PRDX4* | -0.0582 | 0.426572848 | -0.0976 | 0.119063212 |
| *GCLC* | -0.0583 | 0.708572856 | 0.1968 | 0.072757612 |

Supplementary Table 4 The primers used in molecular cloning and mutagenesis

|  | Name | Sequences |
| --- | --- | --- |
| *STC2* P2.0 | Sense | 5' AGCCCCCATCCCACTTCTA 3' |
|  | Antisense | 5' CGTTTCTCGTCTTTCCCCTAA 3' |
| *STC2* P2.0 Del1 | Sense | 5' TAGTGCCTGTGCTTACGCC 3' |
|  | Antisense | 5' CGTTTCTCGTCTTTCCCCTAA 3' |
| *STC2* P2.0 Del2 | Sense | 5' AAAAACAGGTGACCAGATGCC 3' |
|  | Antisense | 5' CGTTTCTCGTCTTTCCCCTAA 3' |
| *STC2* P2.0 Del3 | Sense | 5' TTTGTACCCGCACGTCCTT 3' |
|  | Antisense | 5' CGTTTCTCGTCTTTCCCCTAA 3' |
| *STC2* P2.0 Del4 | Sense | 5' GGGACACGGGAAAGGAAA 3' |
|  | Antisense | 5' CGTTTCTCGTCTTTCCCCTAA 3' |
| *STC2* P2.0 Del5 | Sense | 5' GCCCAACTTTCTCCTTCCC 3' |
|  | Antisense | 5' CGTTTCTCGTCTTTCCCCTAA 3' |
| *STC2* P1.4 | Sense | 5' CACACACAGCAGGGGAAGA 3' |
|  | Antisense | 5' GGTCAAAGGTGGCCAACA 3' |
| *STC2* P2.0 Del4 NF-κB mutation | Sense | 5' AGTGGGAGTTGAAACTTCCCAGGCGC 3' |
|  | Antisense | 5' AGTTTCAACTCCCACTGCCCAGGCGAC 3' |
| *STC2* P2.0 Del4 ATF4 mutation | Sense | 5' TGAAACTGTAGAGGCGCATTACCGCAG 3' |
|  | Antisense | 5' TGCGCCTCTACAGTTTCATCACCCACT 3' |
| *STC2* P2.0 Del4+P1.4 | Sense | 5' GGGACACGGGAAAGGAAA 3' |
|  | Antisense | 5' GGTCAAAGGTGGCCAACA 3' |

Supplementary Table 5 The primers used in ChIP analysis

|  | Name | Sequences |
| --- | --- | --- |
| *STC2* | Sense | 5' CCGGC CAAAA GATGA CCTGC 3' |
|  | Antisense | 5' TGCGC CTGGG AAGTT TCATC A 3' |
| *ASNS* | Sense | 5' AAACA GGCGC ACTGA GACGC 3' |
|  | Antisense | 5' GCGTG CGGGA AGTTT CATCA T 3' |
| *IL-8* | Sense | 5' GGCCA TCAGT TGCAA AT 3' |
|  | Antisense | 5' TTCCT TCCGG TGGTT TCTTC 3' |
| *GAPDH* | Sense | 5' TACTA GCGGT TTTAC GGGCG 3' |
|  | Antisense | 5' TCGAA CAGGA GGAGC AGAGA GCGA 3' |
