# Supplementary material for: Stanniocalcin 2 governs cancer cell adaptation to nutrient insufficiency through alleviation of oxidative stress
Source: Cell Death Dis. 2024 Aug 6;15(8):567. doi: 10.1038/s41419-024-06961-7 (PMC11303387; doi:10.1038/s41419-024-06961-7)
Supplement: Supplementary file 2 — STC2 Supplementary Materials and Methods [file 41419_2024_6961_MOESM2_ESM.docx]

**Supplementary Materials and Methods**

**Stanniocalcin 2 governs cancer cell adaptation to nutrient insufficiency through alleviation of oxidative stress**

Shuo Qie^1,2,3,4,#,*^, Haijuan Xiong^1,2,3,4,^**^#^**, Yaqi Liu^1,2,3,4,^**^#^**, Chenhui Yan^1,2,3,4^, Yalei Wang^1,2,3,4^, Lifeng Tian^5^, Chenguang Wang^5^ and Nianli Sang^6,*^

1 Department of Pathology, Tianjin Medical University Cancer Institute and Hospital, Tianjin, 300060 China

2 National Clinical Research Center for Cancer, Tianjin, 300060 China

3 Key Laboratory of Cancer Prevention and Therapy (Tianjin), Tianjin, 300060 China

4 Tianjin's Clinical Research Center for Cancer, Tianjin, 300060 China

5 Department of Cancer Biology, Kimmel Cancer Center, Thomas Jefferson University, Philadelphia, PA, 19107 USA

6 Department of Biology, Drexel University, Philadelphia, PA, 19104 USA

**^#^**, These authors contributed equally to this work.

^*,^ Corresponding authors:

Shuo Qie: Department of Pathology, Tianjin Medical University Cancer Institute and Hospital, Huanhuxi Road, Tiyuanbei, Hexi District, Tianjin 300060, China. Email: shuoqie@tmu.edu.cn

Nianli Sang: Department of Biology, Drexel University, Papadakis Integrated Sciences Building, Room 417, 3245 Chestnut St, Philadelphia, PA 19104, USA. Email: nianli.sang@drexel.edu

**Reagents and antibodies**

Recombinant human STC2 protein was purchased from R&D Systems (9405-SO-050). (Minneapolis, MN, USA) L-glutamine (200 mM) was purchased from Corning Inc. (Glendale, AZ, USA). Following reagents were purchased from Sigma-Aldrich (St. Louis, MO, United States): D-Glucose (Glc), NH_4_Cl, L-6-diazo-5-oxo-L-norleucine (DON), Actinomycin D (ActD), Cycloheximide (CHX), Curcumin, Tumour necrosis factor-α (TNF-α), Tunicamycin (TM), Tharpasigagin (Tg), Brefeldin A (BFA), and MG132. BAY 11-7082 and Phorbol-12-myristate-13-acetate (PMA) were purchased from EMD Chemicals (Port Wentworth, GA, USA). N-Acetyl-L-cysteine ethyl ester (NAC), L-Glutathione (GSH), Rasagiline and Selegiline hydrochloride were purchased from MedChemExpress (NJ, USA).

Goat anti-STC2 (AF2830) polyclonal antibody was purchased from R&D Systems, rabbit anti-STC2 (10314-1-AP), ASNS (14681-1-AP), VEGFA (19003-1-AP), MAOB (12602-1-AP) and Ki-67 (27309-1-AP) polyclonal antibodies and mouse anti-MAOB (66107-1-Ig) and β-actin (66009-1-Ig) monoclonal antibodies were purchased from Proteintech (Rosemont, IL, USA), mouse anti α-tubulin (T6199) monoclonal antibody was purchased from Sigma-Aldrich, rabbit anti-PARP (9542), p-eIF2α (3398), total eIF2α (2103), ATF4 (11815), p65/RelA (8242) and cleaved caspase-3 (9661) monoclonal antibodies were purchased from Cell Signaling Technology (Danvers, MA, USA), rabbit anti HDAC2 (3602) polyclonal antibody was purchased from BioVision (Cambridge, UK), rabbit anti LC3B (NB100-2220) polyclonal antibody was purchased from Novus Biologicals (Centennial, CO, USA).

**Gln-deprivation assay**

Cells were first maintained in Gln-free DMEM supplemented with 10% dialyzed FBS plus 4 mM Gln. On the next day, fresh media (Gln-free DMEM with 10% dialyzed FBS) were added and kept for indicated periods. Cells cultured in Gln-free DMEM with 10% dialyzed FBS and 4 mM Gln were used as controls.

**cDNA microarray and RNA-Seq analysis**

For microarray study, total RNA samples were collected from Hep3B cells (Gln-/Glc- deprived conditions), MM01 cells (Gln-free +0.8 mM NH_4_^+^) and Hep3B cells (regular media). Gene expression profile was analysed by GenePix Pro version 4.0 (OneArrays platform from the Phalanx Biotech Group, San Diego, CA, USA). GenePix data were analysed using Array Studio (Omicsoft Corp, Cary, NC, USA). The significant differential expression was defined as p value < 0.05 and | log2(fold changes) | ≥ 1 [^1^](#_ENREF_1). The iDEP (integrated differential and pathway analysis) online tool was employed to analyse microarray data (http://bioinformatics.sdstate.edu/idep/) [^2^](#_ENREF_2).

For RNAseq assays, total RNA samples were prepared using TRIzol reagent from Hep3B cells infected with lentivirus expressing control shRNA, STC2 shRNA #1 and STC2 shRNA #2 and cultured in regular or Gln-free media. RNA library and transcriptome sequencing were analysed by Novogene Co. (Beijing, China). The iDEP (integrated differential and pathway analysis) online tool was employed to analyse RNA-Seq data (http://bioinformatics.sdstate.edu/idep/)[^2^](#_ENREF_2). The transcriptomic dataset was normalized and log2(fold changes) was calculated using the iDEP v0.96. Gene set enrichment analysis (GSEA) was applied to analyse the gene express signatures using the Hallmark and REACTOME gene sets [^3^](#_ENREF_3).

**Pan-Cancer survival analysis**

Pan-Cancer Survival Analysis of *STC2* across 21 types of tumours were analysed using the Kaplan-Meier plotter analysis (https://kmplot.com/analysis/) [^4^](#_ENREF_4).

**Other bioinformatic tools**

DepMap analysis was conducted to study the expression correlation between *STC2* and *MAOB* using DepMap portal (<https://depmap.org>). For cBioportal and TNMplot analyses, the expression correlation between *STC2* and *MAOB* was listed and presented using the online tool (http://www.cbioportal.org/) [^5^](#_ENREF_5)^,^ [^6^](#_ENREF_6). For TNMplot analysis, gene expression comparison was performed on the gene chip data using the online tool (https://tnmplot.com/) [^7^](#_ENREF_7).

**Western blot analysis**

Whole cell lysate was extracted using either urea lysis buffer (6.6 M urea, 10 mM Tris, 1% SDS, 5 mM DTT, 1% Triton X-100, 10% Glycerol) or RIPA lysis buffer (25 mM Tris, 150 mM NaCl, 1% NP-40, 1% sodium deoxycholate, 0.1% SDS) supplemented with both protease and phosphatase inhibitors as indicated in experiments. The same amount of whole cell lysate was resolved by SDS-PAGE gels, and thereafter transferred to PVDF membranes. After being blocked by 5% nonfat milk in 1× Tris Buffered Saline with Tween 20 (TBST)**,** the membranes were incubated with primary antibodies overnight. After 3 washes with 1x TBST, membranes were incubated with HRP-conjugated secondary antibodies at room temperature. Finally, signals were visualized using the chemiluminescence reagents (Biosharp, Anhui, China).

**Quantitative Real Time-PCR (qRT-PCR)**

Total RNA was extracted using RNeasy Mini Kit (QIAGEN, Hilden, Germany). Then, total RNA was reverse-transcribed to cDNA using SuperScript II Reverse Transcriptase or RevertAid First Strand cDNA Synthesis Kit (Thermo Fisher Scientific, Waltham, MA, USA). The cDNA was diluted at a ratio of 1/50 and applied for quantitative analysis using validated gene-specific TaqMan probes (Applied Biosystem, Woburn, MA, USA). *β-actin* or *GAPDH* were detected as housekeeping genes for normalization. The relative fold changes were calculated using 2^-ΔΔCt^ method.

**Molecular cloning**

pCMV6 vector with human *STC2* cDNA was purchased from Origene. Genomic DNA was collected from HeLa cells using Wizard Genomic DNA Purification Kit (Promega Corporation, Fitchburg, WI, USA). The primers for cloning and mutagenesis of *STC2* promoter are listed in Supplementary Table 4. *STC2* promoter P2.0 and its relative deletions and *STC2* promoter P1.4 were ligated to pGL3-Luciferase Basic Vector (Promega Corporation). All plasmids were confirmed by automatic sequencing.

**Luciferase reporter assay**

For luciferase assay, pRL*-*CMV Renilla luciferase vector (Promega Corporation) was co-transfected with constructed firefly luciferase plasmids. On the next day, cells were treated with media including 0 or 4 mM Gln and TM or Tg for 6 h. Cells were lyzed using 1× Passive Lysis Buffer (Promega Corporation). Dual-Luciferase Reporter Assay was performed using BioSystems Luminometer Model TD-20/20.

**Chromatin immunoprecipitation (ChIP) assay**

Hep3B cells were treated in media with/without 4mM Gln for 5 h. ChIP was performed using EZ-ChIP kit (EMD Millipore, Danvers, MA, USA) by following the protocol provided by the manufacture. Chromatin shearing was performed using the SCIENTZ-IID sonicator. The same amount sheared chromatin was applied for ChIP assay using normal rabbit IgG (CST # 2729), ATF4 (CST # 11815) or p65/RelA (CST # 8242) antibodies. Eluted DNA fragments were employed for PCR amplification. The primers used in PCR are listed in Supplementary Table 5.

**Transfection**

*ATF4* and control siRNAs were purchased from Ambion (Waltham, MA, USA); *p65/RelA* siRNA was bought from Cell Signaling Technology; *MAOB* siRNA was purchased from Santa Cruz Biotechnology (Dallas, TX, USA). Prior to transfection, 3×10^5^ cells were plated into 6 cm dishes. The next day, cells were transfected using Lipofectamine 2000 transfection reagent following manufacture’s protocol. Six hours post transfection, complete media was supplied. On the third day, a second transfection was performed. Twenty-four hours after the second transfection, cells were plated for indicated experiments.

**Lentivirus production and infection**

Plasmids carrying control or STC2 shRNAs were purchased from Horizon Discovery Ltd (https://horizondiscovery.com/). Lentivirus was produced by co-transfecting HEK293T cells with psPAX2, pCMV-VSV-G (Addgene, Watertown, MA, USA) and shRNA plasmids. The supernatant was collected and filtered at 48 h and 72 h post transfection. Infection was performed using supernatant with control or STC2 shRNA lentiviruses and puromycin was applied to establish stable cell lines.

**Flow cytometry analysis**

For apoptosis analysis, after relative treatment, cells were trypsinized and washed with ice cold PBS. Then, the cells were stained with Annexin V-APC/7-AAD apoptosis kit (MultiSciences Biotech Co., Zhejiang, China). For cell cycle analysis, cells were trypsinized and washed with ice cold PBS after relative treatment. Then, cells were stained with Cell cycle staining kit (MultiSciences Biotech Co.). The stained cells were analysed using BD FACS Canto II flow cytometer, and the data were analysed using FlowJo software (Tree Star, Inc., Ashland, OR, USA).

**Cell proliferation assay**

For Trypan blue assays: Cells were seeded at a density of 1×10^5^/6 cm dish. 24 h later cells were treated with media specified in each experiment. Seventy-two hours after treatment, cells were trypsinized and stained with 0.4% Trypan Blue solution (w/v) for 3 min. Cell numbers were counted using hematocytometer.

For CyQUANT® NF Cell Proliferation Assay: Cells were seeded at a density of 1×10^3^ per well in 96-well plates. 24 h later, cell numbers in one plate were determined using the CyQUANT® NF Cell Proliferation Assay Kit (Thermo Fisher Scientific) and used as Day 0. All other plates were undergone different treatments and cell numbers of each group were determined for Day 1, Day 3 and Day 5. The “Relative Cell Numbers” were calculated by dividing the numbers on Day 1, 3 or 5 by that on Day 0.

**Immunohistochemical (IHC) staining**

Paraffin-embedded sections were deparaffinized, steamed, blocked with 5% goat serum (ZSGB-Bio, Beijing, China) in 1× TBST, and incubated with STC2, MAOB, Ki-67 and cleaved caspase-3 antibodies, respectively. Normal rabbit IgG was used as negative control. Signals were amplified using ZSGB-Bio Kit (PV-6001) and visualized by DAB substrate (ZLI-9019). Following IHC staining, all sections were counterstained with hematoxylin, dehydrated and mounted.

**Quantification of the IHC slides**

For pathological analyses, slides were assessed by two independent pathologists. For Ki-67 and cleaved caspase-3, sections were first checked under low power field to choose appropriate fields for quantification. Then, IHC staining index was assessed at 60 × field, and numbers of cleaved caspase-3 positive cells per 400 cells was used to represent apoptosis rates, and percentage of Ki-67 positive cells was evaluated to assess cell proliferation.

The IHC slides were evaluated by two pathologists independently for staining intensity and the percentage of cells in each group. For STC2 and MAOB, the staining intensity was determined to present the expression levels of STC2 and MAOB. Positive intensity scores were defined by overall staining color: 0 = no stain, 1 = light yellow, 2 = yellow and 3 = brown. The percentage of positively stained cells was also evaluated in the form of %. The staining index was calculated by the sum of the products of intensity multiplied by the percentage of cells in that group, and divided by 2.

**Measurement of mitochondrial activity and ROS levels**

For mitochondrial activity determination, cells were trypsinized and washed with ice cold PBS after treatments. Then cells were stained with Mitochondria Staining Kit (JC-1) (MultiSciences Biotech Co.). For ROS detection, cells were trypsinized and washed with ice cold PBS after treatments, followed by staining with Reactive Oxygen Species Assay Kit (Applygen, Beijing, China). Stained cells were analysed using BD FACS Canto II flow cytometer. All results were analysed and quantified using FlowJo software (Tree Star, Inc.).

**MitoSOX staining and flow cytometric analyses**

MitoSOX dye is applied to detect mitochondrial ROS. Particularly, MitoSOX dye (MedChemExpress) is absorbed by cells and is oxidized by mitochondrial superoxide but not by other ROS. In general, cells were first treated, and collected by trypsinization. Then, cells were stained using 2.5 μM MitoSOX in working solution for 30 min. After staining, cells were washed by PBS and resuspended for flow cytometric analysis using Excitation/emission wavelength: 510/580 nm. Finally, the data were analysed using FlowJo software (Tree Star, Inc., Ashland, OR, USA).

**Immunofluorescent staining**

Hep3B cells were seeded the day before treatment. After 24 h Gln-deprivation, cells were fixed, blocked with 5% goat serum (ZSGB-Bio), and incubated with p65/RelA primary antibody (CST # 8242). Paraffin-embedded HCC sections were deparaffinized, blocked with 5% goat serum (ZSGB-Bio) in 1× TBST and incubated with rabbit anti-STC2 polyclonal (10314-1-AP) and mouse anti-MAOB monoclonal (66107-1-Ig) primary antibodies at the same time. Normal rabbit and mouse IgGs were used as a negative control. The signals were visualized using Goat-anti Rabbit-Alexa Fluor® 488 and Goat-anti Mouse-Alexa Fluor® 594 (ZSGB-Bio, Beijing, China), respectively. Following immunofluorescent staining, all sections were mounted by ProLong Diamond Antifade Mountant with DAPI (Thermo Fisher Scientific) and sealed by nail polish.

**References**

1. Qie S, Liang D, Yin C, Gu W, Meng M, Wang C*, et al.* Glutamine depletion and glucose depletion trigger growth inhibition via distinctive gene expression reprogramming. *Cell Cycle* 2012, **11**(19)**:** 3679-3690.

2. Ge SX, Son EW, Yao R. iDEP: an integrated web application for differential expression and pathway analysis of RNA-Seq data. *BMC Bioinformatics* 2018, **19**(1)**:** 534.

3. Mootha VK, Lindgren CM, Eriksson KF, Subramanian A, Sihag S, Lehar J*, et al.* PGC-1alpha-responsive genes involved in oxidative phosphorylation are coordinately downregulated in human diabetes. *Nat Genet* 2003, **34**(3)**:** 267-273.

4. Gyorffy B. Discovery and ranking of the most robust prognostic biomarkers in serous ovarian cancer. *Geroscience* 2023, **45:** 1889-1898.

5. Cerami E, Gao J, Dogrusoz U, Gross BE, Sumer SO, Aksoy BA*, et al.* The cBio cancer genomics portal: an open platform for exploring multidimensional cancer genomics data. *Cancer Discov* 2012, **2**(5)**:** 401-404.

6. Gao J, Aksoy BA, Dogrusoz U, Dresdner G, Gross B, Sumer SO*, et al.* Integrative analysis of complex cancer genomics and clinical profiles using the cBioPortal. *Sci Signal* 2013, **6**(269)**:** pl1.

7. Bartha A, Gyorffy B. TNMplot.com: A Web Tool for the Comparison of Gene Expression in Normal, Tumor and Metastatic Tissues. *Int J Mol Sci* 2021, **22**(5)**:** 2622.
