# Supplementary material for: Stanniocalcin 2 governs cancer cell adaptation to nutrient insufficiency through alleviation of oxidative stress
Source: Cell Death Dis. 2024 Aug 6;15(8):567. doi: 10.1038/s41419-024-06961-7 (PMC11303387; doi:10.1038/s41419-024-06961-7)

Fig. 1c

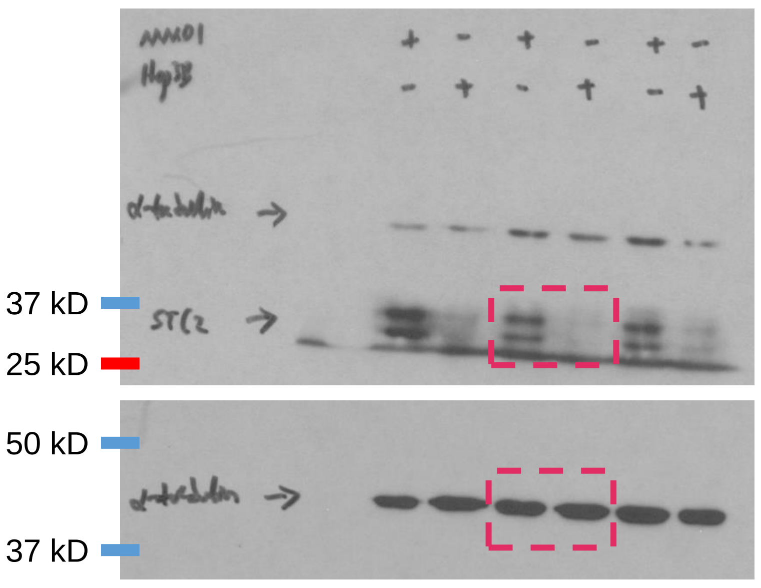

Fig. 1d

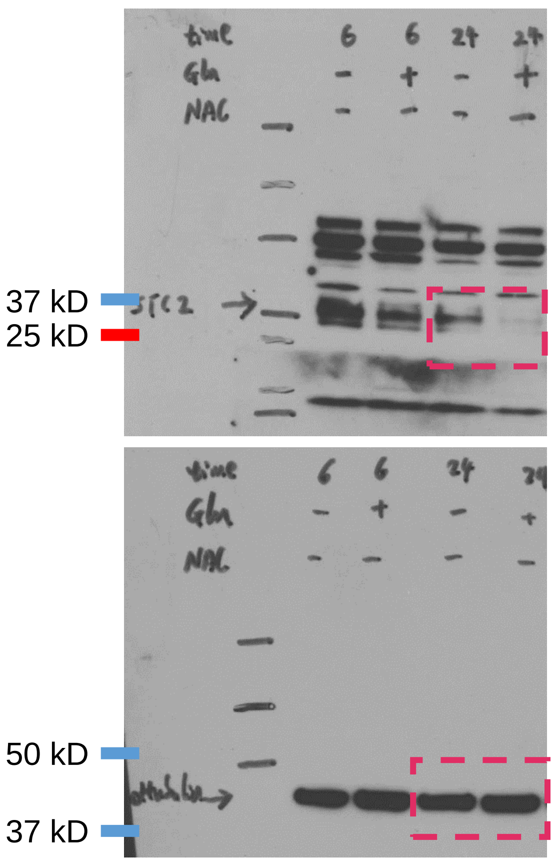

Fig. 1e

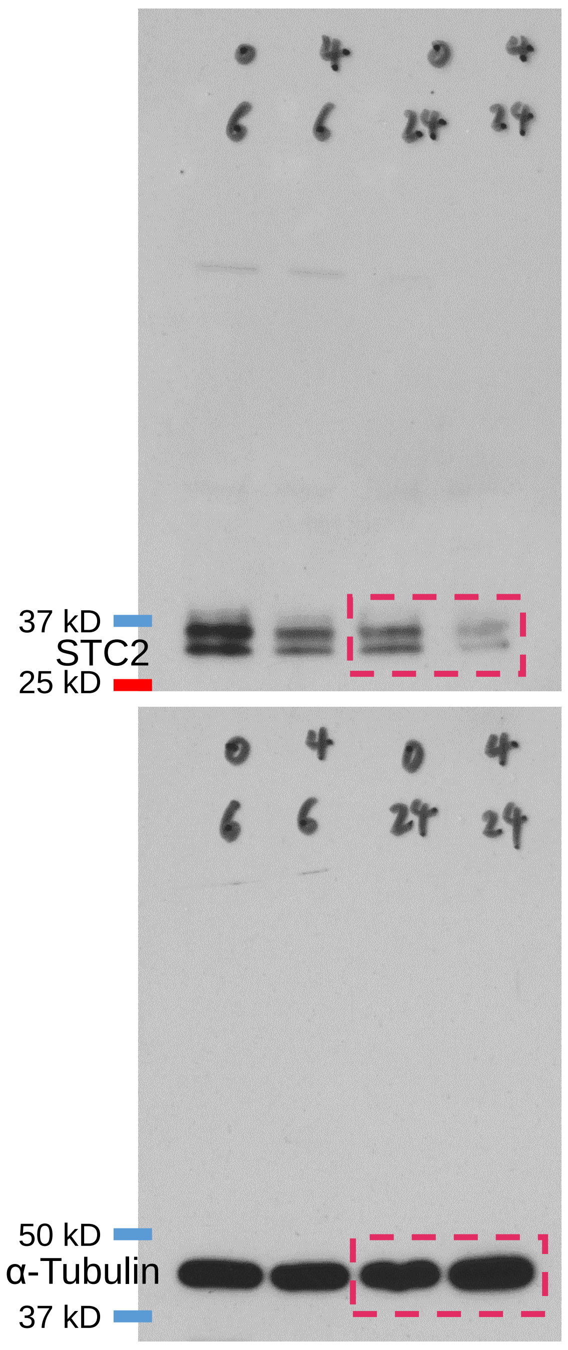

Fig. 1g

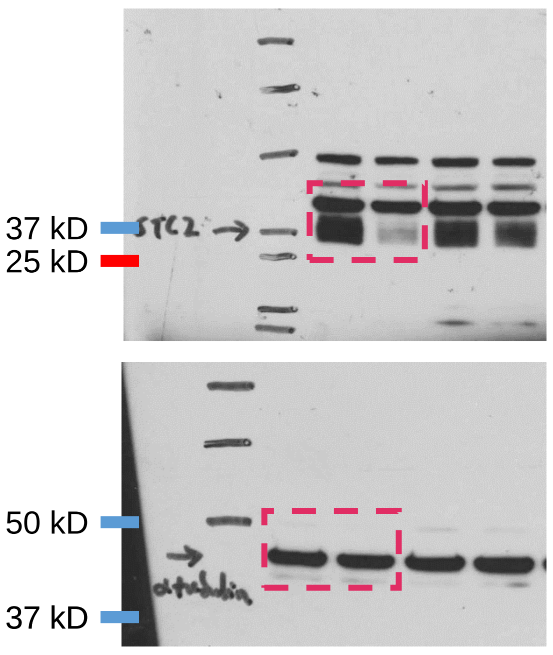

Fig. 1h

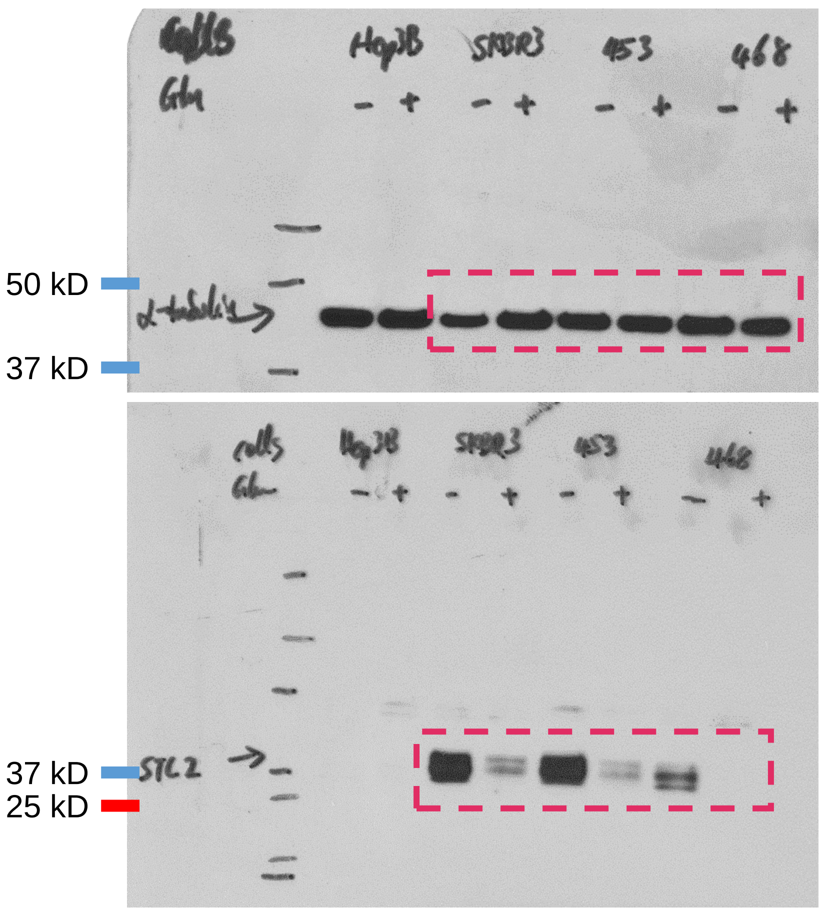

Fig. 1i

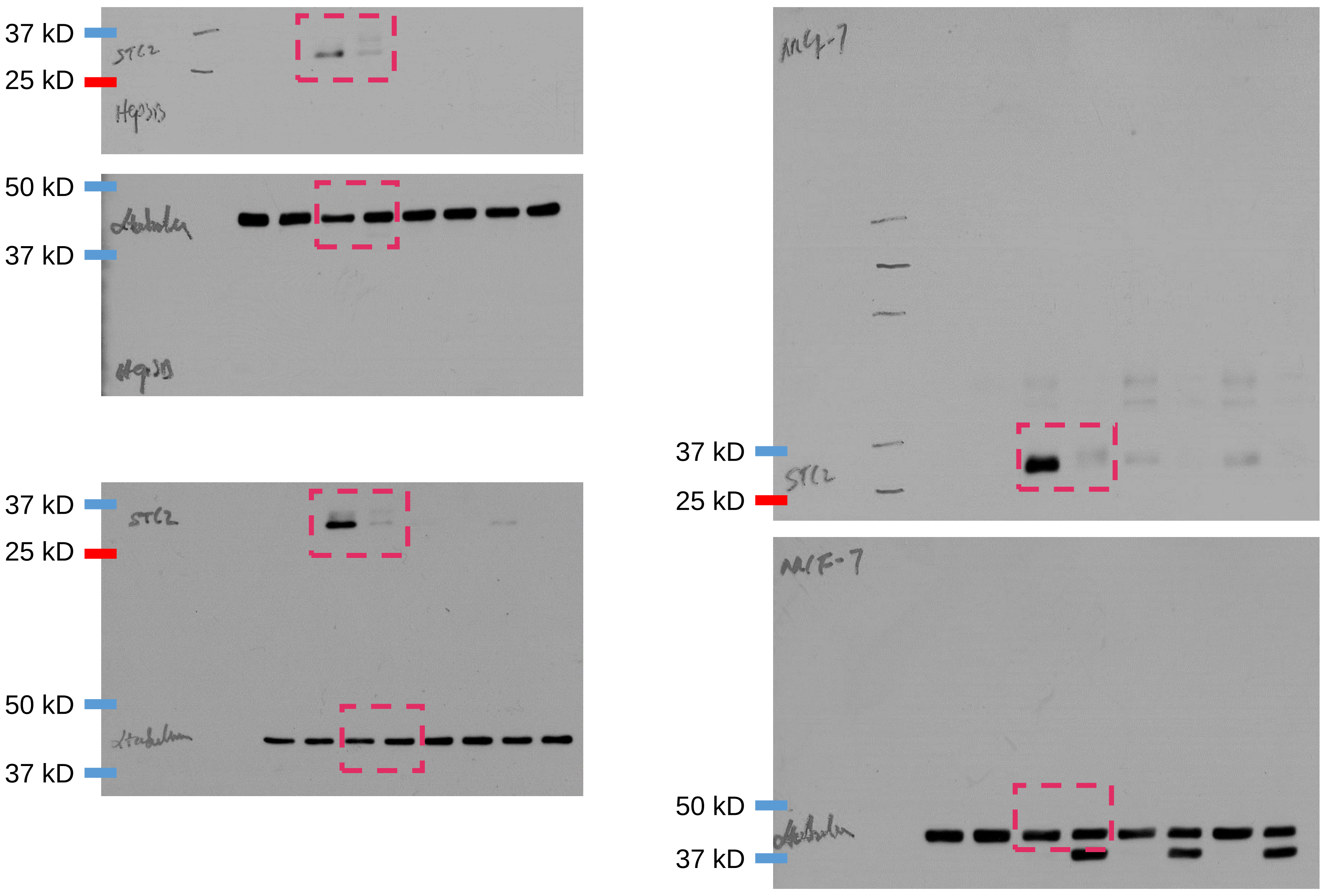

Fig. 1j

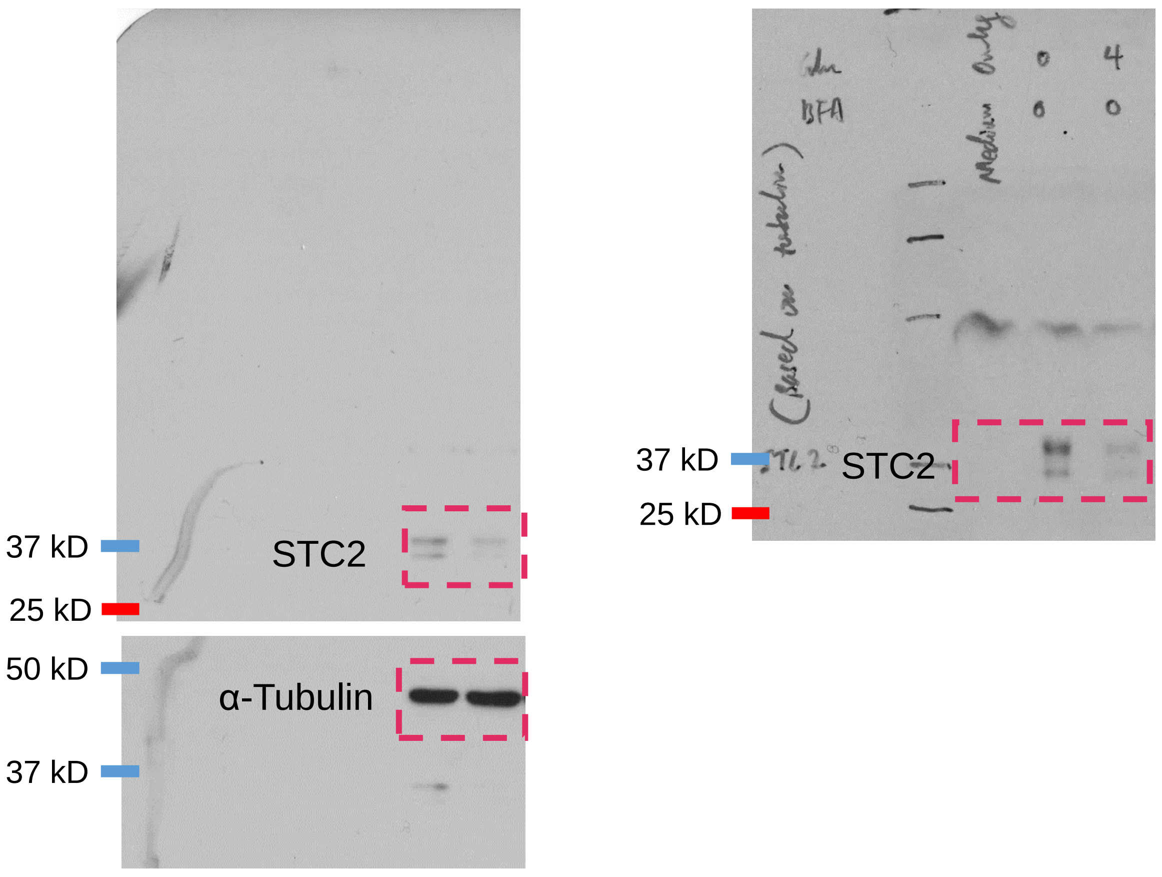

Fig. 1k

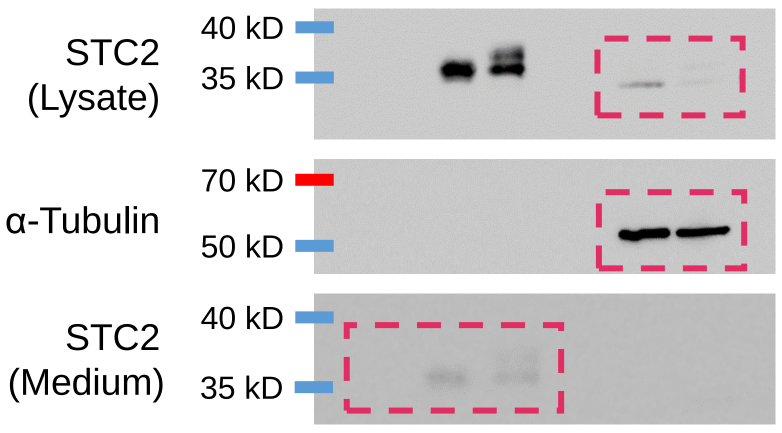

Fig. 1l

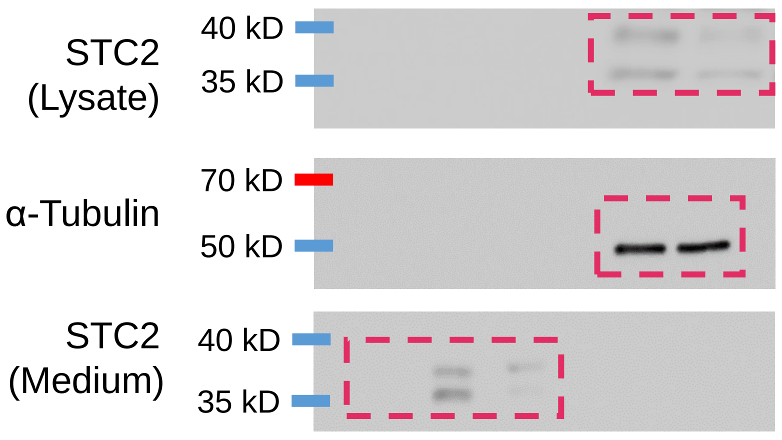

Fig. 1m

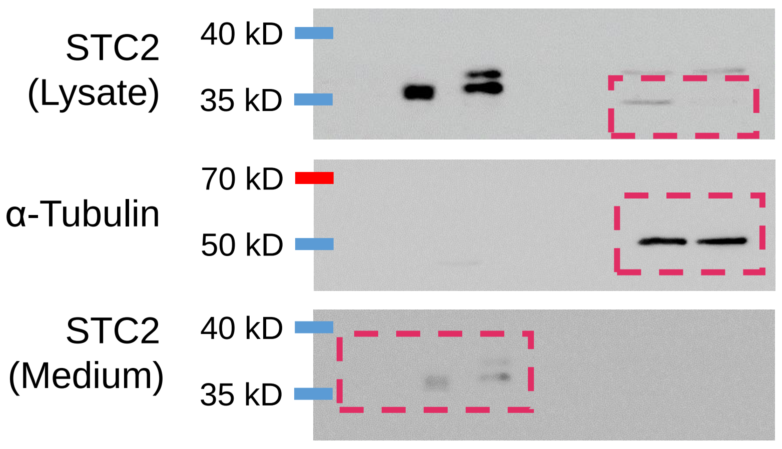

Fig. 2b

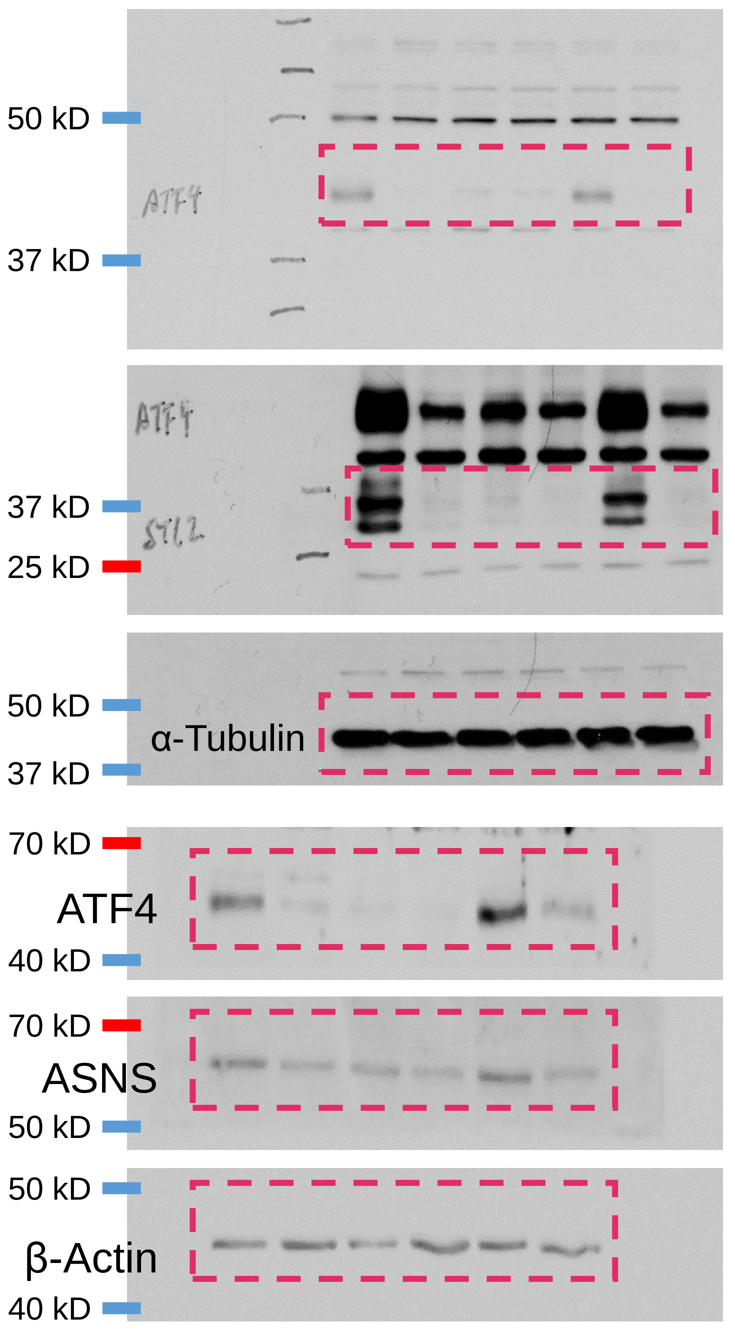

Fig. 2c

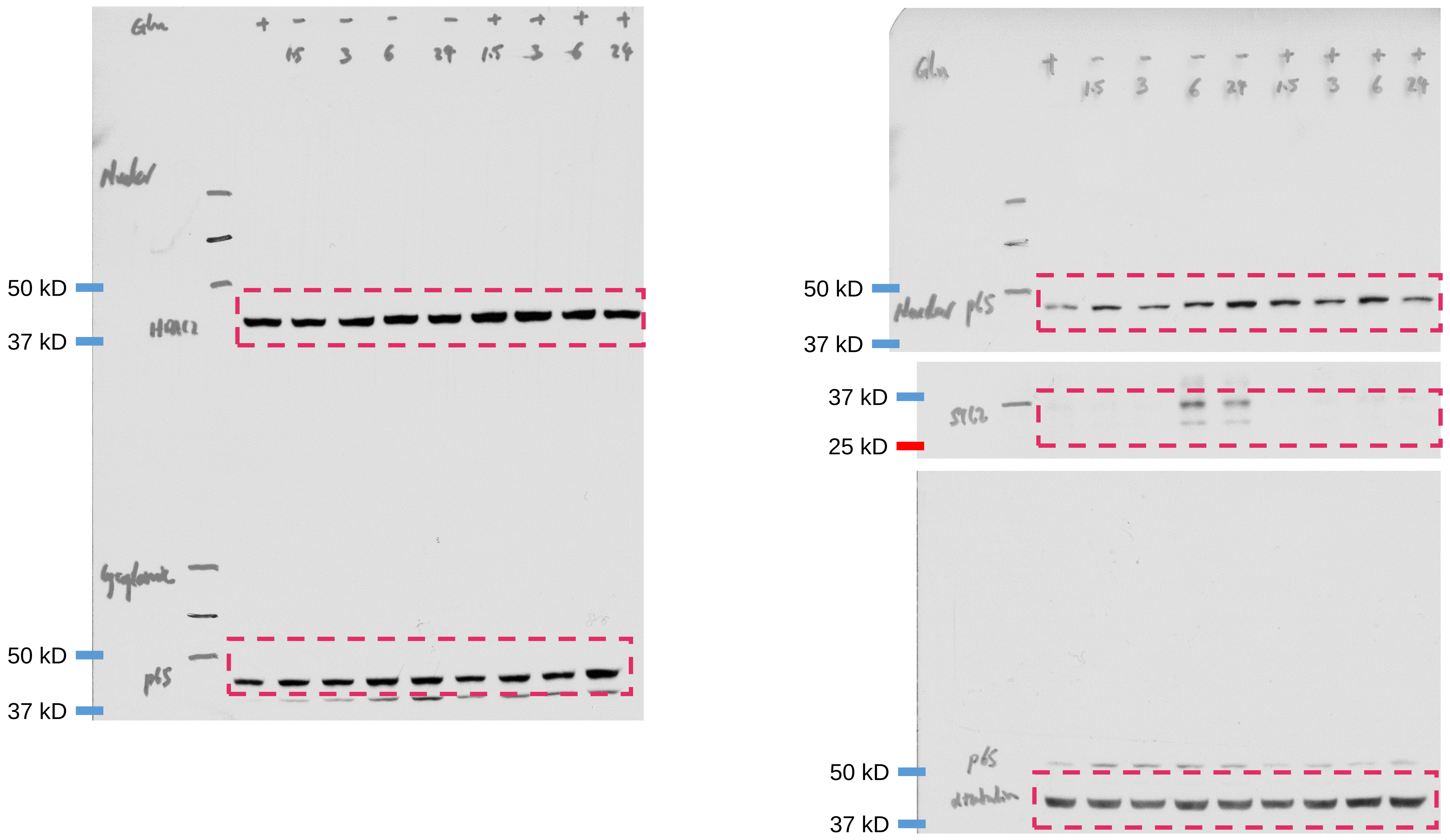

Fig. 2e

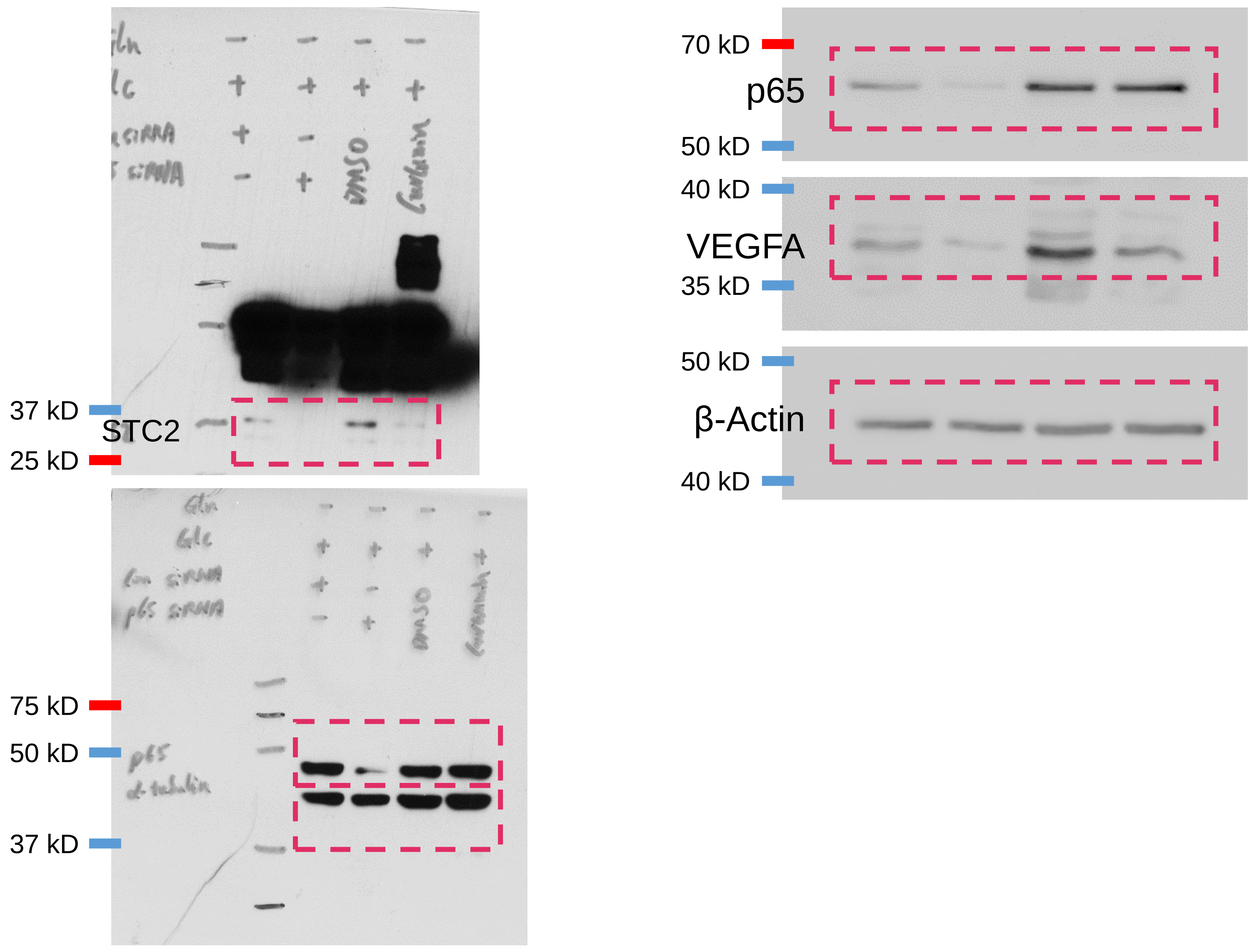

Fig. 2f

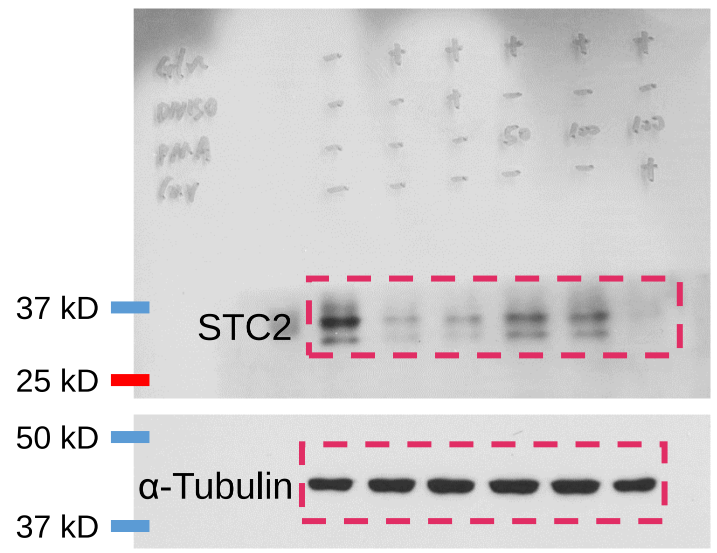

Fig. 2g

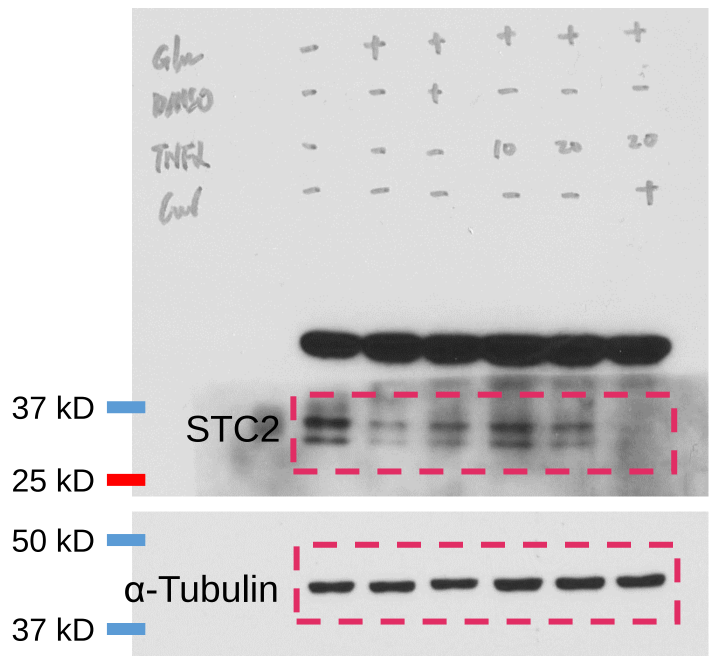

Fig. 2j

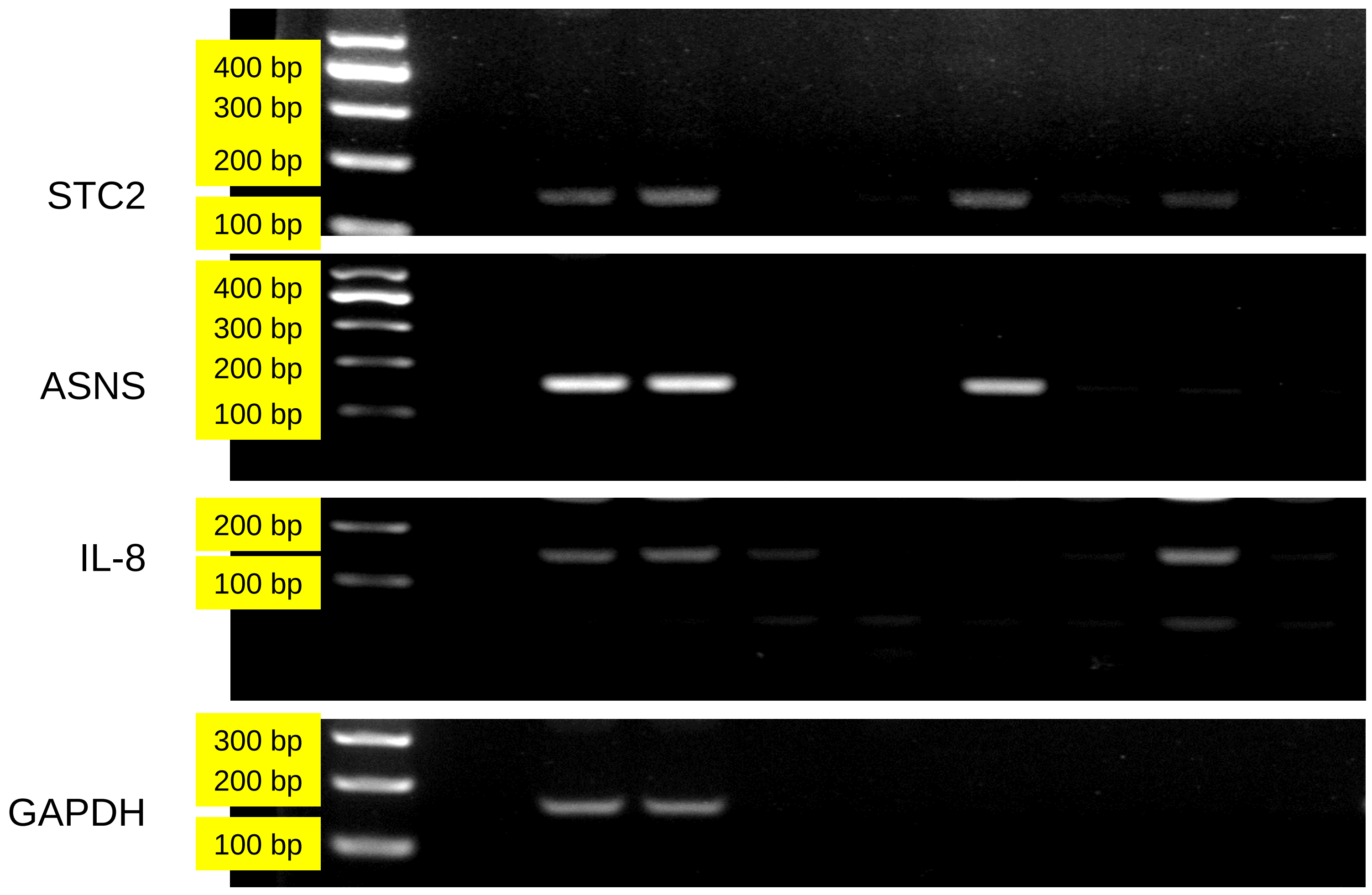

Fig. 3e

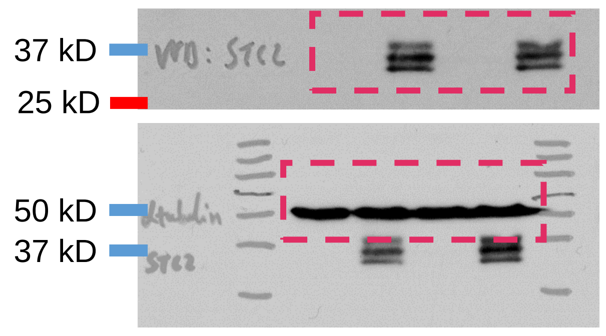

Fig. 3g

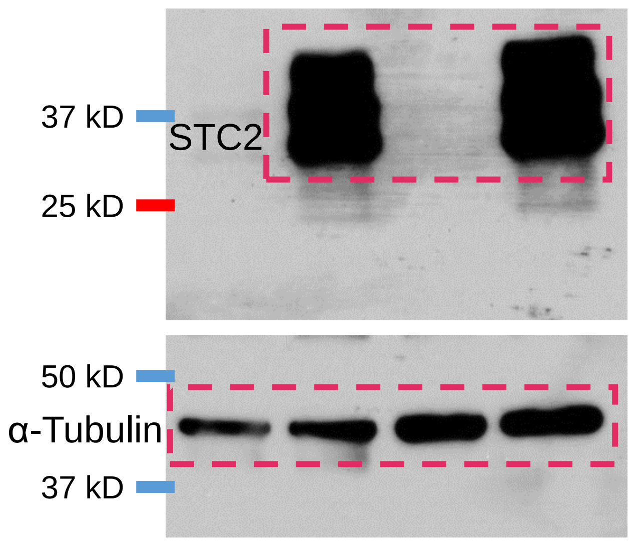

Fig. 4a

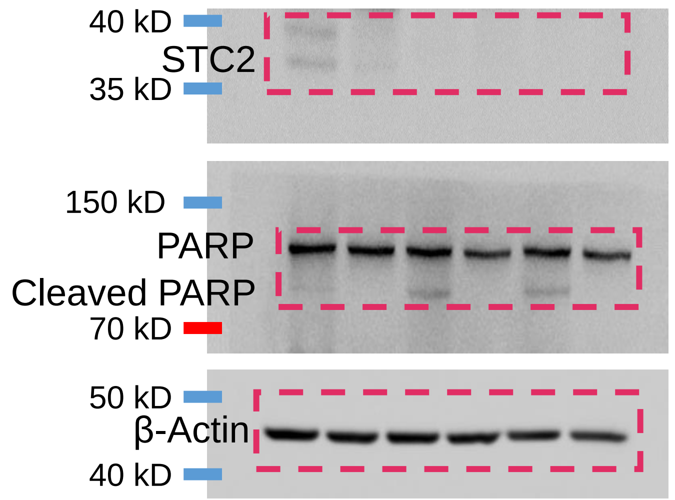

Fig. 4b

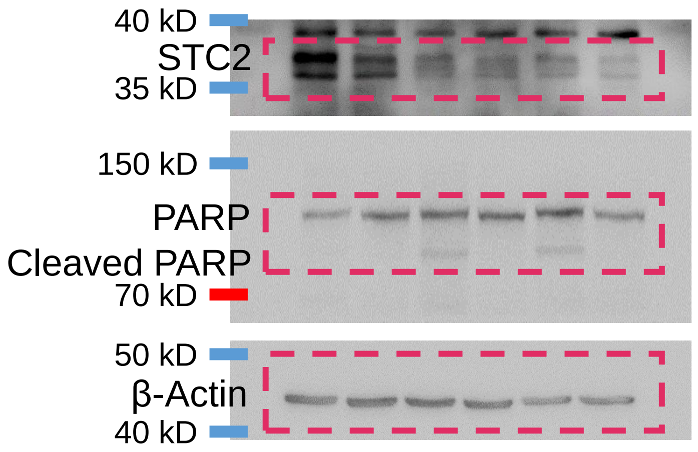

Fig. 4e

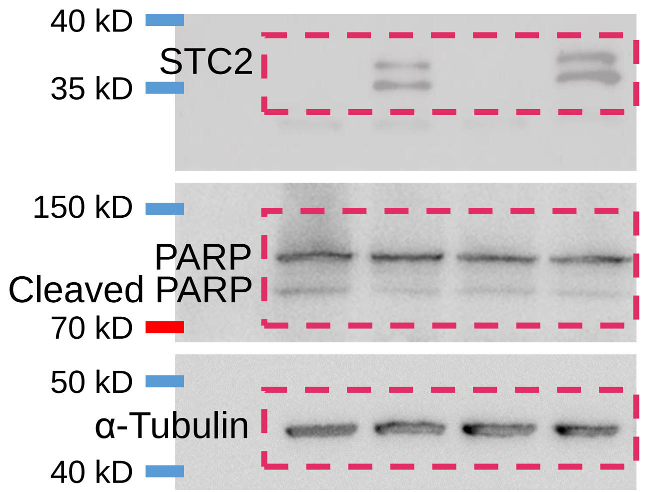

Fig. 4i

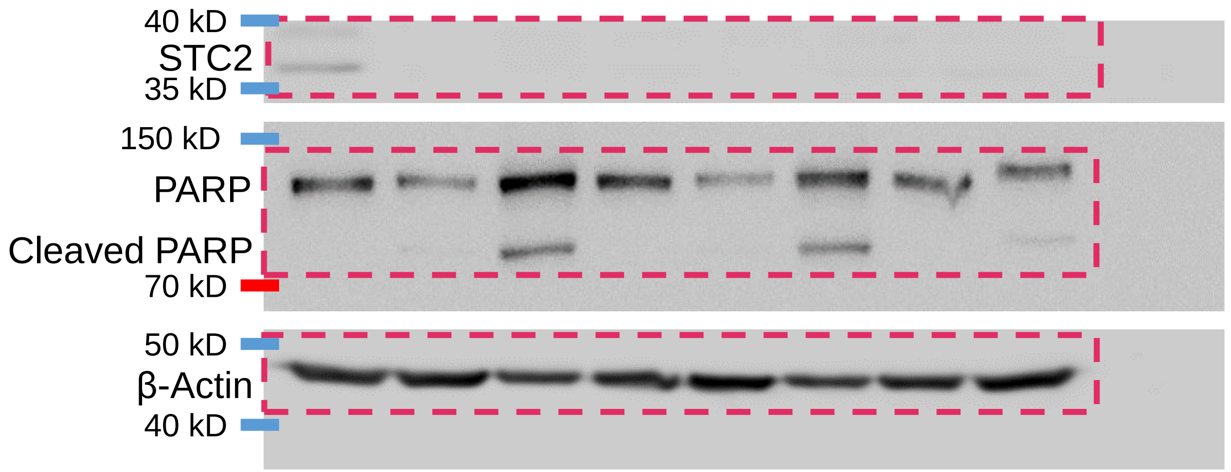

Fig. 4f

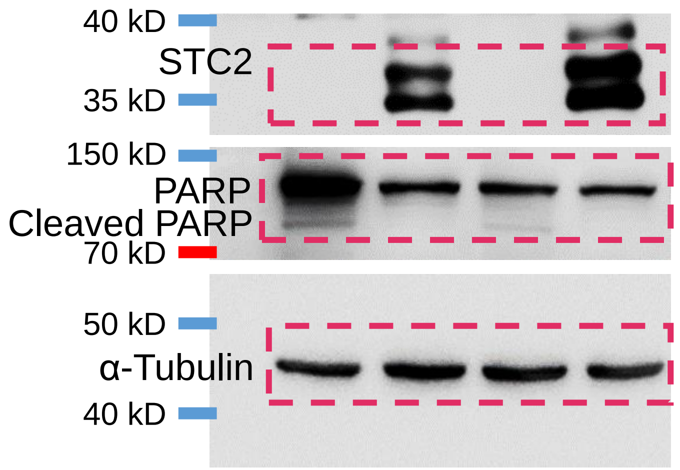

Fig. 4j

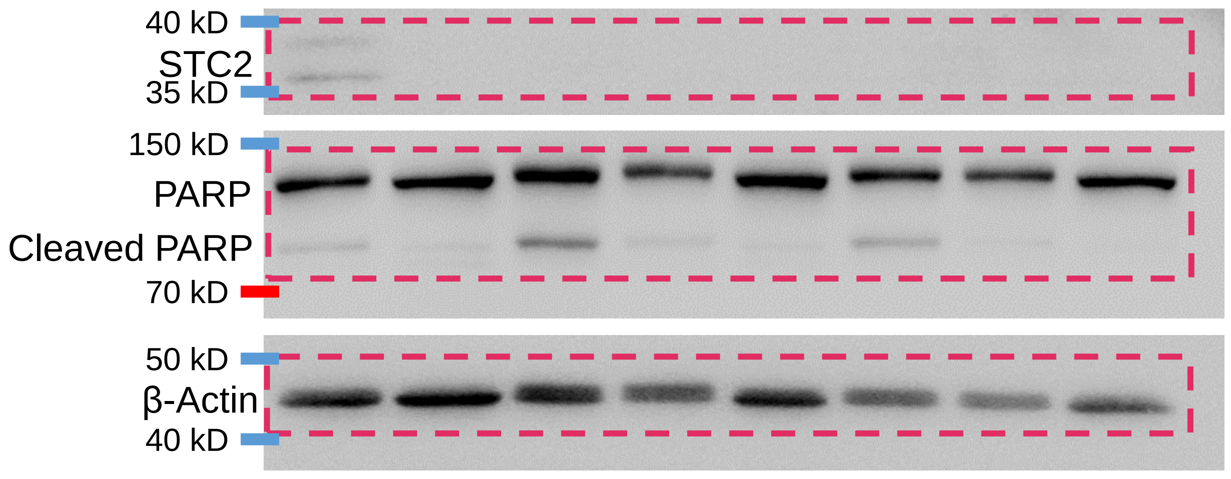

Fig. 7a

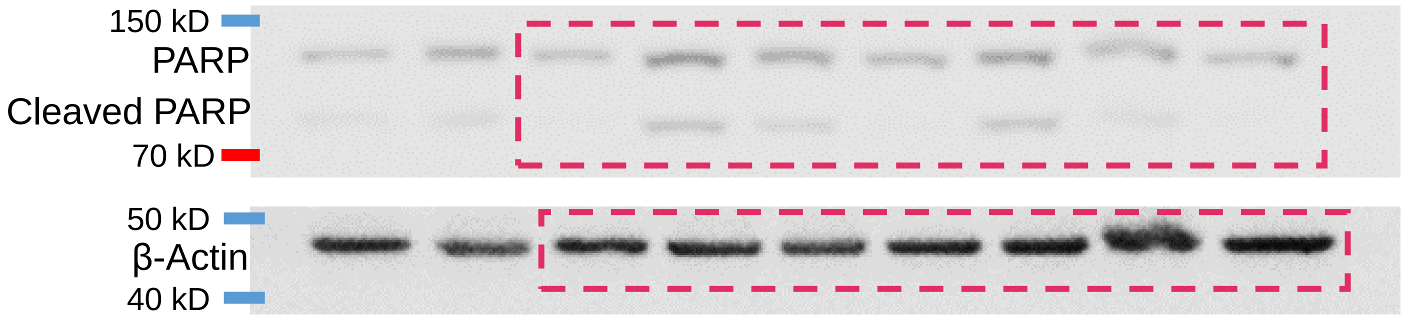

Fig. 7b

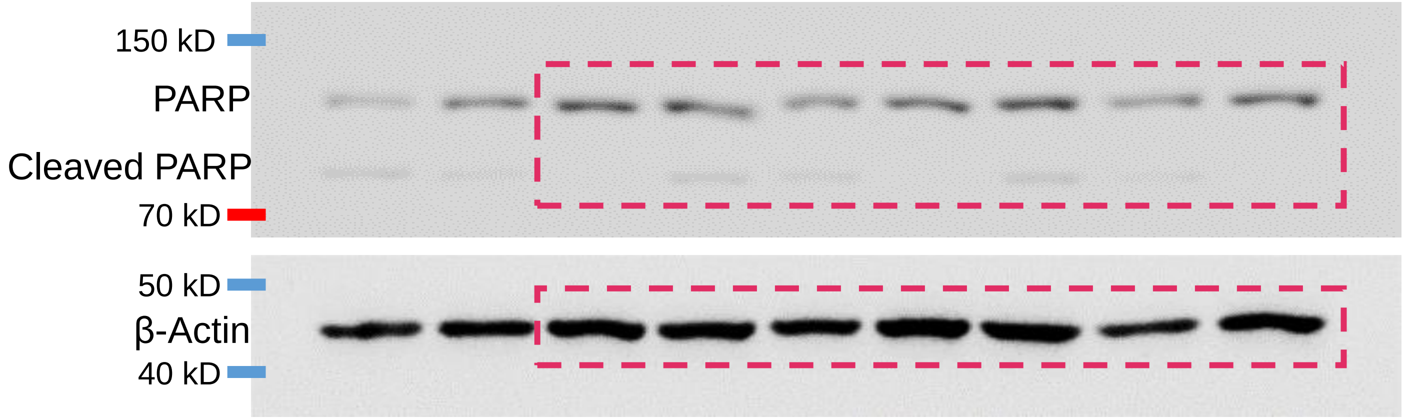

Fig. 7e

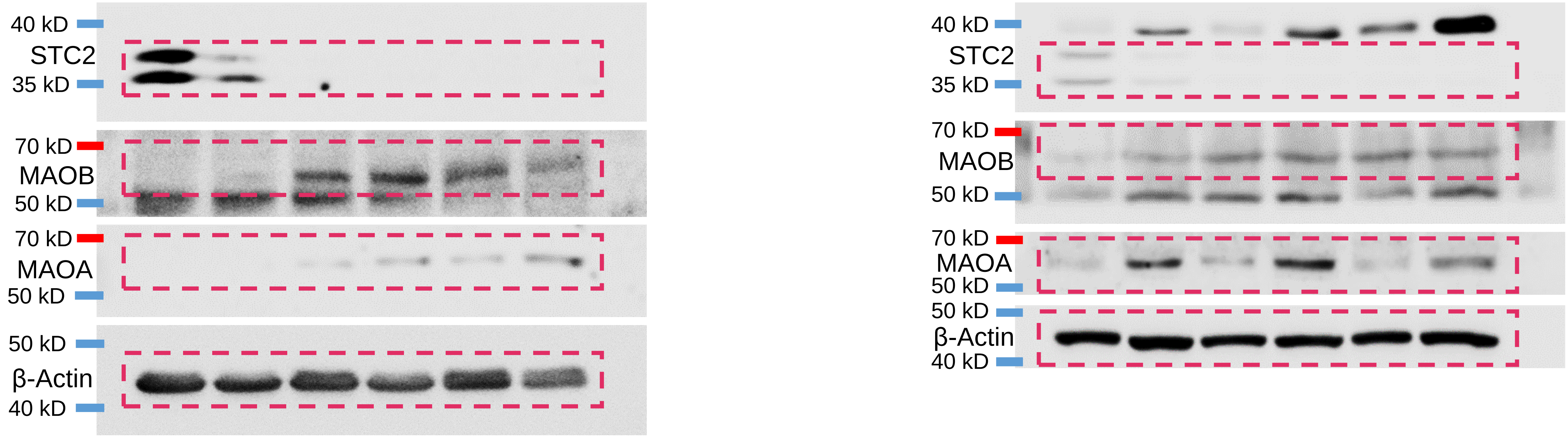

Fig. 7f

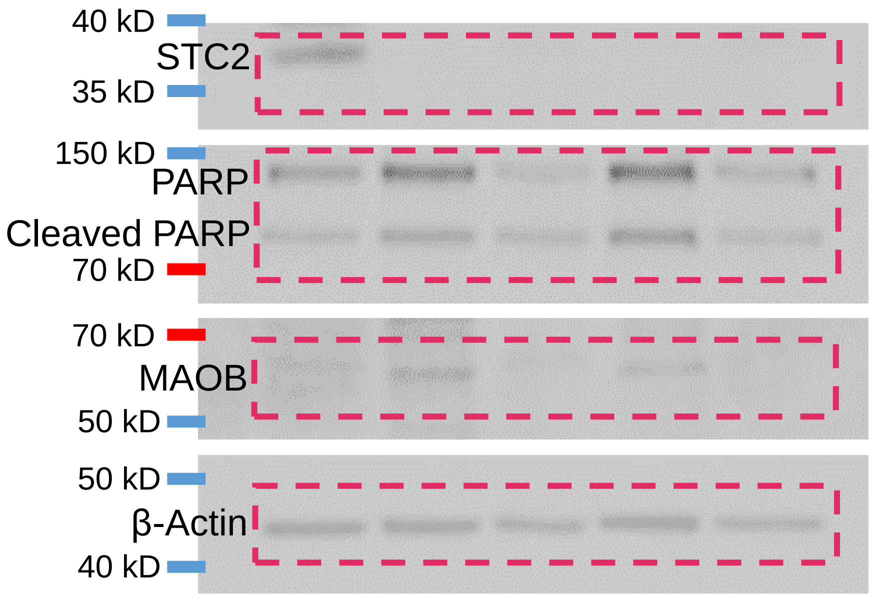

Fig. S1c

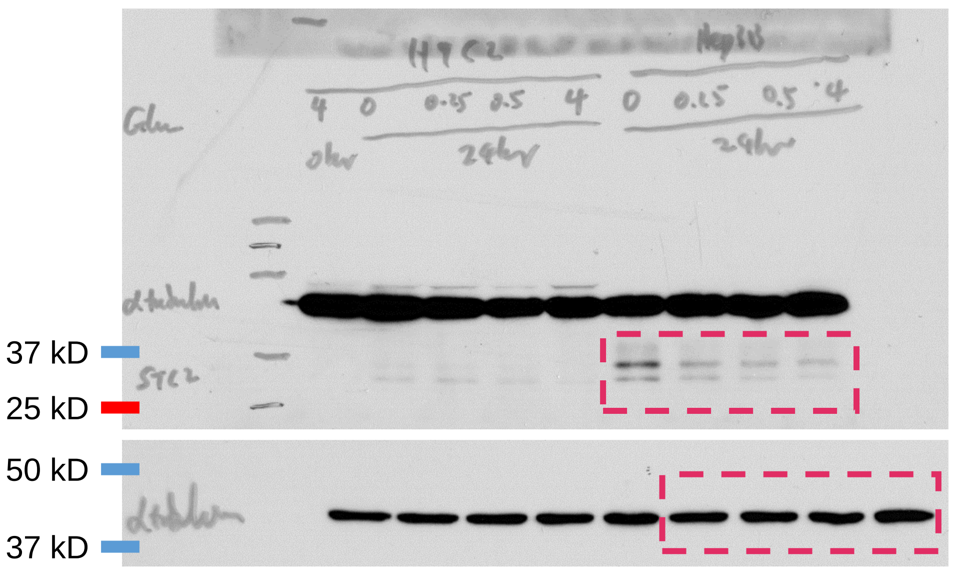

Fig. S1d

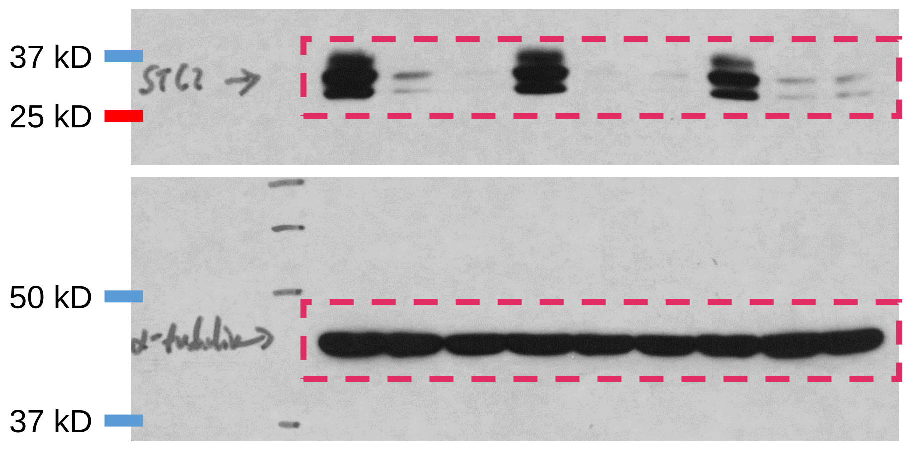

Fig. S1e

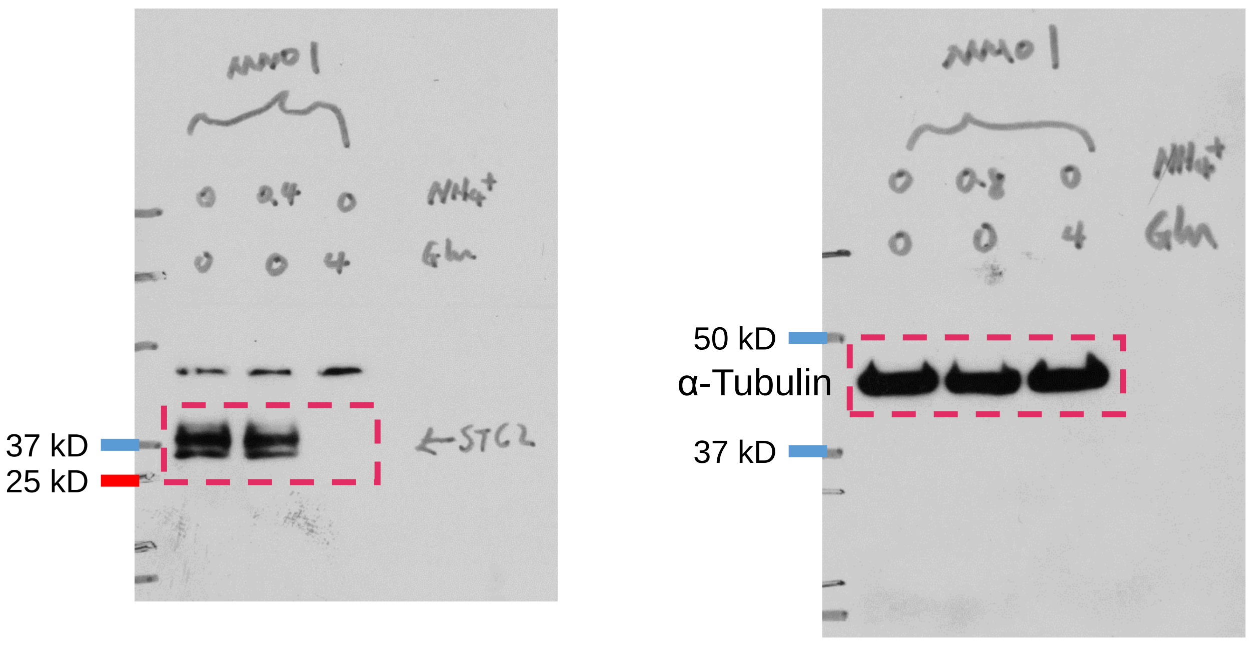

Fig. S1f

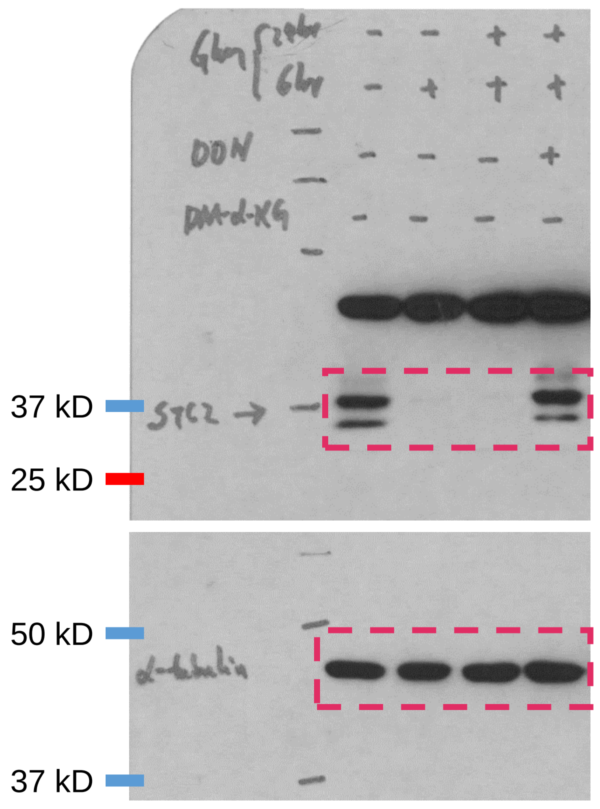

Fig. S1h

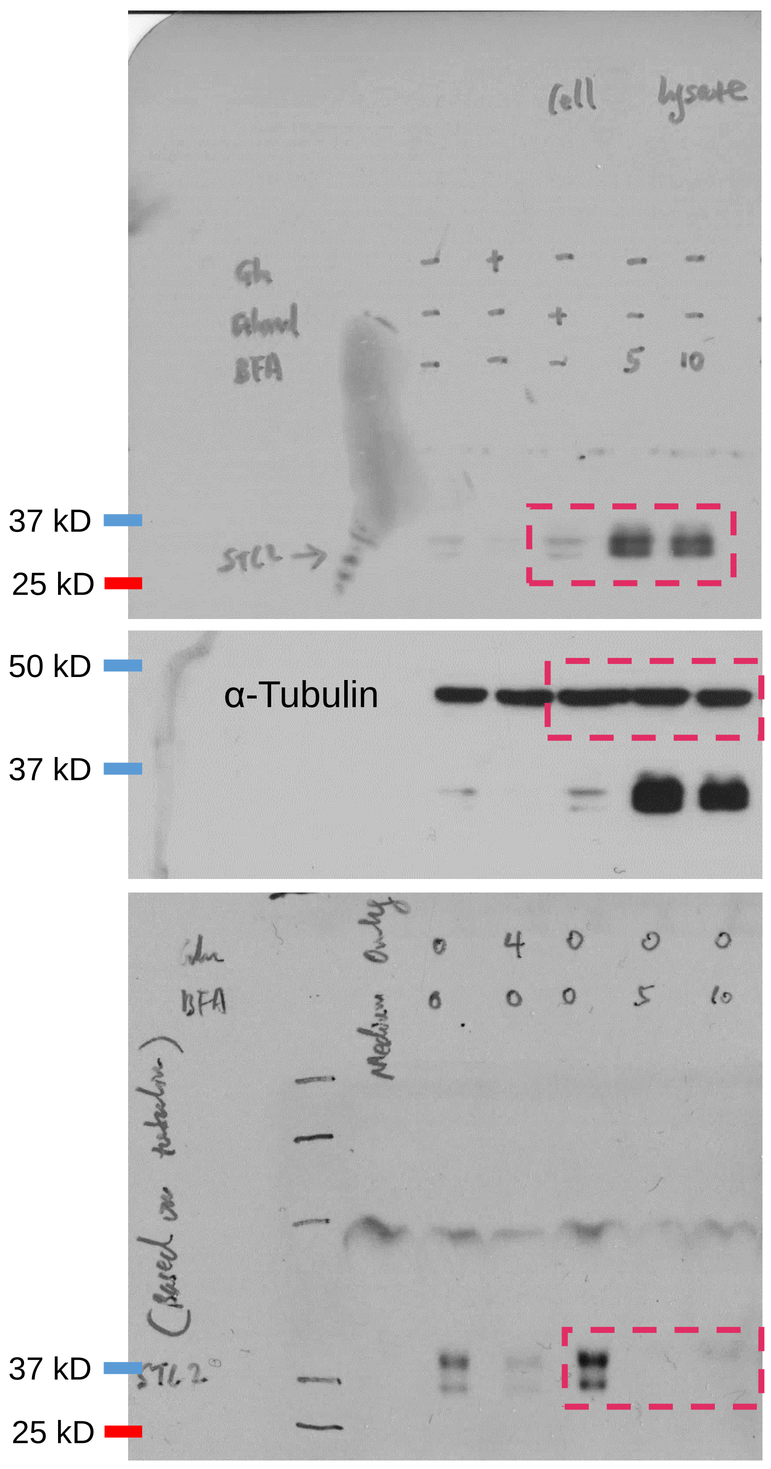

Fig. S2b

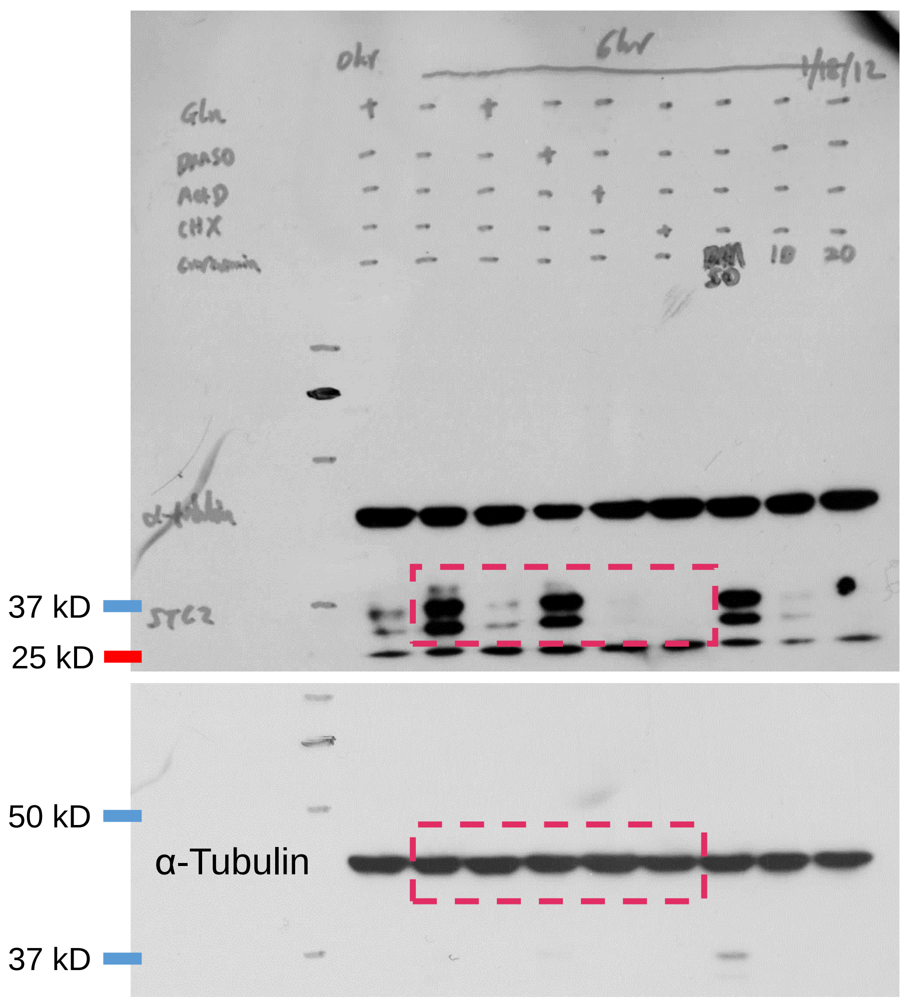

Fig. S2c

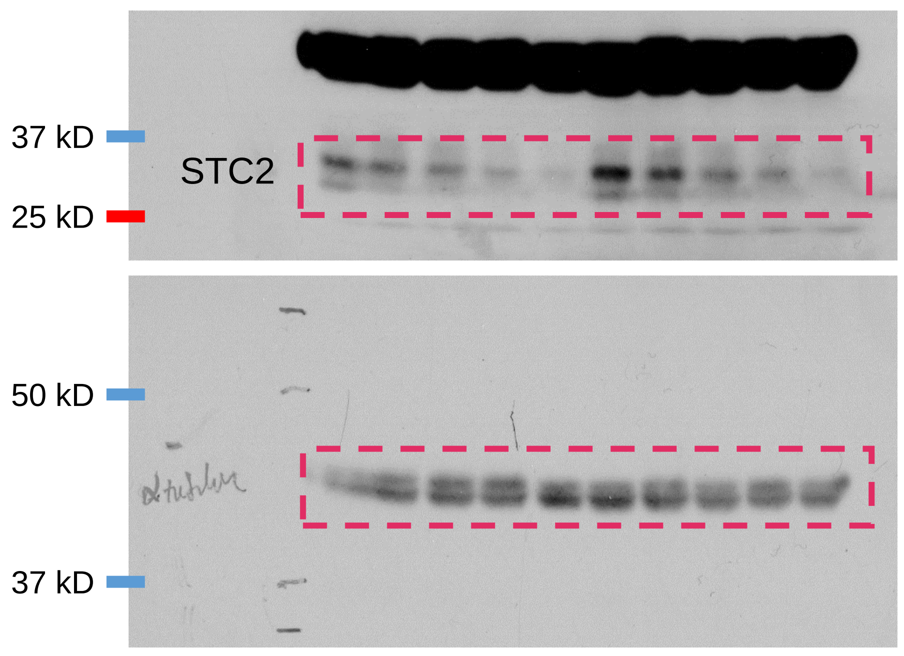

Fig. S2e

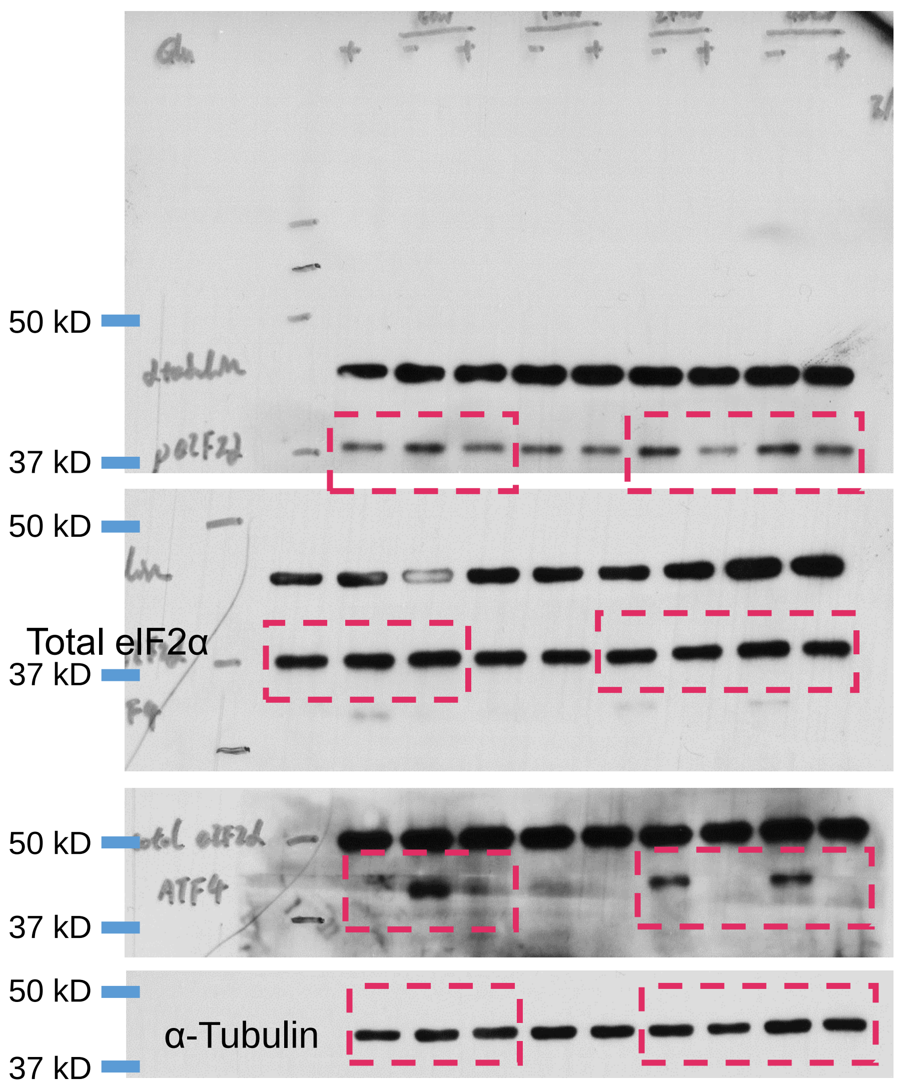

Fig. S2f

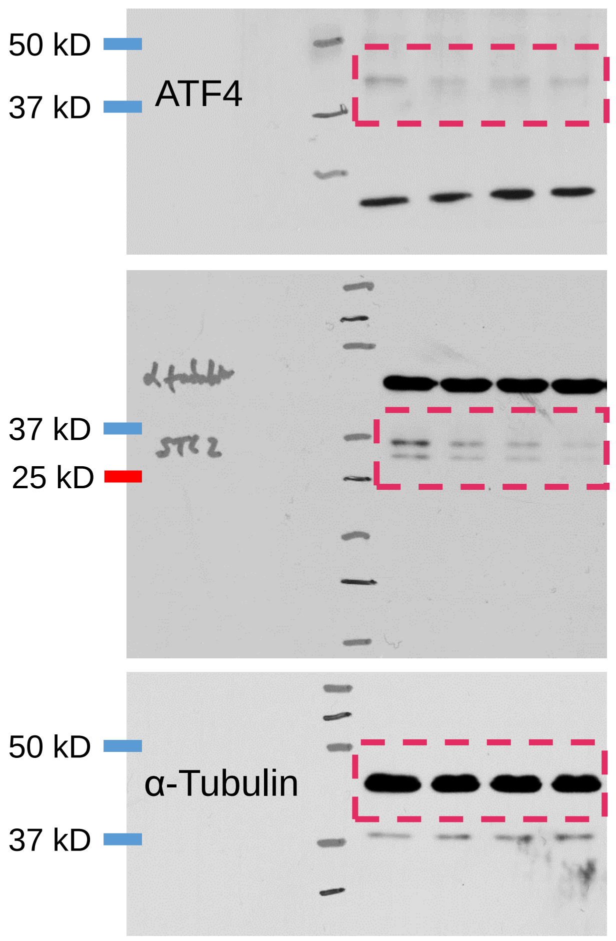

Fig. S2g

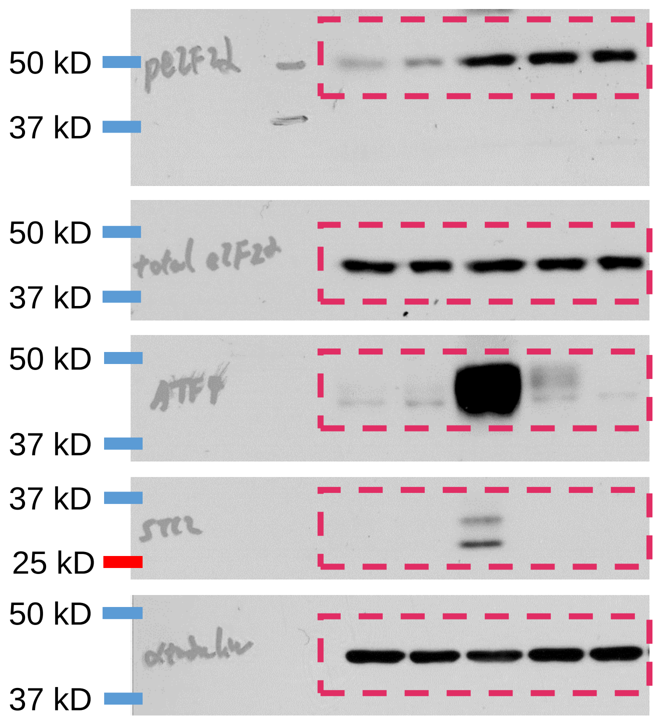

Fig. S2i

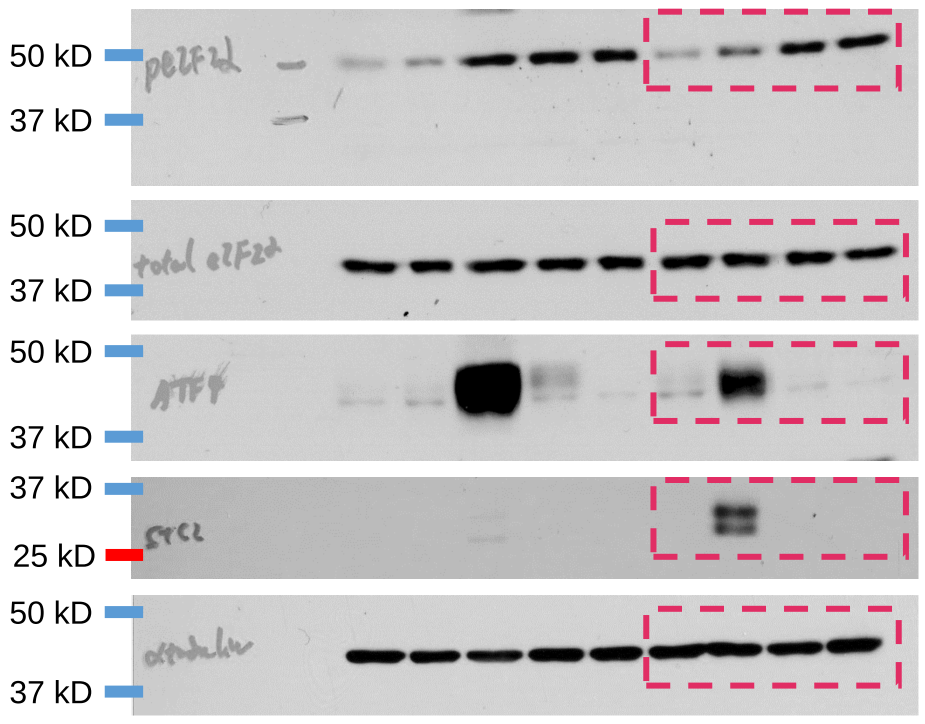

Fig. S2k

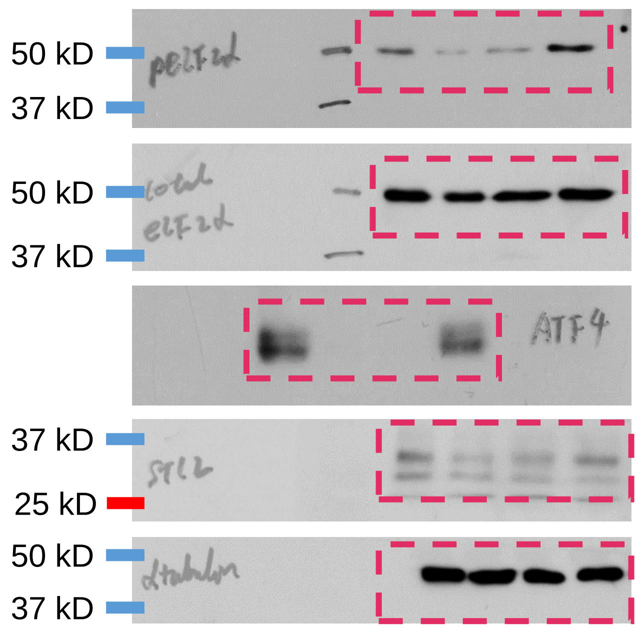

Fig. S2l

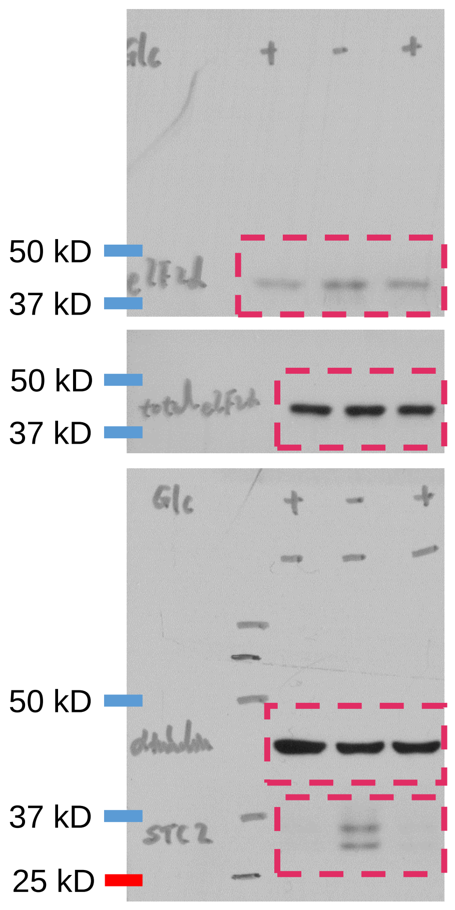

Fig. S3b

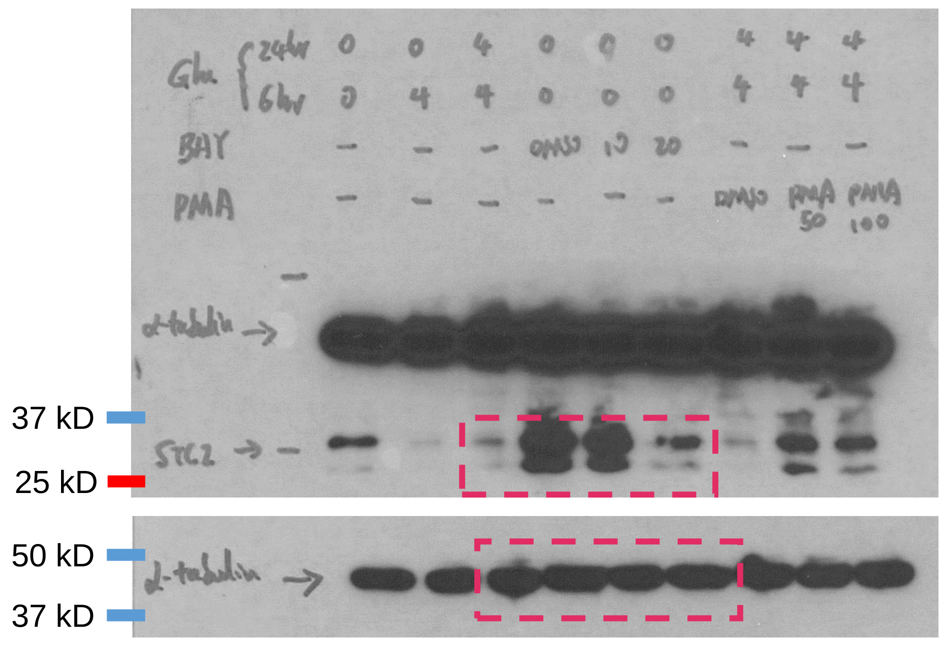

Fig. S3c

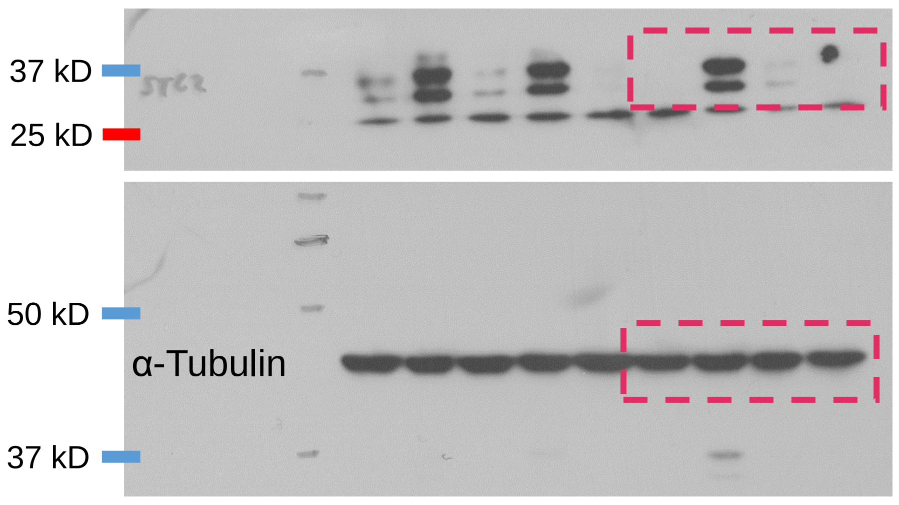

Fig. S3e

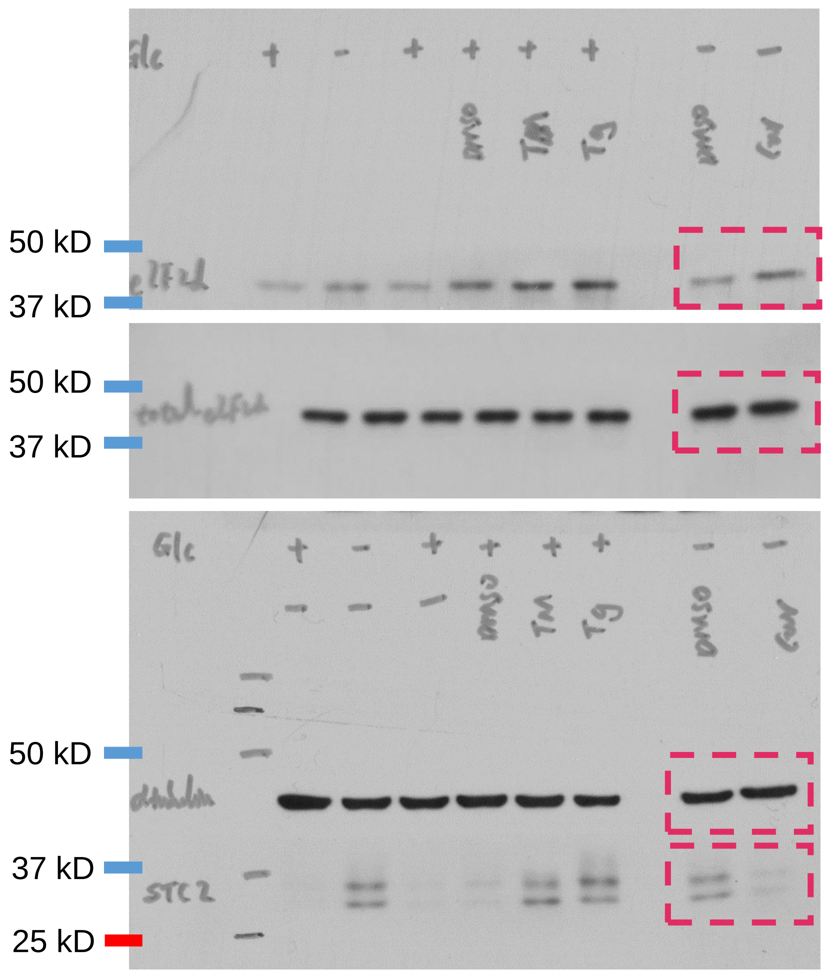

Fig. S3f

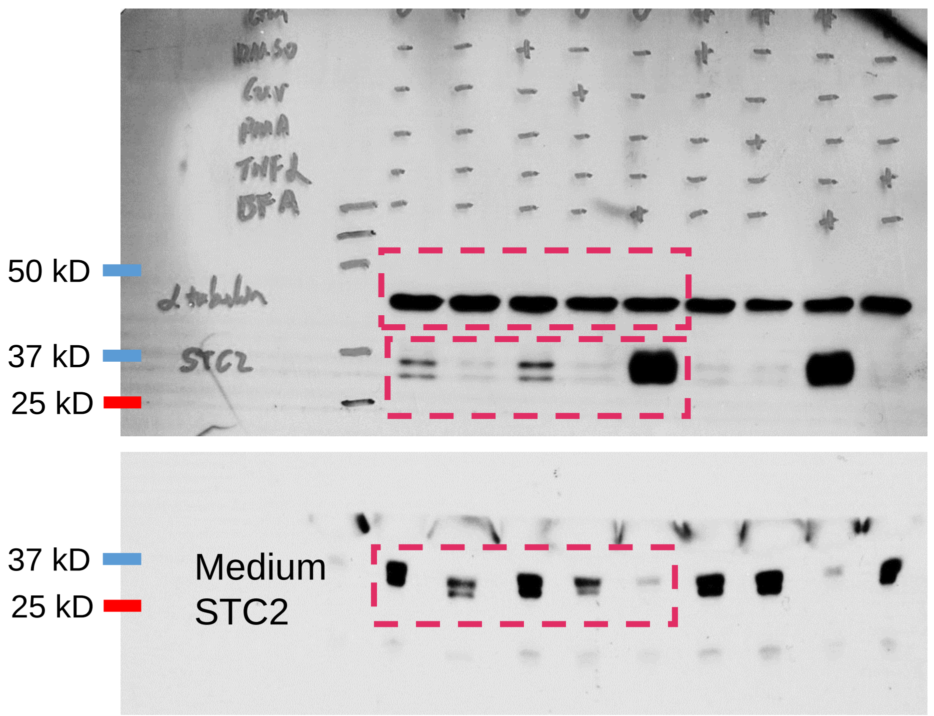

Fig. S3g

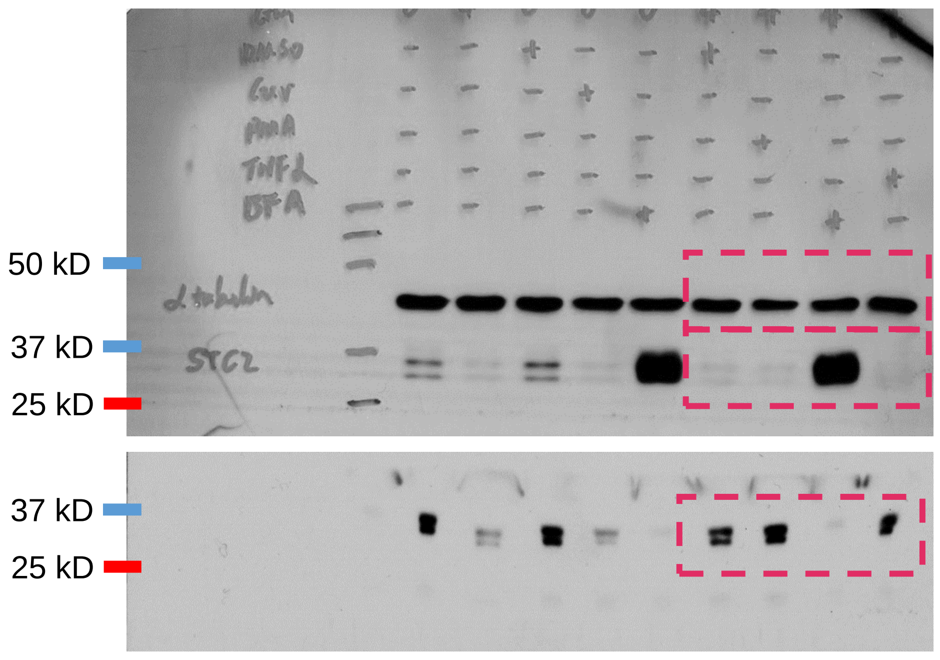

Fig. S3h

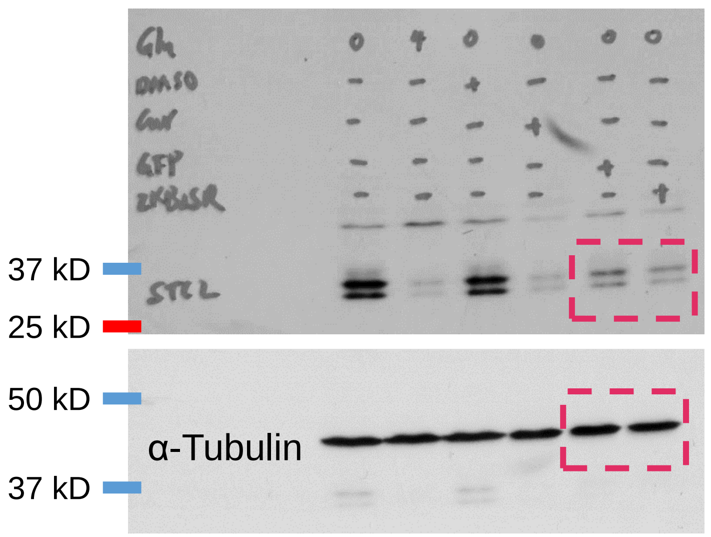

Fig. S5c

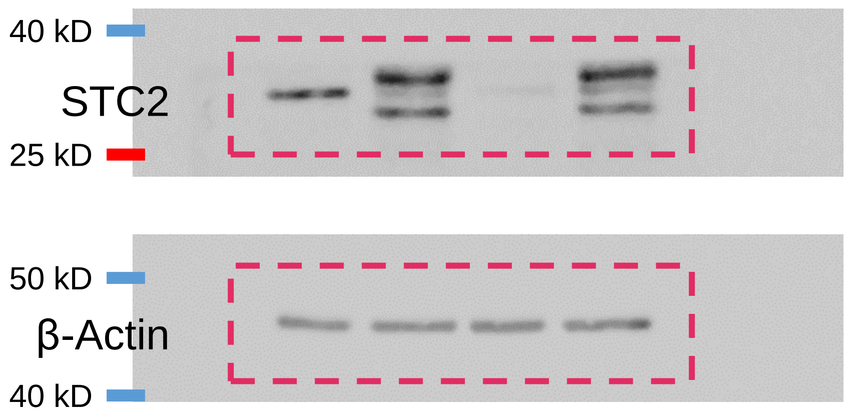

Fig. S6a

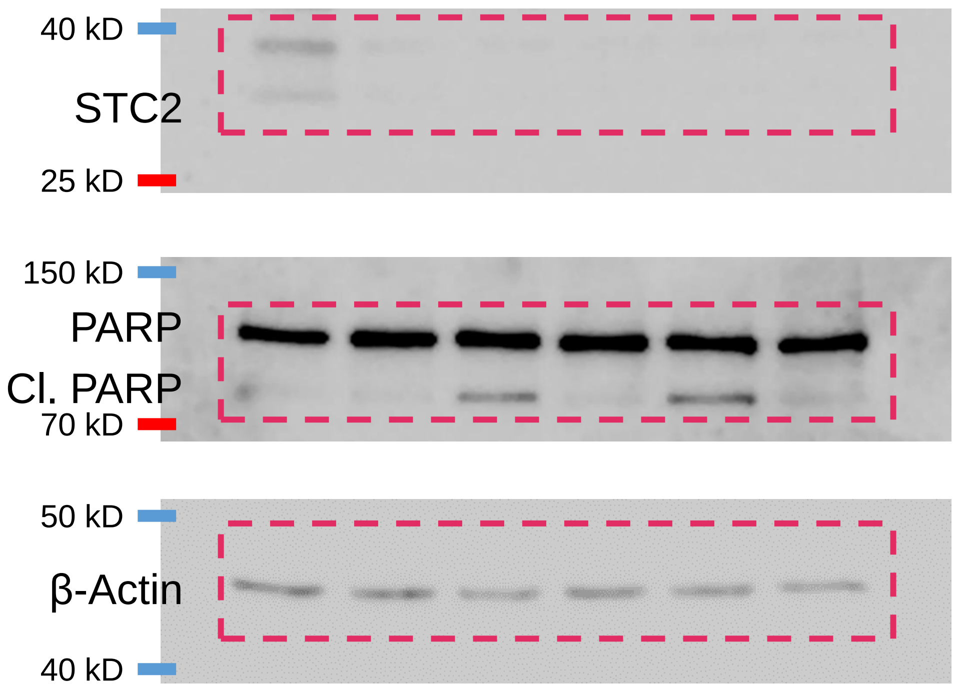

Fig. S6b

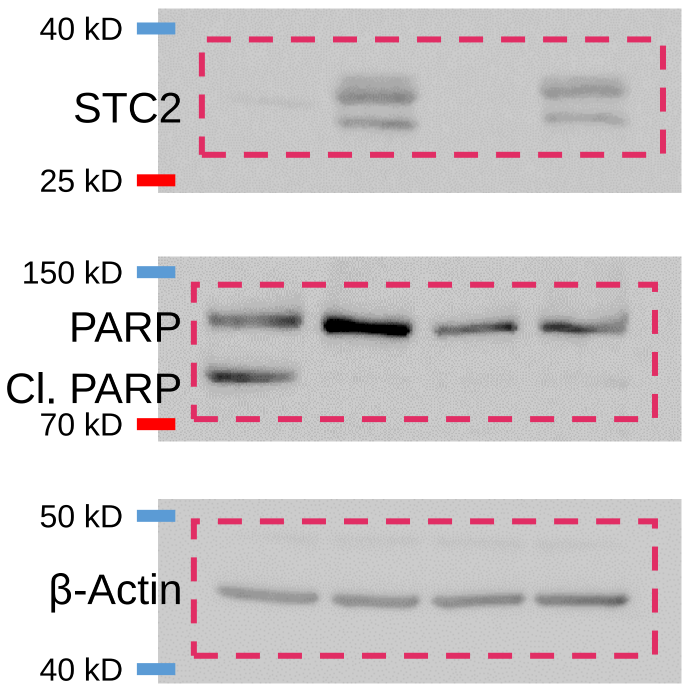

Fig. S6e

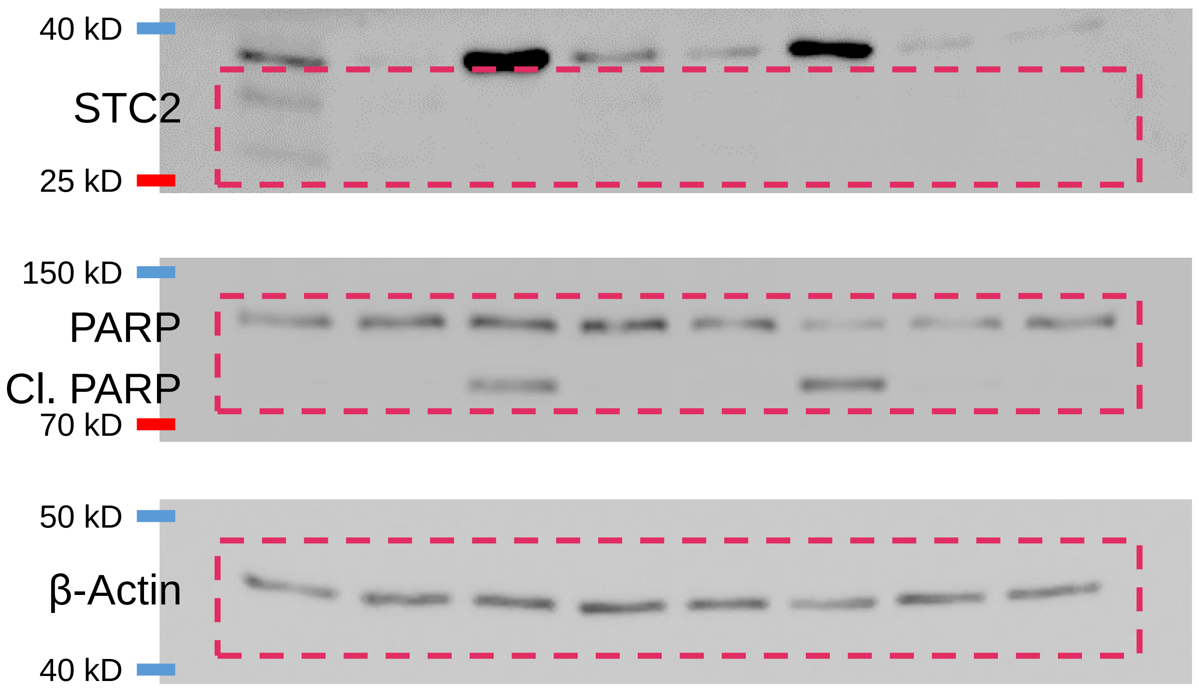

Fig. S14b

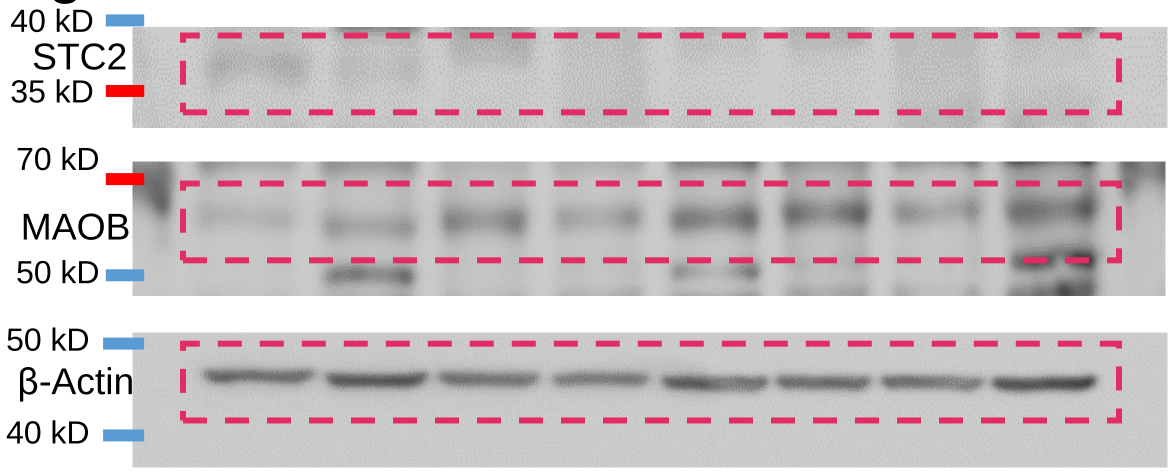

Fig. S14c

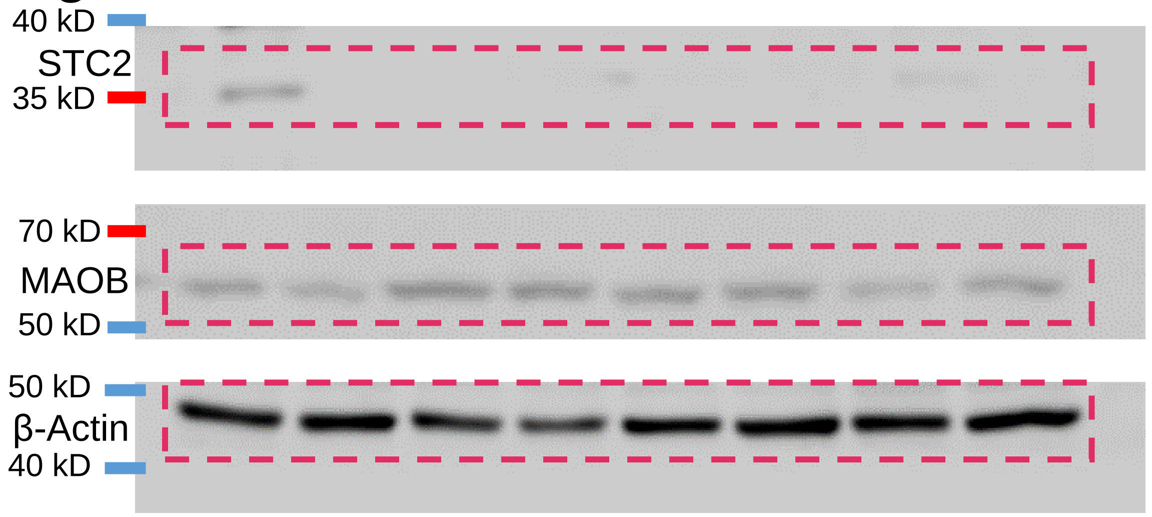

Fig. S15a

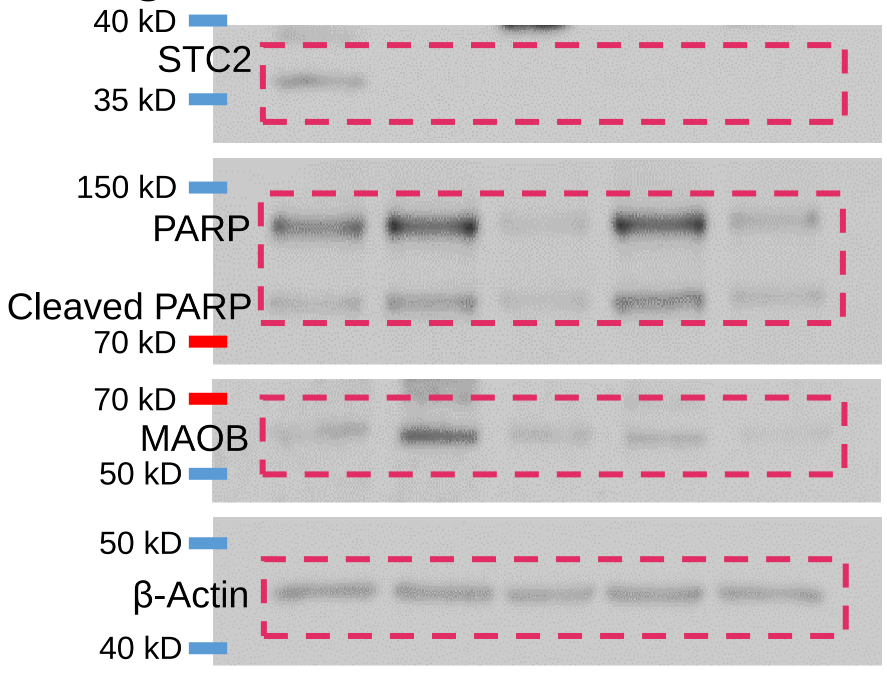

Fig. S16b

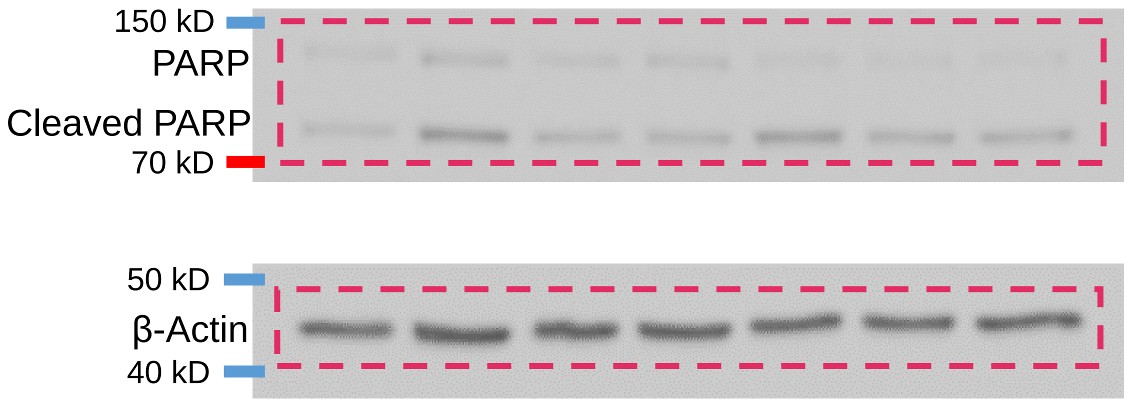

Fig. S16c

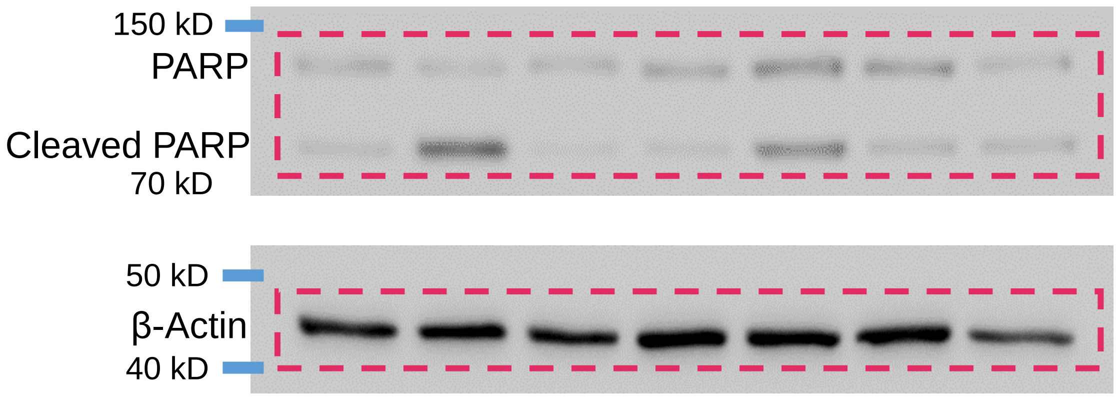

Fig. 5b

Hep3B xenografts

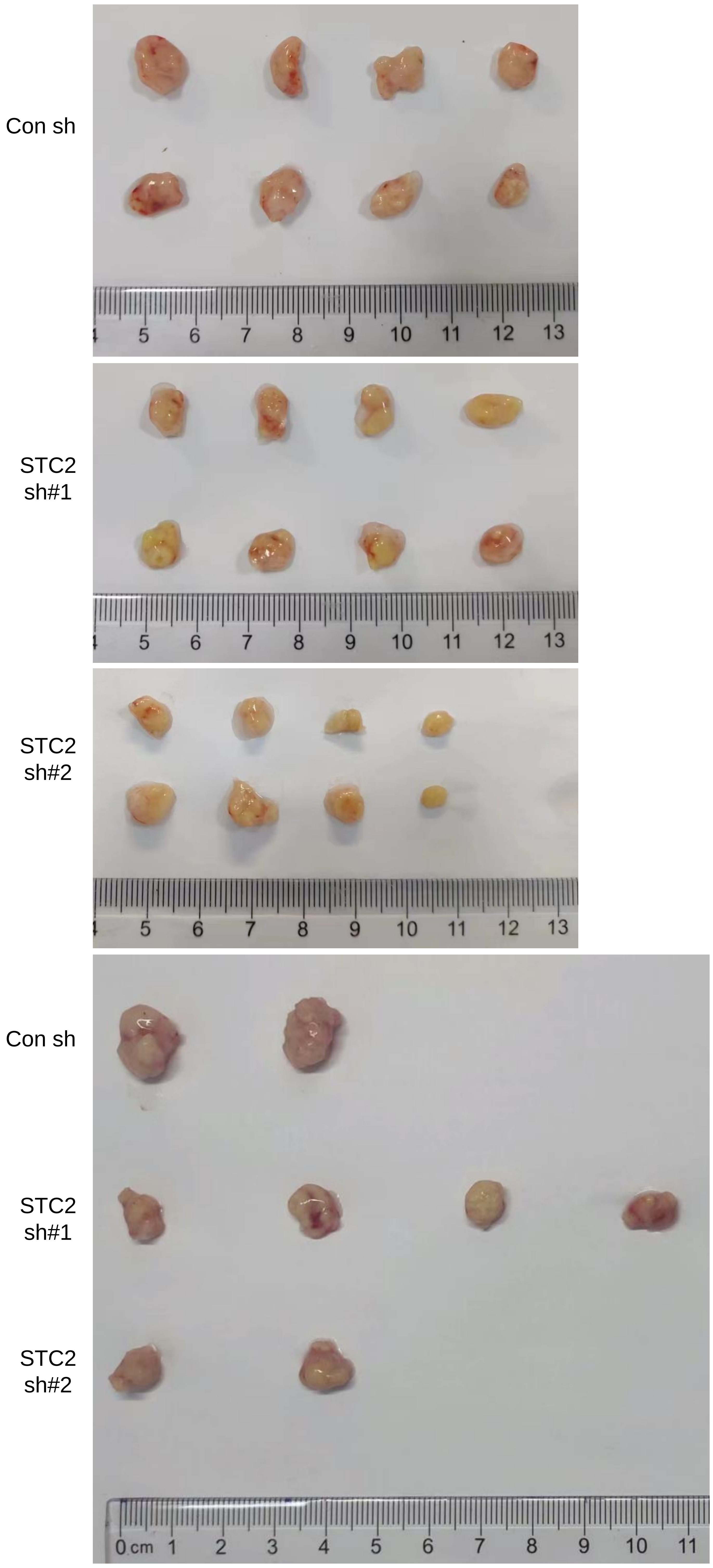

Fig. 5h

HeLa xenografts

Con sh

STC2 sh#1

STC2 sh#2

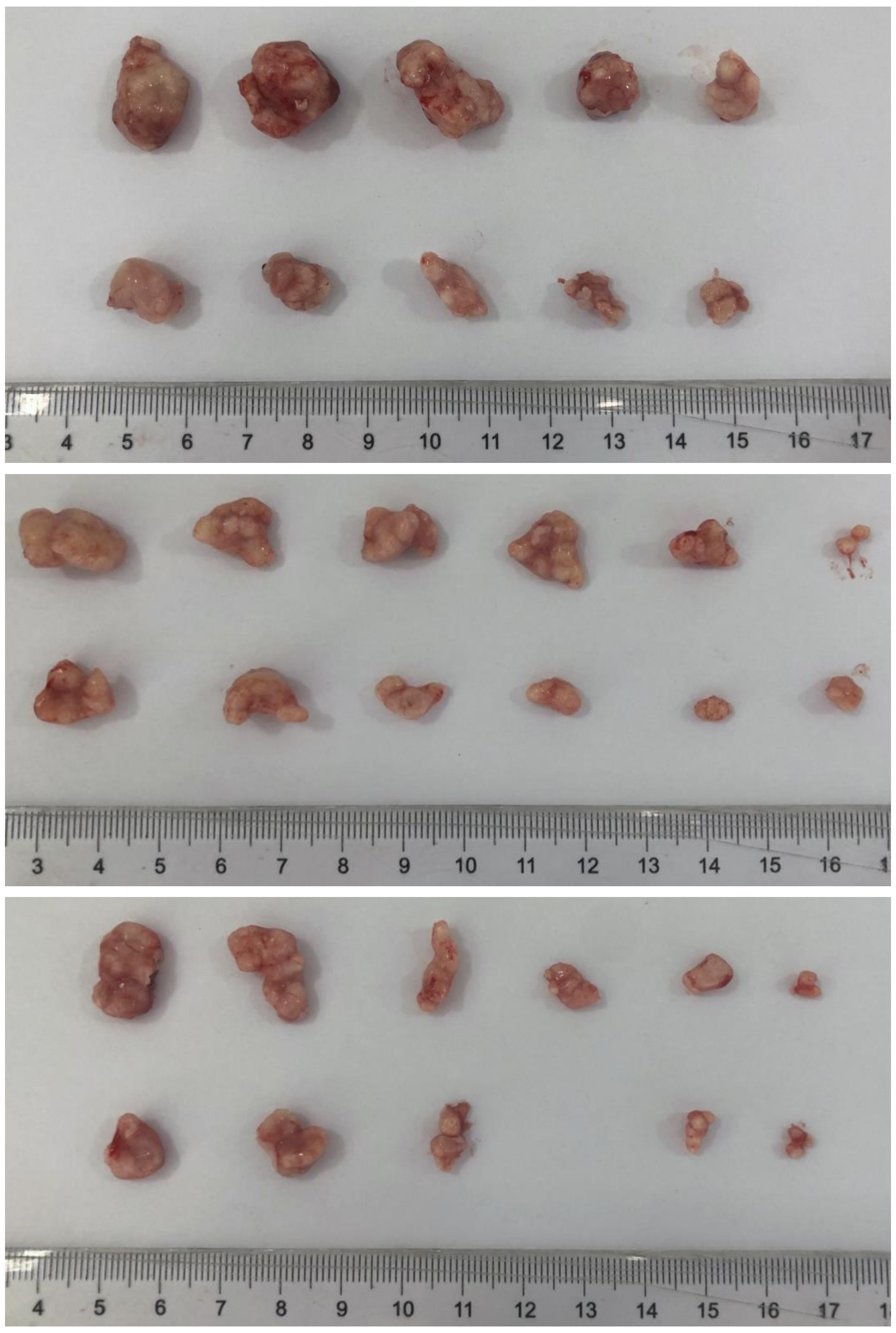

Supplement: Supplementary file 3 — Original Data [file 41419_2024_6961_MOESM3_ESM.pdf]
